# A mitochondrial unfolded protein response inhibitor suppresses prostate cancer growth in mice via HSP60

**DOI:** 10.1172/JCI149906

**Published:** 2022-07-01

**Authors:** Rahul Kumar, Ajay K. Chaudhary, Jordan Woytash, Joseph R. Inigo, Abhiram A. Gokhale, Wiam Bshara, Kristopher Attwood, Jianmin Wang, Joseph A. Spernyak, Eva Rath, Neelu Yadav, Dirk Haller, David W. Goodrich, Dean G. Tang, Dhyan Chandra

**Affiliations:** 1 Department of Pharmacology and Therapeutics,; 2 Department of Pathology and Laboratory Medicine,; 3 Department of Biostatistics,; 4 Department of Biostatistics and Bioinformatics, and; 5 Department of Cell Stress Biology, Roswell Park Comprehensive Cancer Center, Buffalo, New York, USA.; 6 Chair of Nutrition and Immunology and; 7 ZIEL Institute for Food & Health, Technische Universität München, Freising-Weihenstephan, Germany.

**Keywords:** Cell Biology, Oncology, Cell stress, Mitochondria, Prostate cancer

## Abstract

Mitochondrial proteostasis, regulated by the mitochondrial unfolded protein response (UPR^mt^), is crucial for maintenance of cellular functions and survival. Elevated oxidative and proteotoxic stress in mitochondria must be attenuated by the activation of a ubiquitous UPR^mt^ to promote prostate cancer (PCa) growth. Here we show that the 2 key components of the UPR^mt^, heat shock protein 60 (HSP60, a mitochondrial chaperonin) and caseinolytic protease P (ClpP, a mitochondrial protease), were required for the development of advanced PCa. HSP60 regulated ClpP expression via c-Myc and physically interacted with ClpP to restore mitochondrial functions that promote cancer cell survival. HSP60 maintained the ATP-producing functions of mitochondria, which activated the β-catenin pathway and led to the upregulation of c-Myc. We identified a UPR^mt^ inhibitor that blocked HSP60’s interaction with ClpP and abrogated survival signaling without altering HSP60’s chaperonin function. Disruption of HSP60-ClpP interaction with the UPR^mt^ inhibitor triggered metabolic stress and impeded PCa-promoting signaling. Treatment with the UPR^mt^ inhibitor or genetic ablation of *Hsp60* inhibited PCa growth and progression. Together, our findings demonstrate that the HSP60-ClpP–mediated UPR^mt^ is essential for prostate tumorigenesis and the HSP60-ClpP interaction represents a therapeutic vulnerability in PCa.

## Graphical abstract

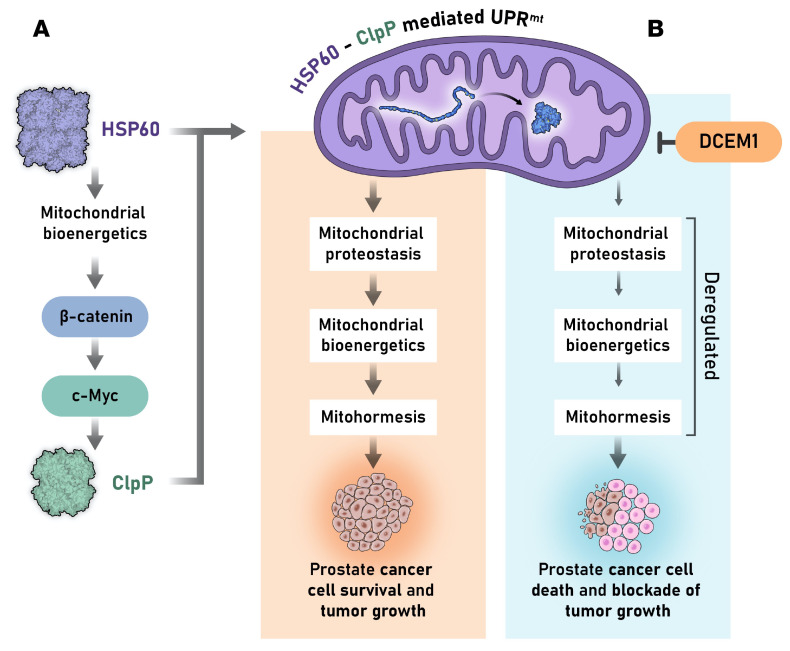

## Introduction

Maintaining protein homeostasis (proteostasis) is essential for normal cellular functions and dysregulated proteostasis has been implicated in many types of cancer (1–3). Proteostasis is regulated by the unfolded protein response (UPR), which is activated in the endoplasmic reticulum and mitochondria to attenuate various cellular stresses (4–6). The mitochondrial UPR (UPR^mt^) facilitates cell adaptation to pervasive mitochondrial stress. The UPR^mt^ activates mitochondria-specific chaperones and proteases to maintain mitochondrial quality control (3, 7–10). Mitochondrial chaperone activity is critical for proper folding of misfolded and unfolded proteins in mitochondria. Two chaperone systems, heat shock protein 60 (HSP60) and mitochondrial HSP70 (mtHSP70), facilitate protein folding function in the mitochondrial matrix (11–16). Interestingly, mtHSP70 cooperates with HSP10 (a cofactor of HSP60) to promote assembly of mature HSP60 complexes (17). More than 26 mitochondrial proteases have been identified in mammalian cells, with LON peptidase 1 (LONP1) and caseinolytic protease P (ClpP) playing prominent roles (6). These proteases degrade unfolded proteins to maintain mitochondrial proteostasis (18). The UPR^mt^ is hyperactive and its components are upregulated in a variety of different cancers (3, 9, 19–22). Genome-wide screening has identified multiple genes involved in protein folding and protein degradation machinery, which are vital for cancer cell survival (23–25). Thus, similar to oncogene addiction (26), the UPR^mt^ may function as a non–oncogene addiction to support cell survival and proliferation (22), but how UPR^mt^ components physically and functionally interact during this cellular response to facilitate tumorigenesis remains poorly understood.

HSP60, encoded by the *HSPD1* gene, is a mitochondrial chaperonin that properly folds nascent or denatured polypeptides (13, 27). HSP60 monomers self-assemble within the mitochondrion to form a tetradecameric barrel, which requires HSP10 for chaperonin activity (11, 12, 28). Damaged or nascent proteins bind to the apical domain of HSP60 within the core of hydrophobic barrel. Binding of HSP10 in the presence of ATP induces a charge turnover within the core of the barrel that leads to a conformational shift, thus initiating protein folding (27, 29). HSP60 is overexpressed in many cancer types, leading to inhibition of cell death, increased metastatic phenotype, and poor survival of patients (19, 30–33). Increased HSP60 in cancer cells maintains mitochondrial proteostasis through the UPR^mt^ (3, 20, 30, 34–36), but whether cancer cell mitochondria require intimate association between HSP60-mediated protein folding machinery and proteases such as ClpP to meet excessive demand of protein turnover during tumorigenesis is not known.

ClpP is a highly conserved mitochondrial serine protease and plays an important role in degradation of unfolded or misfolded proteins. ClpP exists as a heptamer in human mitochondria and relies on the AAA+ chaperone ClpX plus ATP to be proteolytically active (37). ClpP silencing sensitizes cervical carcinoma cells to the chemotherapeutic agent cisplatin by facilitating platinum binding to mtDNA (38). ClpP is upregulated in acute myeloid leukemia (AML) specimens and loss of ClpP decreases viability of AML cells (18). In contrast, ClpP activation induces mitochondrial proteolysis and cancer cell lethality (39). These findings suggest that loss of ClpP promotes accumulation of unfolded proteins, whereas hyperactivation of ClpP may disrupt HSP60-mediated protein folding, both generating chaos in mitochondrial proteostasis and causing mitochondrial dysfunction and impaired cancer cell survival.

Elevated expression of HSP60 correlates with aggressive phenotypes in prostate cancer (PCa) (20). ClpP silencing impairs oxidative phosphorylation (OXPHOS), abolishes metastatic dissemination, reduces cellular proliferation, and induces apoptosis in PCa cells (40). These findings implicate the existence of the UPR^mt^ in cancer such as PCa, but the biological mechanisms underlying how components of the UPR^mt^ cooperate to maintain mitochondrial proteostasis remain unclear. This study proposes that a harmonic relationship between the chaperonin HSP60 and protease ClpP is essential to maintain the increased demand for proteostasis in PCa cell mitochondria. We show that the 2 key components of the UPR^mt^, HSP60 and ClpP, are coordinately upregulated in PCa, are required for optimal mitochondrial function and survival of PCa cells, and promote PCa xenograft growth in vivo. Genetic ablation of *Hsp60* abrogates development and growth of aggressive murine PCa initiated by simultaneous *Pten*, *Rb1*, and *p53* deletion. HSP60 transcriptionally activates ClpP via c-Myc and physically interacts with ClpP in mitochondria via its apical domain. HSP60 promotes β-catenin signaling via maintaining ATP production, leading to c-Myc upregulation. Through in silico screening, we identified a pharmacological inhibitor that binds to the HSP60 apical domain, disrupts HSP60 and ClpP interactions, triggers potent mitochondrial stress, induces robust PCa cell death in vitro, and inhibits tumor growth in vivo irrespective of androgen receptor (AR) status. Together, our findings highlight that the HSP60-ClpP–mediated UPR^mt^ facilitates PCa growth and progression, and identify the HSP60-ClpP interaction as a therapeutic vulnerability in PCa.

## Results

### HSP60 regulates ClpP expression via c-Myc.

**Figure 1:**
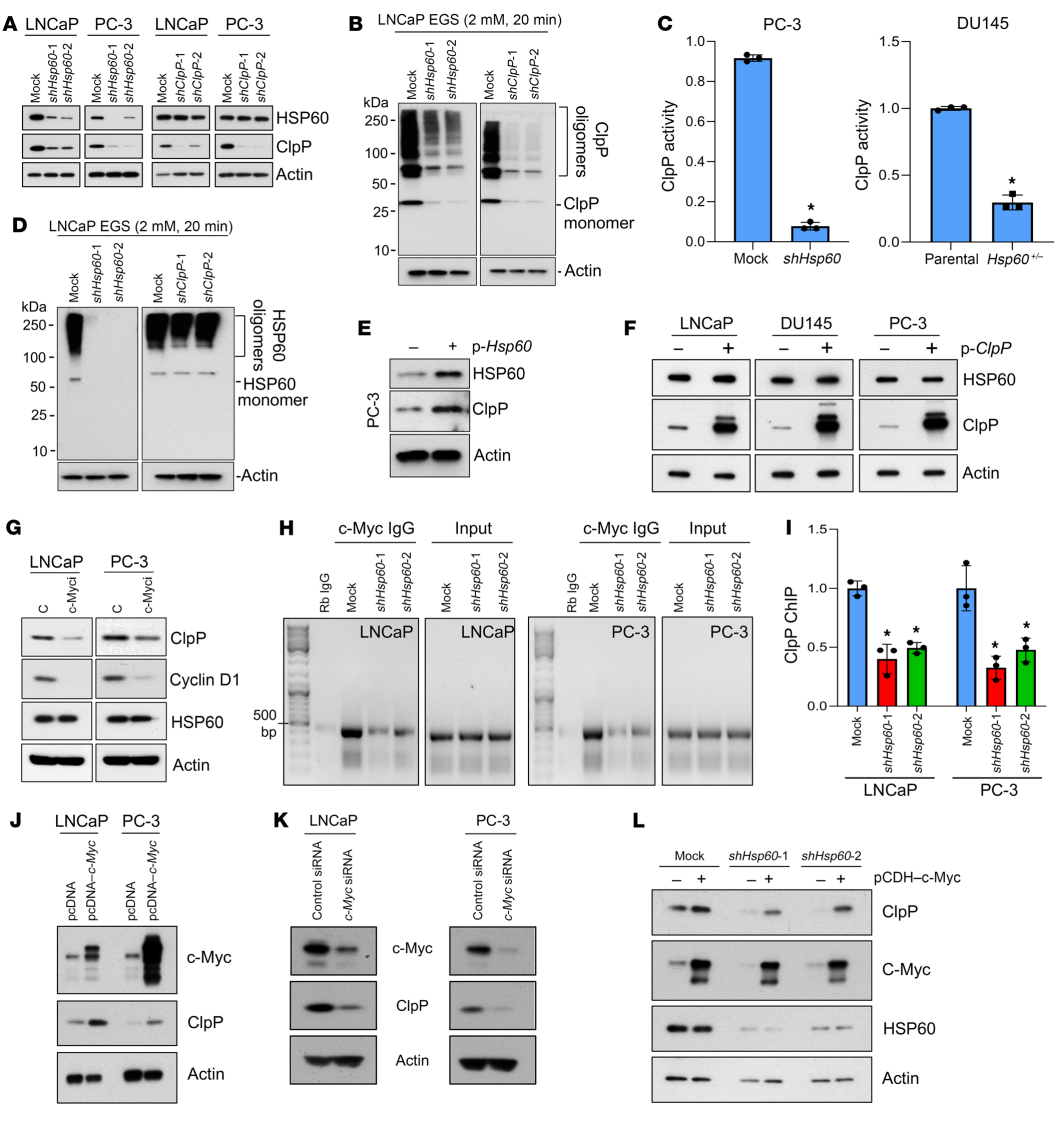
HSP60 regulates ClpP expression and function via c-Myc but not vice versa. (**A**) *Hsp60*- and *ClpP*-silenced LNCaP and PC-3 cells were analyzed for HSP60 and ClpP expression. (**B**) *Hsp60*-and *ClpP*-silenced LNCaP cells were crosslinked with ethylene glycol bis(succinimidyl succinate) (EGS). Protein samples were resolved in an SDS-PAGE gel and probed with an anti-ClpP antibody to analyze its oligomerization status. (**C**) Enzymatic activity of ClpP was assayed from mitochondrial pellets isolated from *Hsp60-*silenced PC-3 cells and *Hsp60^+/–^* DU145 cells. Data are presented as fold change compared to respective controls. (**D**) *Hsp60*- and *ClpP*-silenced LNCaP cells were crosslinked with EGS. Protein samples were resolved in an SDS-PAGE gel and probed with an anti-HSP60 antibody to analyze its oligomerization status. (**E**) HSP60 was overexpressed in PC-3 cells and analyzed for ClpP expression. (**F**) ClpP was overexpressed in LNCaP, DU145, and PC-3 cells and analyzed for HSP60 expression. (**G**) LNCaP and PC-3 cells were untreated (**C**) or treated with c-Myc inhibitor (c-Myci, 10058-F4, 50 μM) for 24 hours. Whole-cell lysates (WCLs) were prepared and analyzed for cyclin D1, ClpP, and HSP60 expression. (**H**) Efficiency of c-Myc binding to the *ClpP* promoter in *Hsp60*-silenced LNCaP and PC-3 cells was determined using a chromatin immunoprecipitation (ChIP) assay. (**I**) Quantitation of the data shown in **H**, represented as fold change compared to mock cells. (**J**) c-Myc was overexpressed in LNCaP and PC-3 cells and analyzed for ClpP expression. (**K**) c-Myc was silenced in LNCaP and PC-3 cells using c-Myc–specific siRNA (100 nM) and analyzed for ClpP expression. (**L**) c-Myc was overexpressed in *Hsp60*-silenced LNCaP cells and analyzed for ClpP expression. Data are mean ± SD. *P < 0.05 by 2-tailed Student’s *t* test (**C**) or 1-way ANOVA followed by Dunnett’s multiple-comparison test (**I**). Actin serves as a loading control.

PCa is a disease of aging prostate tissue that exhibits mitochondrial dysfunction and increased mitochondrial stress (41–43), which activates a protective mechanism in mitochondria termed the UPR^mt^ that is mediated by HSP60 and ClpP. To understand the interplay between HSP60 and ClpP as well as their regulation (Figure 1), we silenced *Hsp60* or *ClpP* in PCa cells. Surprisingly, *Hsp60* silencing greatly reduced ClpP expression (Figure 1A), ClpP oligomerization (Figure 1B), and ClpP activity in PCa cells (Figure 1C). Heterozygous loss of *Hsp60* in the human PCa cell line DU145 also reduced ClpP activity (Figure 1C). By contrast, *ClpP* silencing in PCa cells did not affect HSP60 expression (Figure 1A) or HSP60 oligomerization (Figure 1D). Notably, silencing of *Hsp60* or *ClpP* (Supplemental Figure 1A; supplemental material available online with this article; https://doi.org/10.1172/JCI149906DS1) in PCa cells did not affect expression of LONP1, another mitochondrial matrix–localized ATP-dependent AAA+ protease and a critical factor in mtHSP70 folding machinery (16). *Hsp60* silencing downregulated ClpP expression, but not vice versa, in multiple other cancer cell types (Supplemental Figure 1, B–E).

To establish that HSP60 drives the activation of the UPR^mt^ that leads to increased expression of ClpP, we overexpressed HSP60 in PCa cells and observed elevated expression of ClpP (Figure 1E). However, ClpP overexpression did not alter HSP60 levels in PCa cells (Figure 1F). Interestingly, *Hsp60* silencing also decreased the *ClpP* mRNA levels (Supplemental Figure 1F), suggesting a transcriptional mechanism of ClpP upregulation. Overexpression of FLAG-tagged ClpP in HSP60-deficient PCa cells increased ClpP expression (Supplemental Figure 2), confirming that HSP60 regulates ClpP expression at the transcriptional level.

**Figure 2:**
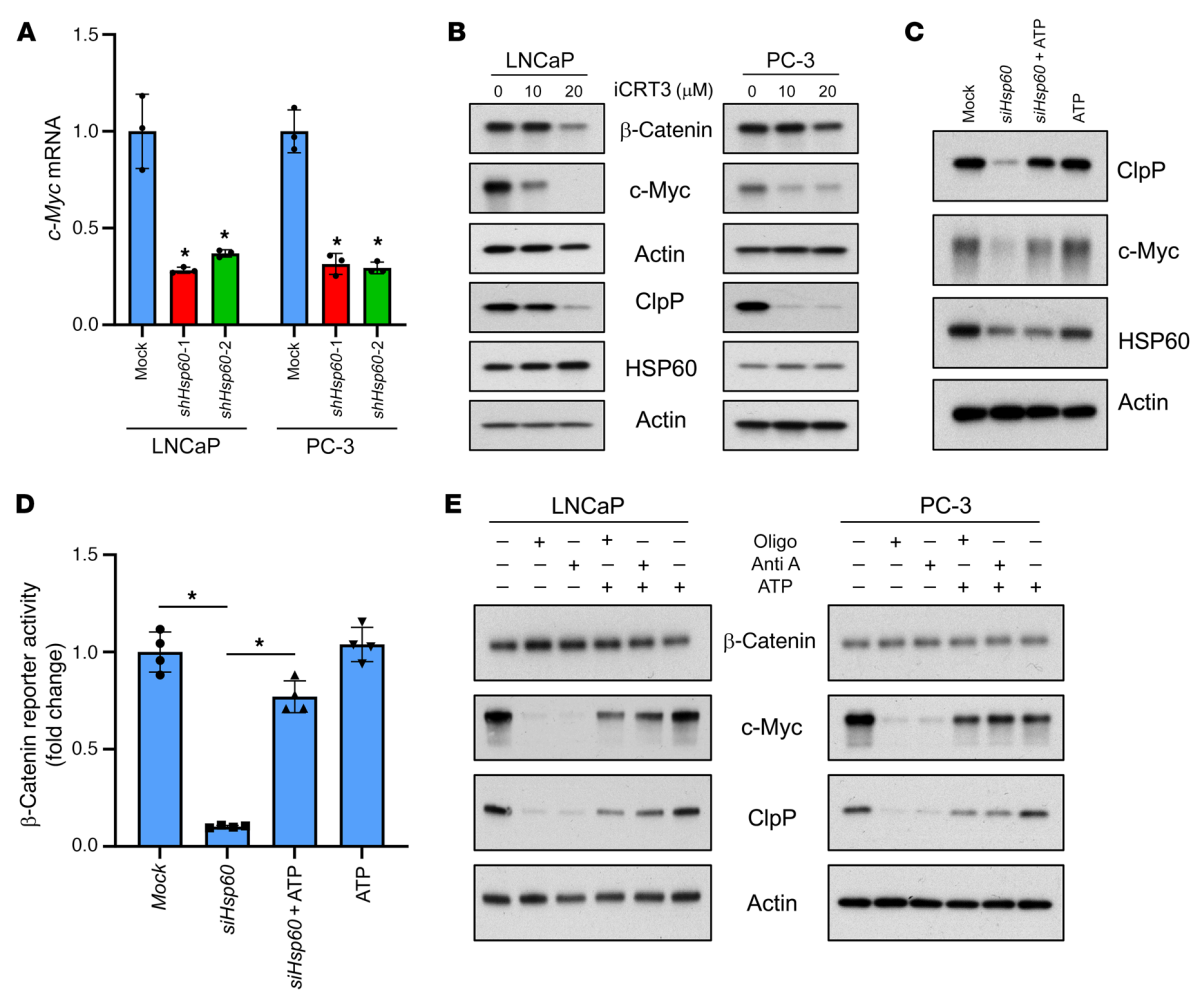
HSP60 regulates c-Myc expression via the β-catenin pathway. (**A**) Analysis of *c-Myc* mRNA expression levels in *Hsp60*-silenced LNCaP and PC-3 cells by real-time PCR using actin mRNA as an internal control. (**B**) Treatment of PCa cells with β-catenin inhibitor iCRT3 for 48 hours downregulated expression of c-Myc and ClpP proteins without any effect on HSP60 protein expression. (**C**) Treatment of *Hsp60*-silenced LNCaP cells with 2 mM ATP for 24 hours rescued the expression of c-Myc and ClpP. (**D**) Assessment of β-catenin promoter reporter activity in *Hsp60*-silenced LNCaP cells; treatment of cells with 2 mM ATP rescued the promoter activity. (**E**) Treatment with mitochondrial OXPHOS complex inhibitors oligomycin (Oligo, 2 μM) and antimycin A (Anti A, 10 μM) for 48 hours downregulated c-Myc and ClpP expression in PCa cells without affecting the expression of β-catenin. Pretreatment of cells with 2 mM ATP rescued the expression of c-Myc and ClpP proteins. Data are mean ± SD. *P < 0.05 by 1-way ANOVA followed by Dunnett’s multiple-comparison test (**A**) or 1-way ANOVA followed by Tukey’s multiple-comparison test (**D**). Actin serves as a loading control.

Importantly, we detected downregulation of *c-Myc* mRNA in *Hsp60*-knockdown PCa cells (Figure 2A), prompting us to investigate whether HSP60 regulates ClpP expression via c-Myc. By searching the ENCODE database, we observed that c-Myc can potentially bind near the *ClpP* gene and regulates its transcription. Indeed, we noticed 2 noncanonical E boxes (44) in the *ClpP* promoter region (Chr 19: 6361236–6361986) (data not shown). Pharmacological inhibition of c-Myc using the compound 10058-F4 (45, 46) downregulated the known c-Myc target cyclin D1 as well as ClpP without affecting HSP60 expression (Figure 1G). To validate that c-Myc regulates ClpP expression in PCa cells, we performed a chromatin immunoprecipitation (ChIP) assay with an anti–c-Myc antibody, and observed reduced c-Myc binding to the *ClpP* promoter upon *Hsp60* silencing (Figure 1, H and I). Consistently, c-Myc overexpression upregulated (Figure 1J), whereas c-Myc silencing reduced (Figure 1K), ClpP expression. To further corroborate that HSP60 regulates ClpP expression via c-Myc, we overexpressed c-Myc in *Hsp60*-knockdown PCa cells and observed the restoration of ClpP expression in HSP60-deficient PCa cells (Figure 1L). *ClpP* promoter reporter assays also revealed that c-Myc knockdown decreased the *ClpP* promoter activity, whereas c-Myc overexpression increased the *ClpP* promoter activity (Supplemental Figure 1G). Together, these data indicate that HSP60 regulates ClpP expression via c-Myc.

### HSP60 regulates c-Myc via ATP-dependent β-catenin signaling to promote ClpP expression.

c-Myc expression is regulated by β-catenin (47, 48) and mitochondrial ATP production is crucial to maintain β-catenin signaling (49, 50). We hypothesized that HSP60 may regulate c-Myc and subsequent ClpP expression by ATP-dependent β-catenin signaling. Treatment of PCa cells with the β-catenin inhibitor iCRT3 downregulated c-Myc and ClpP expression without modulating HSP60 expression (Figure 2B). Treatment of *Hsp60*-knockdown PCa cells with exogenous ATP rescued β-catenin transcriptional activity along with restoration of c-Myc and ClpP expression (Figure 2, C and D). To further confirm the role of mitochondrial ATP production in β-catenin signaling, we treated PCa cells with the mitochondrial OXPHOS inhibitors oligomycin and antimycin A with or without ATP. The results indicate that OXPHOS inhibitors abrogated the expression of c-Myc and ClpP, whereas ATP pretreatment rescued their expression (Figure 2E). Together, these findings suggest that HSP60 regulates c-Myc expression at the mRNA level via β-catenin signaling mediated by mitochondrial ATP production.

### HSP60 and ClpP colocalize and physically interact in mitochondria.

**Figure 3:**
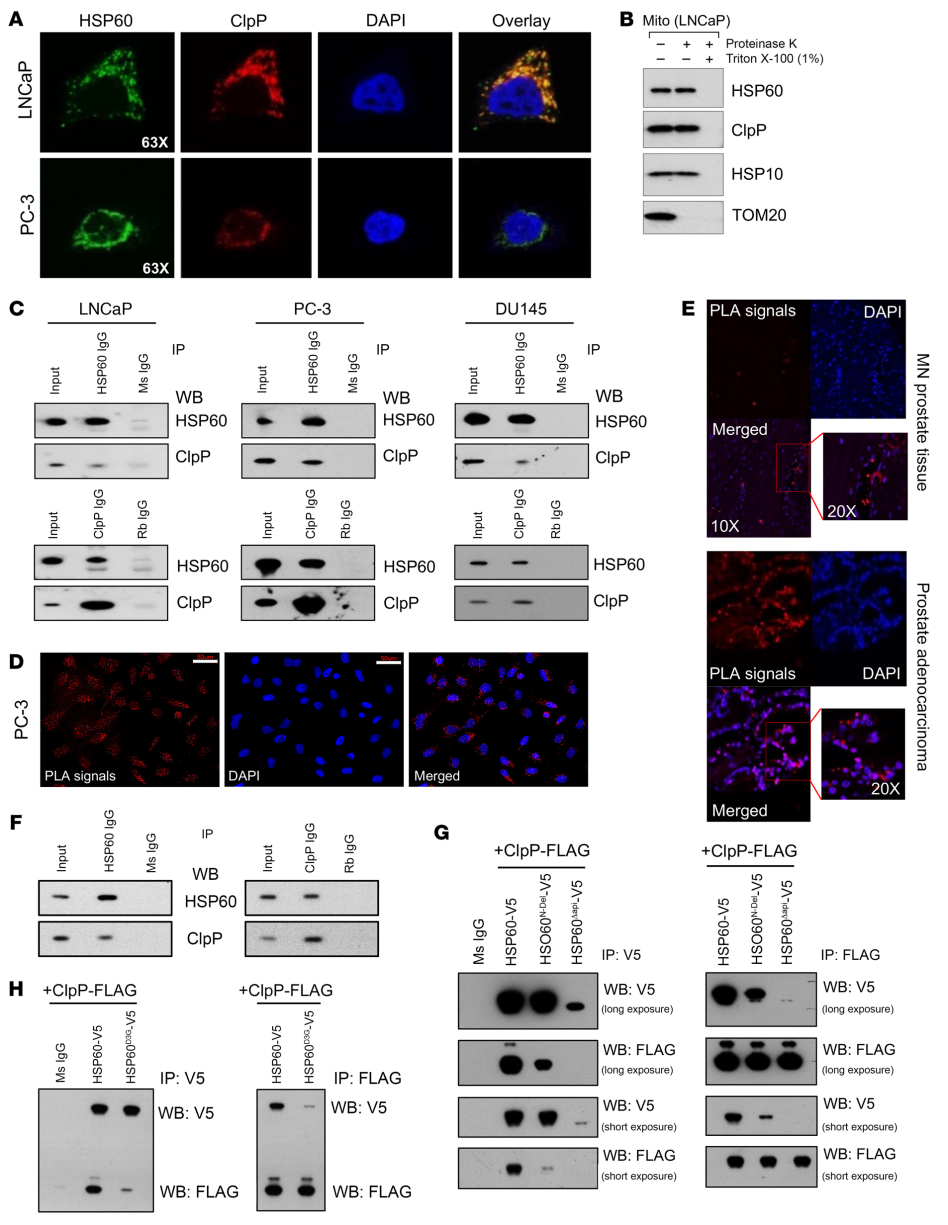
HSP60 and ClpP directly interact in mitochondria. (**A**) Representative immunofluorescence images showing colocalization of HSP60 and ClpP. (**B**) Proteinase K and Triton X-100 digests were performed to determine HSP60, ClpP, and HSP10 colocalization in mitochondria (Mito). (**C**) Co-IPs were performed to determine HSP60 and ClpP interactions in LNCaP, PC-3, and DU145 cells. (**D**) Proximity ligation assay (PLA) between HSP60 and ClpP was performed in PC-3 cells. Scale bars: 50 μm. (**E**) PLA between HSP60 and ClpP was performed in TMA (*n =* 128) constructed from matched normal prostate (MN) and prostate adenocarcinoma tissue. (**F**) Co-IPs were performed to determine HSP60 and ClpP interactions in TKO prostatic tumor tissues. (**G**) Mitochondrial localization signal (HSP60^N-Del^) and apical domain (HSP60^Δapi^) were deleted from the HSP60 construct with a V5 tag and cotransfected with a ClpP construct with a FLAG tag in PC-3 cells. Co-IPs were performed using either anti-V5 antibody or anti-FLAG antibody. Ms IgG, control mouse IgG. (**H**) D3G mutant form of HSP60 (HSP60^D3G^) construct with V5 tag was cotransfected with the ClpP construct with a FLAG tag in PC-3 cells. Co-IPs were performed using either anti-V5 or -FLAG antibody. IP, immunoprecipitation; WB, Western blotting.

Both HSP60/HSP10-mediated protein folding machinery and ClpP-based degradation machinery are required for the maintenance of mitochondrial proteostasis under stress (11–13, 18). Since little is known about the biochemical underpinnings of UPR^mt^ components HSP60 and ClpP as a system, we first evaluated the localization of HSP60 and ClpP in PCa cells. Immunofluorescence analysis demonstrated that HSP60 and ClpP colocalized within mitochondria (Figure 3A), whereas treatment of mitochondria-enriched fractions with proteinase K in the presence of 1% Triton X-100 (to solubilize both outer and inner mitochondrial membranes) suggested colocalization of HSP60 and ClpP in the mitochondrial matrix (Figure 3B). Co-IP studies demonstrated that HSP60 interacted with ClpP in multiple human PCa cell lines (Figure 3C). Proximity ligation assay (PLA) in cultured PCa cells and in patient PCa specimens on a tissue microarray (TMA) revealed in vivo interactions between HSP60 and ClpP (Figure 3, D and E, and Supplemental Figure 3A). In addition, co-IP revealed HSP60-ClpP interactions in murine *Pten^–/–^* *Rb1^–/–^* *p53^–/–^* triple-knockout (TKO) prostate tumors (Figure 3F). HSP60 did not interact with LONP1, another mitochondrial matrix–located protease in PCa cells (Supplemental Figure 3A). As expected, control IgGs did not show PLA signals (Supplemental Figure 3A) and *Hsp60* silencing abrogated or diminished the PLA signals, confirming specificity (Supplemental Figure 3B). Since HSP60 interacts with its cofactor HSP10 (51), we asked whether ClpP and HSP10 would compete for binding with HSP60. We overexpressed ClpP followed by HSP60 IP and observed that ClpP overexpression did not disrupt HSP10’s interaction with HSP60 (Supplemental Figure 4A). Next, we performed double IP in which we first pulled down HSP60-interacting proteins using an anti-HSP60 antibody and then performed either ClpP IP or HSP10 IP in the eluate followed by Western blotting to detect HSP60, HSP10, and ClpP (Supplemental Figure 4B). Our data demonstrated that ClpP and HSP10 did not compete for binding with HSP60, but both were part of the HSP60 complex.

To establish the biochemical basis of the HSP60-ClpP interaction, we created several HSP60 mutants (Supplemental Figure 5) and cotransfected these mutants together with a ClpP-expressing plasmid in PCa cells. As shown in Figure 3G, deletion of either the mitochondrial localization signal (HSP60^N-Del^) and/or apical domain (HSP60^ΔApi^) abolished HSP60’s interaction with ClpP. HSP60 normally oligomerizes to form a tetradecamer structure to exert its biological functions and ClpP proteins also exist in an oligomeric state (27, 29, 52). Consequently, we generated and analyzed the HSP60^D3G^ mutant, which should disrupt HSP60 oligomerization (53). Interestingly, ClpP interaction with HSP60 was significantly diminished upon D3G mutation in HSP60 (Figure 3H). Combined, our findings indicate that HSP60 and ClpP colocalize in the mitochondrial matrix and HSP60 interacts, in its oligomeric form, with ClpP through its apical domain.

### Upregulation of UPR^mt^ components in human PCa.

**Figure 4:**
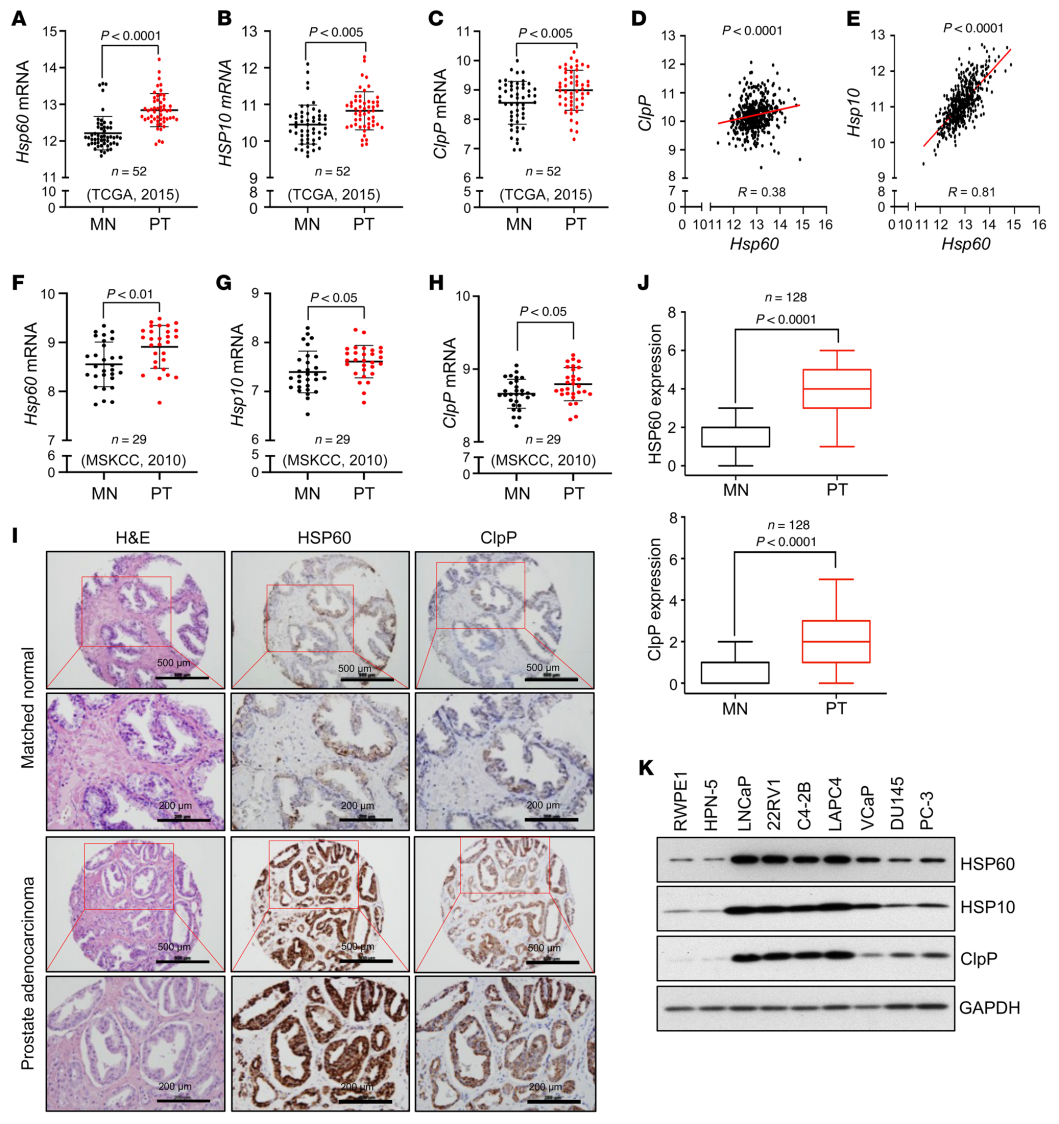
The UPR^mt^ components are upregulated in human PCa. (**A**) *Hsp60* transcript reads in prostate tumors compared to matched normal counterparts from TCGA 2015 data set. (**B**) *Hsp10* transcript reads in prostate tumors (PTs) compared to matched normal (MN) counterparts from TCGA 2015 data set. (**C**) *ClpP* transcript reads in PTs compared to MN counterparts from TCGA 2015 data set. (**D**) Correlative analysis between *Hsp60* and *ClpP* transcript reads from TCGA 2015 data set. (**E**) Correlative analysis between *Hsp60* and *Hsp10* transcript reads from TCGA 2015 data set. (**F**) *Hsp60* transcript reads in PTs compared to MN counterparts from the MSKCC 2010 data set. (**G**) *Hsp10* transcript reads in PTs compared to MN counterparts from the MSKCC 2010 data set. (**H**) *ClpP* transcript reads in PTs compared to MN counterparts from the MSKCC 2010 data set. (**I**) Representative IHC images from PCa TMA stained with H&E and for HSP60 or ClpP. Scale bars: 500 μm (rows 1 and 3) and 200 μm (rows 2 and 4). (**J**) Anti-ClpP and -HSP60 IHC images were scored and quantified. (**K**) Protein expression of HSP60, HSP10, and ClpP in nonmalignant normal prostate cell lines (RWPE1 and HPN-5) and various PCa cell lines. GAPDH serves as a loading control. *P* values were calculated by 2-tailed Student’s *t* test (**A**–**H** and **J**).

To understand the potential biological impact of UPR^mt^ in PCa, we first analyzed the mRNA levels of several key UPR^mt^ components, including 3 mitochondrial chaperonins (*Hsp60*, *mtHsp70*, and *Hsp10*) and 3 mitochondrial proteases (*ClpP*, *LONP1*, and *PARL*), in 52 pairs of PCa and matching normal (MN)/benign prostate tissues in The Cancer Genome Atlas (TCGA) data set. We observed that transcript levels of *Hsp60*, *Hsp10*, and *ClpP* (Figure 4, A–C) as well as *LONP1* (Supplemental Figure 6A) were higher in PCa compared with the MN tissues, whereas *PARL* expression was reduced (Supplemental Figure 6B). Interestingly, there was a strong positive correlation between *Hsp60* and both *Hsp10* and *ClpP* mRNA levels (Figure 4, D and E). A weaker correlation between *Hsp60* and *LONP1* was observed (Supplemental Figure 6C), while no correlation was detected between *Hsp60* and *PARL* (Supplemental Figure 6D). Similarly, analysis of the Taylor et al. Memorial Sloan-Kettering Cancer Center (MSKCC) 2010 data set (see Supplemental Methods) demonstrated that transcript levels of *Hsp60*, *Hsp10*, and *ClpP* (Figure 4, F–H) were higher in PCa compared with the MN tissues, whereas *LONP1* expression did not change (Supplemental Figure 6E) and *PARL* expression was reduced (Supplemental Figure 6F). Transcript levels of *mtHsp70* were higher in PCa compared with the MN tissues in TCGA data set but no significant difference was observed in the MSKCC 2010 data set (Supplemental Figure 6, G and H).

Immunohistochemical (IHC) analysis of HSP60 and ClpP in a TMA containing 128 human PCa and MN tissues revealed significantly higher expression of both proteins in prostate tumors (Figure 4, I and J). Finally, we observed increased expression of HSP60, HSP10, and ClpP in all tested human PCa cell lines compared with nonmalignant prostate epithelial cell lines RWPE-1 and HPN-5 (Figure 4K). Altogether, these data indicate that both the mRNA and protein levels of 3 key components of UPR^mt^ are upregulated in PCa compared with normal or benign prostate epithelium.

### Ablating key UPR^mt^ components inhibits PCa development and growth in vivo.

**Figure 5:**
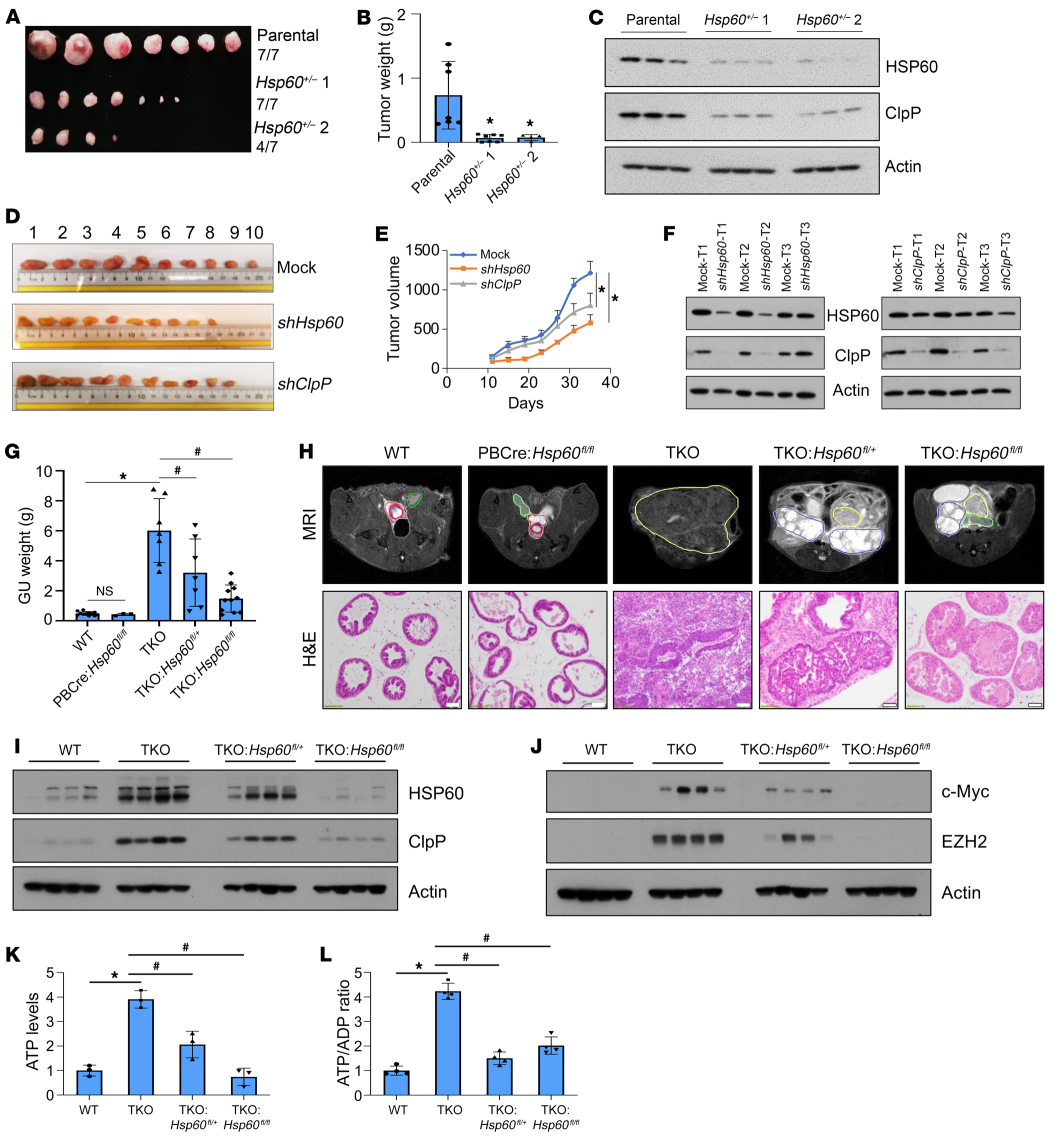
Ablating key UPR^mt^ components inhibits PCa development and growth in vivo. (**A** and **B**) Parental DU145 and *Hsp60^+/–^* DU145 cells were transplanted into SCID mice. Xenograft tumors were harvested and photographed (**A**) and weighed, with the results presented in grams (**B**). (**C**) Whole-cell lysates (WCLs) from parental and *Hsp60^+/–^* DU145 xenografts were analyzed for HSP60 and ClpP by Western blotting. (**D**) *Hsp60*- or *ClpP*-silenced PC-3 cells were transplanted into SCID mice. Xenografts were harvested and photographed. (**E**) *Hsp60*- or *ClpP*-silenced PC-3 cells were transplanted into SCID mice. Tumor size was checked every 4 days and is represented as tumor volume (mm^3^). (**F**) HSP60 and ClpP silencing efficiency in PC-3 cell xenografts was determined using Western blotting. T, tumor. (**G**) WT, *PB*-*Cre4* *Hsp60^fl/fl^*, TKO, TKO *Hsp60^fl/+^*, and TKO *Hsp60^fl/fl^* prostate tissue and tumors were harvested at 16 weeks of age and the whole genitourinary (GU) tract was weighed and is presented in grams. (**H**) WT, *PB*-*Cre4* *Hsp60^fl/fl^*, TKO, TKO *Hsp60^fl/+^*, and TKO *Hsp60^fl/fl^* prostates were imaged by MRI and outlined as indicated (green, normal seminal vesicle [SV]; red, normal prostate; magenta, urethra; yellow, prostate tumor; blue, SV tumor). Mouse prostate tissue and tumors were harvested at 16 weeks and representative H&E-stained images are shown. Scale bar: 100 μm. (**I** and **J**) WT, *PB*-*Cre4* *Hsp60^fl/fl^*, TKO, TKO *Hsp60^fl/+^*, and TKO *Hsp60^fl/fl^* prostate tissue and tumors were harvested at 16 weeks of age and WCLs were prepared and analyzed for HSP60 and ClpP (**I**) and c-Myc and EZH2 (**J**) by Western blotting. (**K**) ATP levels and (**L**) ATP/ADP ratio were analyzed in WT, TKO, TKO *Hsp60^fl/+^*, and TKO *Hsp60^fl/fl^* prostate tissue, represented as fold change compared to WT tissue. Data are mean ± SD (*n* ≥ 3). *P < 0.05 by 1-way ANOVA followed by Dunnett’s multiple-comparison test (**B** and **E**). *P < 0.05; ^#^*P <* 0.05 by 1-way ANOVA followed by Tukey’s multiple-comparison test (**G**, **K**, and **L**). Actin serves as a loading control.

Activation of the UPR^mt^ plays an important role in maintaining mitochondrial functions and cancer cell survival (21). We observed that shRNA-mediated silencing of *Hsp60* and *ClpP* inhibited the clonogenic growth of both androgen-sensitive LNCaP and androgen-independent PC-3 cells (Supplemental Figure 7A, top). Similarly, CRISPR/Cas9-mediated heterozygous loss of *Hsp60* (i.e., *Hsp60^+/–^*) in AR^–^ DU145 cells also reduced its clonogenic capabilities (Supplemental Figure 7A, bottom). We failed to recover homozygous DU145 clones lacking both *Hsp60* alleles (*Hsp60^–/–^*), suggesting that a certain level of HSP60 protein is essential for survival of these cells. Xenograft studies using WT and *Hsp60^+/–^* DU145 cells demonstrated that deletion of one allele of *Hsp60* (i.e., *Hsp60^+/–^* DU145 xenograft) greatly reduced tumor burden, inhibited tumor incidence, and was accompanied by ClpP downregulation (Figure 5, A–C). Likewise, silencing of *Hsp60* or *ClpP* inhibited PC-3 xenograft growth (Figure 5, D–F). Notably, *Hsp60* silencing reduced ClpP expression in vivo but not vice versa (Figure 5F), consistent with the earlier in vitro data (Figure 1, A and B). These findings suggest that HSP60 promotes PCa growth via ClpP expression, although regulation of other mitochondrial activities, including synthesis of macromolecules, import and folding of mitochondrial proteins, and metabolic reprogramming, might also contribute to its PCa-promoting effects (15, 28).

To test the importance of HSP60 in a genetically engineered mouse model of PCa, we bred floxed *Hsp60* alleles (54) into the TKO murine PCa model, which develops AR^–^, highly aggressive neuroendocrine PCa de novo due to concordant deletion of the *Pten*, *Rb1*, and *p53* tumor suppressor genes in prostate epithelial cells (55). TKO animals have an average overall survival of 16 weeks. We euthanized all animals at this age, as they are expected to have readily detectable prostate tumors. Deletion of both *Hsp60* alleles significantly decreased prostate tumor weight and volume in TKO animals (Figure 5, G and H). Remarkably, deletion of even one *Hsp60* allele was sufficient to significantly reduce tumor burden in TKO animals. *Hsp60* deletion also extended the survival of a few TKO tumor-bearing mice from 16 weeks to 28 weeks (data not shown). Of note, *Hsp60* deletion did not apparently affect prostate development and did not generate abnormal prostate phenotypes (Figure 5H). This highlights a reduced requirement for HSP60 in the nonmalignant state of prostate tissue. In contrast, aggressive PCa requires high levels of HSP60 to maintain growth and survival. Indeed, both HSP60 and ClpP were significantly increased in the TKO tumors, which were decreased in TKO *Hsp60^fl/+^* tumors and nearly abolished in TKO *Hsp60^fl/fl^* tumors (Figure 5I).

As observed with DU145 (Figure 5C) and PC-3 (Figure 5F) xenografts, *Hsp60* KO also reduced ClpP levels in TKO tumors (Figure 5I). Similar to the observations in human PCa cells (Supplemental Figure 7, B and C), genetic ablation of *Hsp60* also resulted in decreased expression of oncogenes c-Myc and EZH2 in TKO tumors (Figure 5J). ClpP silencing also inhibited expression of c-Myc and EZH2 in PCa cells (Supplemental Figure 7D). Finally, ATP levels and the ATP/ADP ratio were significantly elevated in TKO tumors, which was abrogated by *Hsp60* deletion (Figure 5, K and L). Collectively, these studies in both human PCa xenograft and genetic murine PCa models reveal a requirement for HSP60 in PCa development and growth.

### An inhibitor of HSP60-ClpP interactions disrupts the UPR^mt^ and induces PCa cell death.

**Figure 6:**
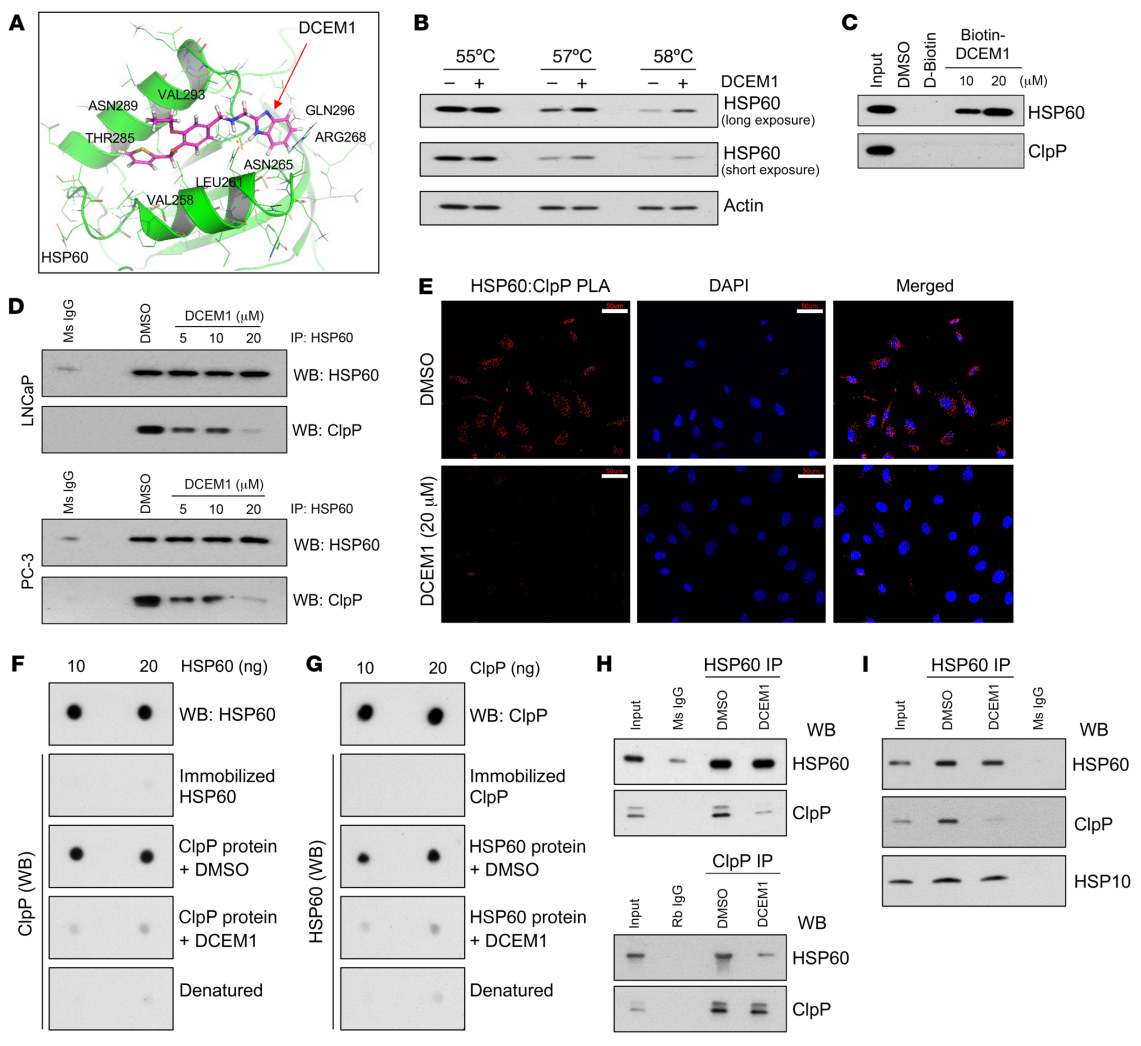
The UPR^mt^ inhibitor DCEM1 disrupts HSP60-ClpP interaction in PCa cells and in vitro. (**A**) Docking of DCEM1 into apical domain of HSP60. (**B**) PC-3 cells were treated with DCEM1 for 1 hour and cellular thermal shift assay (CETSA) was performed for HSP60 protein. Long exposure (LE) and short exposure (SE) of HSP60 are shown. Actin serves as a loading control. (**C**) Western blot analysis of endogenous HSP60 and ClpP protein after biotin-DCEM1 pull-down in PC-3 cell lysates. (**D**) LNCaP and PC-3 cells were treated with DCEM1 for 24 hours and HSP60-ClpP interaction was analyzed by co-IP assay. Ms IgG, mouse control IgG. (**E**) PLA between HSP60 and ClpP was performed in DCEM1-treated PC-3 cells. Scale bars: 50 μm. (**F**) Purified HSP60 protein was dot blotted onto a nitrocellulose membrane and far-Western blotting with ClpP protein with or without DCEM1 (20 μM) was performed. (**G**) Purified ClpP protein was dot blotted onto a nitrocellulose membrane and far-Western blotting with HSP60 protein with or without DCEM1 (20 μM) was performed. (**H**) In vitro co-IP with purified HSP60 and ClpP proteins with or without DCEM1 (20 μM) was performed using either anti-HSP60 or -ClpP antibody. (**I**) LNCaP cells were treated with DCEM1 (20 μM) for 24 hours and HSP60 IP was performed. Samples were analyzed for HSP60, ClpP, and HSP10 by Western blotting.

Having shown that (a) HSP60 chaperonin and ClpP physically interact, and (b) HSP60 is required for prostate tumor development, we aimed to decipher the importance of the HSP60-ClpP interaction in maintaining mitochondrial health and PCa cell survival. We performed in silico screening of a small-molecule library (developed by Enamine Ltd) to identify drugs targeting the apical domain of HSP60, which is critical for its interaction with ClpP (Figure 3G). We identified 9 promising compounds (referred to as A–I) and screened for their cell death–inducing potential in PCa cells (data not shown). We observed that compound A, referred to as DCEM1 (Supplemental Figure 8A), induced robust cell death in PCa cells with little or no effect in nontransformed prostate epithelial RWPE-1 cells (Supplemental Figure 8B and see below). Molecular docking of DCEM1 into the HSP60 protein revealed that DCEM1 binds to the apical domain of HSP60 (Figure 6A). To experimentally substantiate direct binding of DCEM1 to HSP60, we performed 2 independent assays. First, a cellular thermal shift assay (CETSA) demonstrated that treatment of PC-3 cells with DCEM1 (20 μM) for 1 hour led to increased thermal stabilization of HSP60 (Figure 6B). Second, we performed pull-down assays in PC-3 cell lysates using biotin-conjugated DCEM1 (Supplemental Figure 8C), which pulled down endogenous HSP60 but not ClpP (Figure 6C). Together, these 2 assays validated DCEM1 binding to endogenous HSP60 protein.

Having established that DCEM1 binds to HSP60, we asked whether DCEM1 would disrupt HSP60-ClpP interactions in vitro and in PCa cells. Co-IP demonstrated that DCEM1, in a dose-dependent manner, inhibited HSP60-ClpP interactions (Figure 6D). PLA also showed that DCEM1 efficiently inhibited HSP60-ClpP interactions in PCa cells (Figure 6E and Supplemental Figure 8D). To further confirm that DCEM1 disrupts the direct interaction of HSP60 with ClpP, we performed dot-blot far-Western and in vitro IP experiments using purified HSP60 and ClpP proteins in the presence or absence of DCEM1. We observed direct interactions between HSP60 and ClpP proteins, which did not require any other binding partners (Figure 6, F–H). Importantly, we found that DCEM1 effectively disrupted HSP60-ClpP interactions (Figure 6, F–H) but did not affect HSP60-HSP10 interactions (Figure 6I).

**Figure 7:**
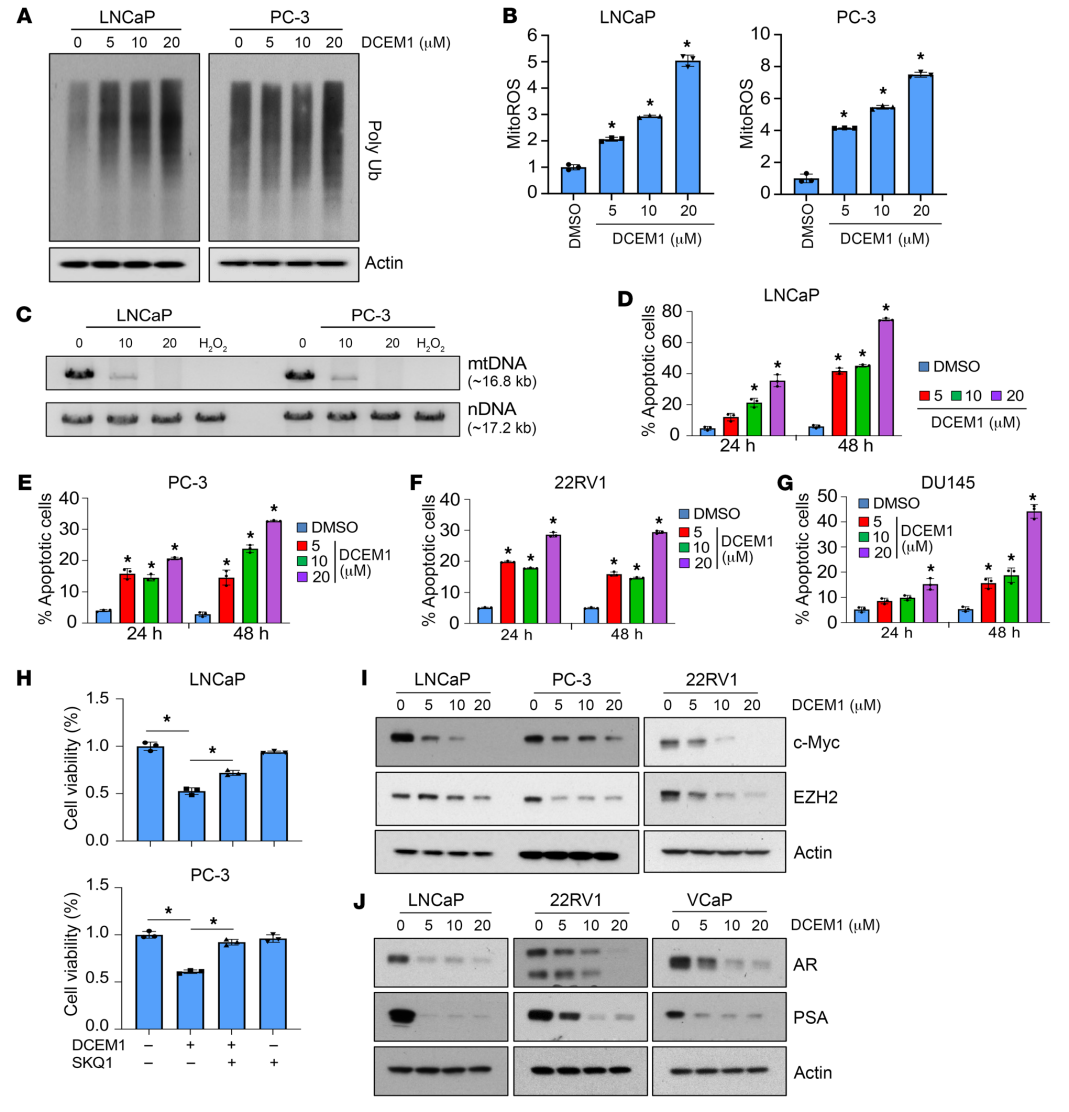
DCEM1 induces proteostatic stress and cell death in PCa cells. (**A**) LNCaP and PC-3 cells were treated with DCEM1 for 24 hours, and poly-Ub protein levels were analyzed by Western blotting. (**B**) LNCaP and PC-3 cells were treated with DCEM1 for 24 hours, and mitoROS levels were analyzed by flow cytometry using mitoSOX dye and are represented as fold change compared to control. (**C**) LNCaP and PC-3 cells were treated with either DCEM1 or H_2_O_2_ (200 μM) for 24 hours, and total DNA was isolated and analyzed for mtDNA damage. (**D**) LNCaP cells were treated with DCEM1 for 24 and 48 hours, and apoptotic cell populations were analyzed using annexin V–FITC/PI. (**E**) PC-3 cells were treated with DCEM1 for 24 and 48 hours, and apoptotic cell populations were analyzed using annexin V–FITC/PI. (**F**) 22RV1 cells were treated with DCEM1 for 24 and 48 hours, and apoptotic cell populations were analyzed using annexin V–FITC/PI. (**G**) DU145 cells were treated with DCEM1 for 24 and 48 hours, and apoptotic cell populations were analyzed using annexin V–FITC/PI. (**H**) LNCaP and PC-3 cells were pretreated with SQM1 (750 nM) followed by DCEM1 (10 μM) treatment, and analyzed for cell viability by MTT assay and are represented as fold change compared to control. (**I**) LNCaP, PC-3, and 22RV1 cells were treated with DCEM1 and analyzed for c-Myc and EZH2 protein expression after 24 hours of treatment. (**J**) LNCaP, 22RV1, and VCaP cells were treated with DCEM1 and analyzed for AR and PSA protein expression after 24 hours of treatment. Data are mean ± SD (*n* ≥ 3). *P < 0.05 compared to respective control by 1-way ANOVA followed by Dunnett’s multiple-comparison test (**B** and **D**–**G**) or 1-way ANOVA followed by Tukey’s multiple-comparison test (**H**). Actin serves as a loading control.

To understand the effect of DCEM1-mediated inhibition of HSP60-ClpP interactions on mitochondrial stress and proteostasis, we analyzed the levels of mitochondrial ROS (mitoROS) and poly-ubiquitinated (poly-Ub) proteins. We observed a dose-dependent upregulation of mitoROS accompanied by increased accumulation of poly-Ub proteins in response to DCEM1 treatment in PCa cells (Figure 7, A and B, and Supplemental Figure 9A), suggesting that inhibition of the HSP60-ClpP interaction interferes with mitochondrial proteostasis. Severe mtDNA damage upon DCEM1 exposure to PCa cells (Figure 7C) further supported the notion that disruption of the HSP60-ClpP interaction by DCEM1 resulted in mitoROS production, mtDNA damage, and subsequent mitochondrial dysfunction. DCEM1 treatment induced caspase activity and apoptotic cell death in PCa cells (Figure 7, D–G, and Supplemental Figure 9B) and inhibited the clonogenic growth of PCa cells (Supplemental Figure 9C). DCEM1 treatment also increased mitoROS and induced accumulation of poly-Ub proteins in TKO PCa cells (established from TKO mouse prostate tumor tissue) as well as caused robust apoptotic cell death (Supplemental Figure 10, A–C). The mitoROS quencher SKQ1 mitigated DCEM1-induced cell death (Figure 7H), suggesting that DCEM1-induced cell death was caused by mitoROS production. *N*-acetyl cysteine pretreatment similarly antagonized DCEM1-induced cell death in PCa cells (Supplemental Figure 11, A and B), confirming the pro-oxidant properties of DCEM1. Interestingly, DCEM1 treatment did not alter the protein levels of UPR^mt^ components, including HSP60, HSP10, ClpP, and LONP1 in most of the PCa cells and normal prostate epithelial cells, and in human embryonic HEK-293 cells (Supplemental Figure 10D and Supplemental Figure 12, A and B), although ClpP and LONP1 expression in PC-3 cells slightly decreased upon DCEM1 treatment (Supplemental Figure 12A). Similar to the c-Myc and EZH2 downregulation when HSP60 or ClpP was silenced or knocked out (Supplemental Figure 7, B–D, and Figure 5J), DCEM1 also robustly downregulated c-Myc and EZH2, and surprisingly, reduced AR and inhibited AR activity (as measured by prostate-specific antigen [PSA] levels) in PCa cells (Figure 7, I and J, Supplemental Figure 10E, and Supplemental Figure 13, A and C). Downregulation of EZH2, AR, and PSA expression in all tested PCa cells by the Myc inhibitor 10058-F4 suggests that HSP60 may regulate these cancer-promoting proteins via c-Myc (Supplemental Figure 13, B and D). These findings indicate that disruption of HSP60-ClpP interactions by DCEM1 inhibits PCa cell survival and tumor growth by deregulating mitochondrial proteostasis, aggravating the generation of mitoROS, and inhibiting key PCa cell survival pathways.

Since the HSP60 chaperonin system is crucial for maintaining mitochondrial homeostasis in normal cells, we developed a method, the mitochondrial chaperonin activity assay (MiCAA), to analyze chaperonin activity in live cells using flow cytometry to evaluate whether DCEM1 modulates mitochondrial chaperonin activity. MiCAA demonstrated that DCEM1 did not modulate mitochondrial chaperonin activity in PCa cells (Supplemental Figure 14A). In contrast, known chaperonin activity and ATPase inhibitors, epolactaene (ETB) and mizoribine (56, 57), respectively, significantly inhibited MiCAA in PCa cells (Supplemental Figure 14B). Genetic approaches using HSP60-specific siRNAs revealed that knocking down HSP60 significantly inhibited mitochondrial chaperonin activity in PCa cells (Supplemental Figure 14C). Collectively, these results indicate that DCEM1 interferes with HSP60-ClpP interactions in PCa cells without altering the chaperonin functions of HSP60.

### Abrogation of HSP60-ClpP interactions by DCEM1 induces metabolic stress in PCa cells.

**Figure 8:**
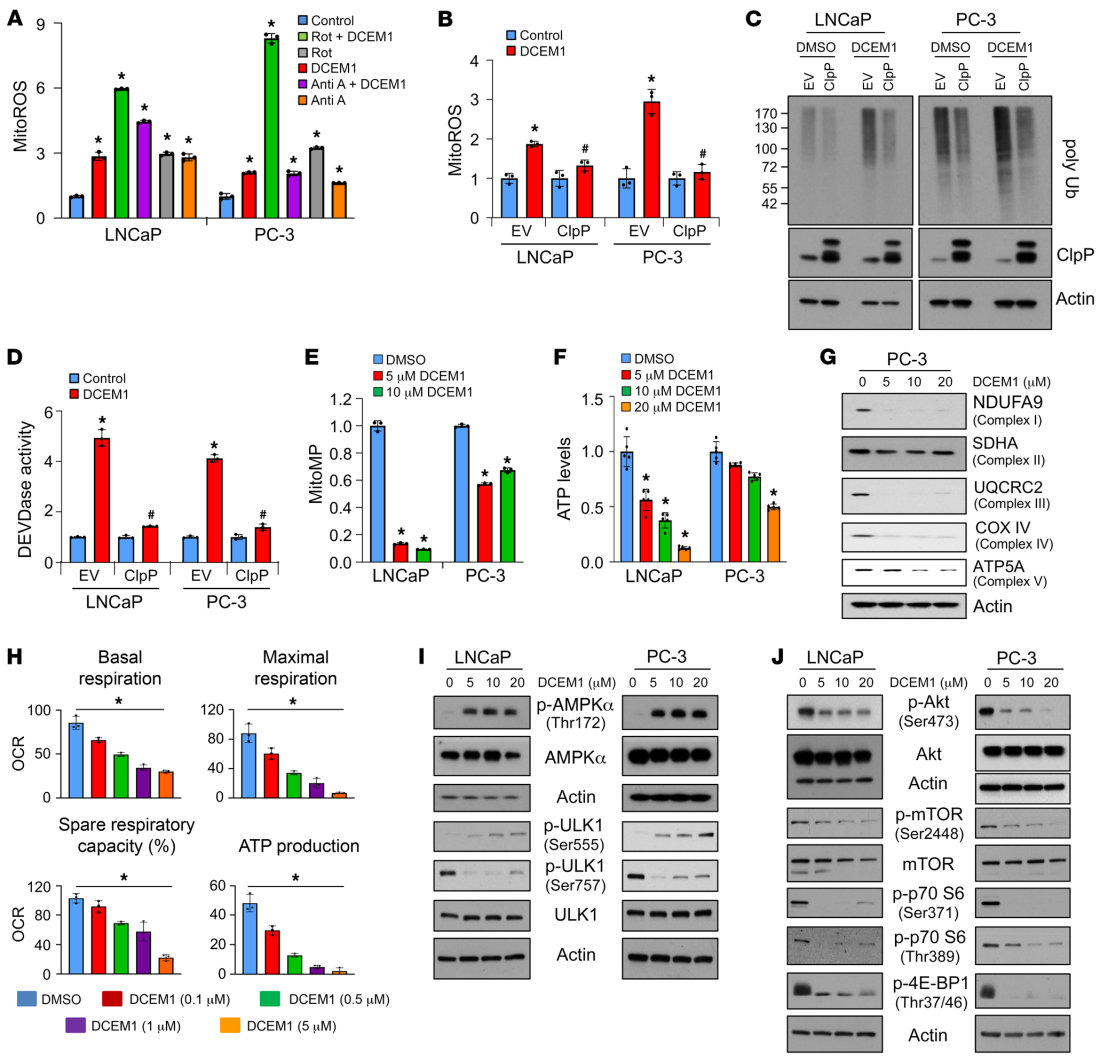
DCEM1 induces metabolic stress in PCa cells. (**A**) LNCaP and PC-3 cells were pretreated with either rotenone (1 μM) or antimycin A (10 μM) followed by DCEM1 (10 μM) treatment, and mitochondrial ROS (mitoROS) were analyzed and are represented as fold change compared to control. (**B**) ClpP protein was overexpressed in LNCaP and PC-3 cells followed by DCEM1 treatment (10 μM), and mitoROS were analyzed and are represented as fold change compared to control. (**C**) ClpP protein was overexpressed in LNCaP and PC-3 cells followed by DCEM1 treatment (10 μM), and the level of poly-Ub protein was analyzed by Western blotting. (**D**) ClpP protein was overexpressed in LNCaP and PC-3 cells followed by DCEM1 treatment (10 μM), and DEVDase activity was analyzed and is represented as fold change compared to control. (**E**) Mitochondrial membrane potential (mitoMP) was analyzed in LNCaP and PC-3 cells treated with DCEM1 and is represented as fold change compared to control. (**F**) ATP level was analyzed in LNCaP and PC-3 cells treated with DCEM1 and is represented as fold change compared to control. (**G**) Protein expression levels of OXPHOS subunits were analyzed in PC-3 cells treated with DCEM1. (**H**) Oxygen consumption rate (OCR) was analyzed in PC-3 cells treated with DCEM1 and is represented as basal and maximal respiration rate, spare respiratory capacity, and ATP production potential. (**I** and **J**) AMPK (**I**) and mTOR (**J**) signaling pathways were analyzed in LNCaP and PC-3 cells treated with DCEM1. Data are mean ± SD (*n* ≥ 3). *P < 0.05 by 1-way ANOVA followed by Dunnett’s multiple-comparison test (**A**, **E**, **F**, and **H**). *P < 0.05, ^#^*P <* 0.05 compared to DCEM1-treated Empty Vector (EV) groups by 1-way ANOVA followed by Tukey’s multiple-comparison test (**B** and **D**). Actin serves as a loading control.

To dissect the underlying mechanism of mitoROS production upon disruption of HSP60-ClpP interactions by DCEM1, we used rotenone to inhibit OXPHOS complex I or antimycin A to inhibit complex III, known sources of mitoROS production (58). We observed that DCEM1-induced mitoROS production was not attenuated by inhibition of complex I or III (Figure 8A). Interestingly, overexpression of ClpP inhibited DCEM1-induced mitoROS production, accumulation of poly-Ub proteins, and DEVDase activity in PCa cells (Figure 8, B–D), suggesting that accumulation of unfolded proteins contributed to mitoROS production. Overexpression of another mitochondrial matrix–localized AAA+ protease, LONP1 (59, 60), also inhibited DCEM1-induced accumulation of poly-Ub proteins, and DEVDase activity in PCa cells (Supplemental Figure 15A). However, overexpression of inner mitochondrial membrane–localized protease PARL (61) did not show any effect on DCEM1-induced accumulation of poly-Ub proteins and DEVDase activity in PCa cells (Supplemental Figure 15B), suggesting that mitochondrial compartment–specific localization of proteases also plays a role in maintaining mitochondrial proteostasis and functions. Given the importance of the UPR^mt^ in maintaining mitochondrial proteostasis and functions, we hypothesized that disruption of HSP60-ClpP interactions promotes metabolic stress in PCa cells. Indeed, DCEM1 treatment robustly depolarized the mitochondrial membrane along with reducing cellular ATP levels and the ATP/ADP ratio, similarly to *Hsp60* or *ClpP* knockdown (Figure 8, E and F, and Supplemental Figure 16, A and B). Interestingly, DCEM1 also abrogated retrograde signaling, as evidenced by downregulation of nuclear DNA–encoded subunits of OXPHOS complexes accompanied by a reduction in oxygen consumption rate (OCR) in PCa cells (Figure 8, G and H).

Another marker of cellular metabolic stress is the AMP-activated protein kinase (AMPK) pathway, which becomes activated under low ATP conditions to restore energy homeostasis (62). Robust increases in p-AMPKα (Thr172) levels were observed in PCa cells upon DCEM1 treatment (Figure 8I). Activation of the AMPK pathway was further confirmed by enhanced phosphorylation at residue Ser555 and decreased phosphorylation at residue Ser757 of UNC-51–like kinase 1 (ULK1) (Figure 8I). DCEM1 treatment also inhibited mTOR signaling in PCa cells, as evidenced by decreased phosphorylation at residue Ser2448 of mTOR, Ser371 and Thr389 of p70 S6, and Thr37/46 of 4E-BP1 (Figure 8J). Together, these results suggest that inhibition of the HSP60-ClpP interaction by DCEM1 causes robust metabolic stress in PCa cells.

### DCEM1 downregulates c-Myc, EZH2, and AR as well as inhibits tumor growth in vivo.

**Figure 9:**
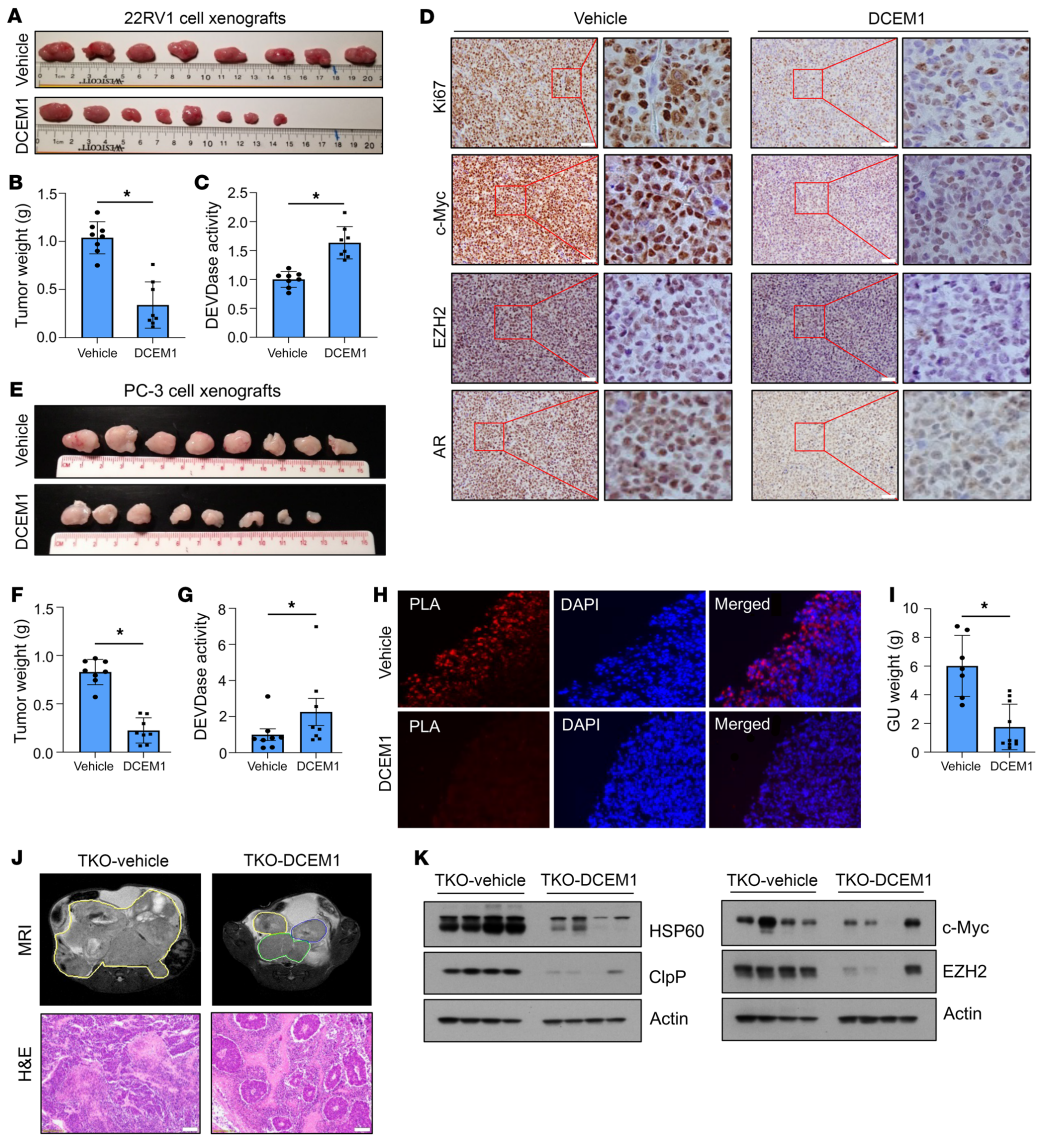
DCEM1 inhibits oncogenic signaling and PCa tumor growth in vivo. (**A** and **B**) 22RV1 cell xenografts were established in each flank of SCID mice and treated with DCEM1 (60 mg/kg body weight, i.p.) twice weekly. Tumors were harvested, photographed (**A**), and weighed (**B**) at 30 days, and results are presented in grams. (**C**) DEVDase activity was analyzed in 22RV1 xenograft tumor tissues following DCEM1 treatment and is represented as fold change compared to vehicle control. (**D**) 22RV1 xenograft tissues were sectioned and expression of Ki67, c-Myc, EZH2, and AR proteins was analyzed by immunohistochemistry. Scale bar: 50 μm. (**E** and **F**) PC-3 cell xenografts were established in each flank of SCID mice and treated with DCEM1 (60 mg/kg body weight, i.p.) twice weekly. Tumors were harvested, photographed (**E**), and weighed (**F**) at 35 days and results are presented in grams. (**G**) DEVDase activity was analyzed in PC-3 xenograft tumor tissue following DCEM1 treatment and is represented as fold change compared to control. (**H**) PC-3 xenograft tumor tissues were fixed and sections were used for in situ PLA to analyze HSP60-ClpP interactions in tumor tissue samples. Original magnification, x40. (**I**–**L**) TKO animals were treated with either vehicle or DCEM1 (60 mg/kg body weight) twice weekly from 10 weeks of age. Animals were sacrificed at 16 weeks of age and the whole genitourinary tract was harvested and weighed (**I**). Animals were imaged by MRI at 16 weeks of age and sacrificed. Prostate tissues and tumors were harvested, and representative H&E-stained images are shown (**J**). Scale bar 100 μm. Whole-tissue lysates from vehicle- or DCEM1-treated (60 mg/kg body weight) TKO tumor tissues were prepared and analyzed for HSP60 and ClpP expression (**K**) and c-Myc and EZH2 expression (**L**) by Western blotting. Data are mean ± SD. *P < 0.05, by 2-tailed Student’s *t* test (**B**, **C**, and **F**–**I**). Actin serves as loading control.

We subsequently determined potential therapeutic effects of DCEM1 on 22RV1 (AR^+^) and PC-3 (AR^–^) xenograft tumor growth in SCID mice (Figure 9, A–H). We observed that DCEM1 at 60 mg/kg body weight effectively inhibited growth of both 22RV1 (Figure 9, A and B) and PC-3 (Figure 9, E and F) xenograft tumors in mice. DCEM1 treatment induced caspase-3/7 (DEVDase) activity in both 22RV1 and PC-3 xenograft tumors (Figure 9, C and G), and significantly downregulated expression of Ki67, c-Myc, EZH2, and AR in 22RV1 xenografts (Figure 9D). Importantly, DCEM1 treatment inhibited HSP60-ClpP interactions in PC-3 xenograft tumors, as supported by PLA (Figure 9H).

To evaluate the efficacy of DCEM1 in spontaneous prostate tumor growth and incidence in an autochthonous PCa model, we treated TKO animals with DCEM1 (60 mg/kg body weight) twice weekly from 10 weeks to 16 weeks of age. As shown in Figure 9I, DCEM1 treatment significantly inhibited TKO tumor growth, as supported by MRI imaging analysis (Figure 9J). DCEM1 downregulated HSP60 and ClpP expression in TKO tumors along with a reduction in c-Myc and EZH2 (Figure 9, K and L), suggesting that DCEM1 phenocopies *Hsp60* deletion in downregulating pro-oncogenic signaling in TKO tumors. Importantly, DCEM1 treatment did not manifest apparent systemic toxicities, as evidenced by body weight measurement (Supplemental Figure 17A), weight and histological evaluation of vital organs (Supplemental Figure 17, B–N), and hematological (Supplemental Figure 18) and clinical chemistry (Supplemental Figure 19) analyses.

## Discussion

**Figure 10:**
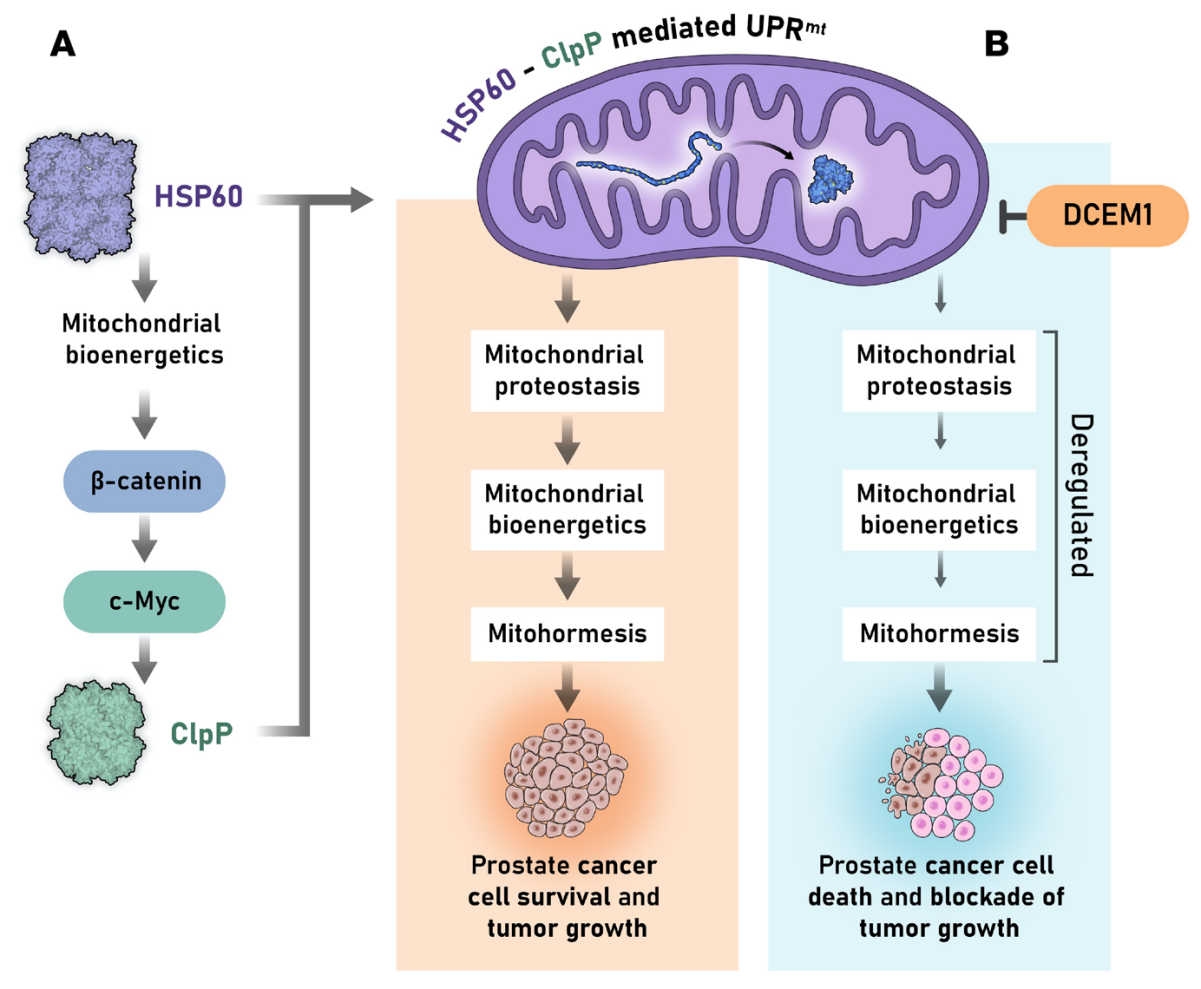
A brief overview of HSP60-ClpP–mediated UPR^mt^ in PCa cell survival and prostate tumor growth. (**A**) HSP60 transcriptionally regulates ClpP expression via c-Myc. Two arms of mitochondrial proteostasis, mitochondrial protein folding (e.g., HSP60) and mitochondrial protease degradation (e.g., ClpP) machineries, interact and cooperate to maintain proteostasis and mitochondrial functions that lead to PCa cell survival and tumor growth. (**B**) Disruption of HSP60-ClpP interactions by UPR^mt^ inhibitor (i.e., DCEM1) deregulates mitochondrial proteostasis, mitochondrial bioenergetics, and mitohormesis, leading to PCa cell death and blockade of prostate tumor growth. Reproduced with permission from Roswell Park Comprehensive Care Center.

Normal cells activate the UPR^mt^ to maintain mitochondrial proteostasis, leading to cellular homeostasis and health (63–66). Cancer cells highjack this unique pathway to promote their long-term survival, contributing to cancer progression and metastasis (3, 67). However, the underlying mechanisms by which mitochondrial proteostasis is exploited by cancer cells such as PCa cells to gain survival advantage are not clearly understood. This study provides evidence suggesting direct cooperation between mitochondrial protein folding and protease degradation machineries to maintain mitochondrial proteostasis in aggressive PCa cells. The discovery that this cooperation requires direct physical interaction between HSP60 and ClpP identifies a vulnerability that could be targeted to disrupt PCa progression. Our findings indicate that disrupting the balance between HSP60-mediated protein folding and ClpP-mediated protein degradation triggers accumulation of unfolded proteins, generates chaos in mitochondrial proteostasis, causes mitochondrial dysfunction and metabolic stress, and ultimately suppresses PCa growth and progression (Figure 10). The HSP60-ClpP interaction is therapeutically targetable, as demonstrated by the discovery of DCEM1, which blocks HSP60 interactions with ClpP, potentially providing an alternative approach to treat aggressive PCa that no longer responds to anti-AR therapy or genotoxic chemotherapy.

The UPR^mt^ consists of 2 protein quality control systems: the protein folding machinery with HSP60 and mtHSP70 as the major chaperone systems, and the proteolysis machinery containing ClpP, LONP1, and many other proteases to degrade unfolded proteins (11, 12, 14, 68, 69). Although both HSP60 and mtHS70 are critical for folding of mitochondrial proteins (12, 14), HSP60 is highly expressed in PCa, suggesting that HSP60-based protein folding machinery plays a critical role in PCa growth. HSP60’s regulation of ClpP suggests that HSP60 and ClpP are concomitantly upregulated in PCa, suggesting hyperactivation of both folding and proteolysis machineries of the UPR^mt^ in PCa growth (Figure 10). One of the most significant findings made in this study is that HSP60, through its apical domain, directly interacts with ClpP, and this interaction requires HSP60 oligomerization. This finding has great implications in designing experimental therapeutics to target the UPR^mt^ in PCa cells. Should we target the expression levels of HSP60 or its chaperonin activity or its interaction with ClpP? Given that HSP60 and ClpP per se are likely important for the survival and functions of not only cancer cells but also normal cells, strategies or drugs that target their expression levels may have unwanted cytotoxicities in the clinic. Likewise, inhibiting HSP60’s chaperonin functions may also have serious deleterious side effects, as observed with phase II clinical trials of an HSP90 inhibitor in metastatic PCa (70). On the other hand, PCa cells selectively, and coordinately, upregulate both HSP60 and ClpP, which interact with each other and work together to maintain mitohormesis (Figure 10). This suggests that the HSP60-ClpP interaction may represent a selective therapeutic vulnerability in PCa cells. Thus, blocking HSP60-ClpP interactions by DCEM1 exhibited impressive therapeutic efficacies in both xenograft and autochthonous TKO models. The availability of DCEM1, a small-molecule inhibitor of the HSP60-ClpP interaction, should allow rapid derivation of a new class of therapeutics to target cancers dependent on a hyperactivated UPR^mt^.

Although it is possible that *Hsp60* silencing and DCEM1 treatment may destabilize ClpP, leading to its degradation, reduced *ClpP* mRNA upon *Hsp60* silencing suggests that degradation of ClpP may not be the main reason for the reduction in ClpP protein. We identify c-Myc as a downstream target of HSP60 to directly regulate ClpP expression and c-Myc overexpression in *Hsp60*-knockdown PCa cells restores ClpP expression. These findings suggest that HSP60 transcriptionally regulates ClpP via c-Myc. Consistently, *Hsp60* deletion, *Hsp60* or *ClpP* knockdown, and DCEM1 inhibited ATP levels in cells and tumors, indicating the requirement for HSP60 in ATP production in mitochondria. ATP is critical for the activation of β-catenin signaling, which upregulates c-Myc expression (47, 49, 50). Thus, ablation of HSP60’s function either genetically or by DCEM1 treatment inhibits ATP production, which abrogates β-catenin–mediated c-Myc expression, leading to ClpP downregulation. These findings support the involvement of mitochondria-to-nucleus (retrograde) signaling in HSP60-mediated PCa growth and progression. Regardless of HSP60 being upstream or downstream of c-Myc, HSP60 has been shown to play an essential role in cellular transformation (71, 72). Our findings have enormous significance in understanding PCa biology because c-Myc represents a key oncogenic driver of prostate tumorigenesis as well as development of aggressive PCa (55, 73–76). In addition, c-Myc regulates EZH2 expression during prostate tumorigenesis via transcriptional and posttranscriptional mechanisms (77). We envision that inhibition of HSP60-ClpP interactions by DCEM1, with subsequent dampening of Myc signaling, will disrupt mitochondrial homeostasis, leading to inhibition of PCa growth and prevention of PCa recurrence.

The majority of mitochondrial proteins are encoded by nuclear DNA and newly synthesized polypeptides are imported to mitochondria for proper folding (78, 79). Cancer cells require increased protein synthesis to meet the demands of increased cellular proliferation, which, conceivably, will cause mitochondrial stress and activation of the UPR^mt^, leading to upregulated synthesis of HSP60 and ClpP (7, 9, 21). Increased levels of these 2 key components of the UPR^mt^ may ultimately impact mitochondrial OXPHOS functions (80), leading to increased mitoROS production, which further enhances the UPR^mt^. How UPR^mt^ activation regulates expression of nuclear DNA–encoded OXPHOS subunits and other mitochondrial functions during tumorigenesis remains to be fully elucidated, but our study indicates that HSP60 is a key regulator of mitochondrial-nuclear crosstalk. This statement is also supported by the observations that mitochondrial proteases such as LONP1 trigger mitochondria-to-nucleus signaling pathways and UPR^mt^ activation (81) and ClpP inhibition diminishes UPR^mt^ signaling (82, 83). The requirement for HSP60 in ATP production further supports the involvement of retrograde signaling during PCa development and progression. Thus, inhibition of ClpP expression and function by *Hsp60* silencing or DCEM1 may abolish mitochondrial-nuclear crosstalk, leading to inhibition of retrograde signaling and tumor growth. Depletion of key PCa-related oncogenic proteins c-Myc and EZH2 upon genetic and pharmacological inhibition of HSP60 and ClpP functions further supports a critical role of the UPR^mt^ in tumor growth and progression. Therefore, there is likely a continued demand for UPR^mt^ activation in attenuating persistent mitochondrial stresses during prostate tumorigenesis and progression.

Although cancer cells prefer aerobic glycolysis for energy, continual production of ATP via the OXPHOS system is still required for rapid cancer cell survival and proliferation (84–88). ClpP along with LONP1 degrades complex I during stress to alleviate mitoROS, thus promoting cell survival (69). *Hsp60* silencing or inhibition of HSP60-ClpP interactions by DCEM1 contributes to mitoROS buildup and mitotoxicity due to inhibition of ClpP function or ClpP deficiency because ClpP is no longer available/functional in alleviating mitoROS production and degrading unfolded proteins in mitochondria, leading to collapse of mitochondrial function and homeostasis. Inhibition of HSP60 renders mitochondria in a fragile and dysfunctional state, leading to enhanced apoptosis and blockage of cellular proliferation. Therefore, OXPHOS collapse upon *Hsp60* and *ClpP* silencing or by DCEM1 treatment causes prominent cell death and may be a major reason why cell proliferation and cell viability are reduced. One of the important characteristics of DCEM1 is that it binds to the apical domain of HSP60, blocking its interaction with ClpP to exert anticancer activity. By contrast, most known inhibitors of HSP60, such as mizoribine, myrtucommulone, and *tert*-butyl ester of ETB block chaperonin or ATPase activity and disrupt protein folding functions of HSP60, and display inefficient anticancer activities (57, 89–92). Our findings show that by abrogating HSP60-ClpP interactions, DCEM1 disrupts mitochondrial proteostasis and effectively causes PCa cell death. Therefore, targeting the HSP60/ClpP axis, which is upregulated in PCa regardless of the AR status, represents a promising therapeutic approach.

## Methods

### Supplemental materials and methods

Additional experimental details as well as a list of antibodies (Supplemental Table 1), shRNA sequences (Supplemental Table 2), and siRNA sources (Supplemental Table 3) are included in the Supplemental Methods.

#### Cell lines.

Human PCa cell lines LNCaP, 22RV1, C42B, PC-3, and DU145; and RWPE-1 (immortalized normal prostate epithelial cells) were purchased and maintained as recommended by ATCC. VCaP and LAPC4 cells were a gift from James Mohler (Roswell Park Comprehensive Cancer Center, Buffalo, New York, USA). DU145 cells heterozygous for the *Hsp60* allele (*Hsp60^+/–^*) were generated using CRISPR/Cas9 by the Genome Engineering and iPSC Center, Washington University (St. Louis, Missouri, USA). Human cell lines acquired from ATCC or collaborators are profiled by short tandem repeat (STR) analysis every 6 months. Early-passage cells are cryopreserved for subsequent use in all experiments to reduce possible genetic drift. Cultures are passaged for no more than 3 months, at which time they are replaced from cryopreserved stocks. Cell lines are screened routinely for mycoplasma contamination using Hoechst staining or a more sensitive PCR assay.

#### Human TMA.

Human PCa adenocarcinoma (*n =* 128) and its MN (*n =* 128) TMA slides were procured from the Pathology Core Resources at Roswell Park Comprehensive Cancer Center and used for either immunostaining or PLA for the key UPR^mt^ proteins HSP60 and ClpP. Primary prostate tumors and MN prostate tissues were collected at Roswell Park Comprehensive Cancer Center by the Pathology Network Shared Resource (PNSR) under an approved IRB protocol (BDR 035513). The patient’s samples were deidentified by PNSR and patient information was not provided to researchers.

#### Generation of conditional KO mice.

Generation of conditional KO mice with a floxed *Hsp60* allele was described previously (54). Generation of *PB*-*Cre4* *Pten^fl/fl^* *Rb1^fl/fl^* *p53^fl/fl^* (TKO) mice was described previously (55). We crossed *Hsp60^fl/fl^* mice with TKO mice to generate *Hsp60*-conditional-KO mice in the TKO background. Genomic DNA was isolated from tail snips using the alkaline lysis method. Briefly, tail snips were incubated in alkaline lysis buffer (25 mM NaOH, 0.2 mM EDTA, pH 12) at 95°C for 45 minutes followed by neutralization buffer (40 mM Tris-HCl, pH 5). Genomic DNA extracts (2 μL) were subjected to PCR for genotyping of different alleles (WT and floxed) using the following primers: *PB-Cre4* transgene Fwd, GCATAACCAGTGAAACAGCATTGCTG and Rev, GGACATGTTCAGGGATCGCCAGGCG; *Pten* floxed allele Fwd, CAAGCACTCTGCGAACTGAG and Rev, AAGTTTTTGAAGGCAAGATGC; *Rb1* floxed allele Fwd, GGAATTCCGGCGTGTGCCATCAATG and Rev, AGCTCTCAAGAGCTCAGACTCATGG; *p53* floxed allele Fwd, GTTAAGGGGTATGAGGGACAAGGTA and Rev, CCATGAGACAGGGTCTTGCTATTGT; *Hsp60* floxed allele Fwd, ACCAAGACCCTGTACTCTTAACC and Rev, AACTTGACCTAGATGTTGTGTGG.

We used a PCR program with an initial denaturation for 3 minutes at 94°C followed by 35 cycles of 94°C denaturation for 15 seconds, 60°C (for *PB*-*Cre4*, *Pten^fl/fl^*, *Rb1^fl/fl^*, and *p53^fl/fl^*) or 54°C (for *Hsp60^fl/fl^*) annealing for 15 seconds, and 72°C primer extension for 30 seconds. A final extension at 72°C was performed for 5 minutes at the completion of the profile. PCR products were resolved in a 2% agarose gel and genotypes were determined as per PCR product size. All animals were sacrificed at 15–16 weeks of age, prostate tumor tissues were flash frozen in liquid nitrogen, and whole-cell lysates were prepared in RIPA buffer for Western blotting. Tissues were also fixed in formalin and processed for H&E staining, as described in Supplemental Methods.

In addition, TKO animals were treated with either vehicle (normal saline/DMSO/Kolliphor HS15 [70:5:20, v/v]; *n =* 7) or DCEM1 (60 mg/kg body weight in vehicle, *n =* 10) twice weekly from 10 weeks of age. All the treated TKO animals were sacrificed at 16 weeks of age, prostate tumor tissues were flash frozen in liquid nitrogen, and whole-cell lysates were prepared in RIPA buffer for Western blotting. Tissues were also fixed in formalin and processed for H&E staining as described in Supplemental Methods.

#### Gene-specific silencing using shRNA lentiviral particles.

Cells were seeded (5 × 10^4^ cells) per well of 6-well plates for 24 hours. Polybrene (8 μg/mL) was added to the media for 1 hour followed by addition of mock shRNA or gene-specific shRNA (*Hsp60* and *ClpP*) lentiviral particles at MOI of 2. After 48 hours of transduction, media were replaced with fresh media containing 1 μg/mL puromycin for selection of transduced cells. Silencing of targeted genes was confirmed using Western blotting (46).

#### ChIP assay.

The association of c-Myc transcription factor with the *ClpP* promoter within the mock and *Hsp60* shRNA–transduced LNCaP and PC-3 cells was detected using a ChIP Assay Kit (Millipore, 17-295) according to the manufacturer’s instructions. To design ChIP PCR primers, the ENCODE database (https://www.encodeproject.org/genes/8192/; Accessed July 6, 2017) was searched for transcription factor binding sites on the *ClpP* promoter using the UCSC genome browser. Ensembl ChIP-seq analysis (https://www.encodeproject.org/search/?type=Experiment&replicates.library.biosample.donor.organism.scientific_name=Homo+sapiens&assay_title=TF+ChIP-seq&status=released&target.label=MYC&biosample_ontology.classification=cell+line) suggested 2 c-Myc–binding DNA regions in the *ClpP* promoter, with the major region being Chr 19: 6361236–6361986, and ChIP PCR primers were designed within this region.

In brief, 1 × 10^6^ cells were fixed in formaldehyde for 15 minutes and chromatin was sheared using a Bioruptor sonicator for 10 minutes in ice with a 30-second on/off cycle (Diagenode). Ten microliters of sonicated samples (of 2 mL total volume) were separated as input. Chromatin was immunoprecipitated with 1.0 μg of anti–c-Myc or normal rabbit IgG (Santa Cruz Biotechnology) antibody at 4°C overnight. Each sample (5 μL) was used as a template for PCR amplification and 20 μL of the 50-μL PCR product was loaded onto agarose gels. The *ClpP* oligonucleotide sequence encompasses the *ClpP* promoter region that includes the c-Myc binding sites for PCR primers 5′-AACCCAGAAGGCAGAGGTTG-3′ and 5′-CACCACGATGGGAATGAGC-3′. PCR mixtures were amplified for 1 cycle at 94°C for 5 minutes followed by 22 cycles at 94°C for 30 seconds, 55°C for 30 seconds, and 72°C for 30 seconds, and then subjected to final elongation at 72°C for 10 minutes. PCR products were resolved in 2% agarose gels and analyzed using ethidium bromide staining (46, 93).

#### Data and materials availability.

All data associated with this study are present in the paper and/or the supplemental material.

#### Statistics.

Statistical analysis was performed using unpaired, 2-tailed Student’s *t* tests for comparison between 2 groups; 1-way ANOVA followed by Dunnett’s multiple-comparison test; or 1-way ANOVA followed by Tukey’s multiple-comparison test for multigroup data sets. All statistical analysis were performed using Prism version 9.3.1 (GraphPad Software). A *P* value of less than 0.05 was considered statistically significant. Significance is denoted as compared with control, unless otherwise indicated.

#### Study approval.

Sections of a PCa TMA constructed from prostate tumors and MNs from PCa patients (*n* = 128) were provided by the PNSR, approved IRB protocol (BDR 035513) at Roswell Park Comprehensive Cancer Center (RPCCC). The patient’s samples were deidentified by PNSR and patient information was not provided to researchers. All mouse experiments were approved by the Institutional Animal Care and Use Committee at RPCCC (IACUC approval no. 1306M).

## Author contributions

DC, RK, and AKC designed experiments. RK, AKC, JRI, J. Woytash, AAG, and NY performed experiments. JAS helped with analysis of MRI imaging. WB helped with IHC analysis of tissue microarrays. KA, J. Wang, and J. Woytash performed statistical and bioinformatic analysis, respectively. ER, DH, DWG, and DGT provided reagents and resources. DC, RK, AKC, and NY analyzed the data. DC and RK wrote the manuscript. DGT, DWG, ER, and DH helped with editing of the manuscript. DC conceived and supervised the study.

## Supplementary Material

Supplemental data

## Version 1 06/02/2022

In-Press Preview

## Version 2 07/01/2022

Electronic publication
