# Supplementary material for: A mitochondrial unfolded protein response inhibitor suppresses prostate cancer growth in mice via HSP60
Source: J Clin Invest. 2022 Jul 1;132(13):e149906. doi: 10.1172/JCI149906 (PMC9246382; doi:10.1172/JCI149906)
Supplement: Supplemental data [file jci-132-149906-s058.pdf]

SUPPLEMENTAL MATERIAL

A mitochondrial unfolded protein response inhibitor suppresses prostate cancer growth in mice via HSP60

Rahul Kumar, Ajay K Chaudhary, Jordan Woytash, Joseph R Inigo, Abhiram A Gokhale, Wiam Bshara, Kristopher Attwood, Jianmin Wang, Joseph Spernyak, Eva Rath, Neelu Yadav, Dirk Haller, David W Goodrich, Dean G Tang and Dhyan Chandra

Supplementary Materials and Methods

Supplementary Figures 1-19

Figure S1. HSP60 regulates ClpP expression in multiple types of cancer cells.
Figure S2. Overexpression of ClpP in HSP60 depleted PCa cells.
Figure S3. HSP60 interacts with ClpP but not with LONP1.
Figure S4. HSP60 interacts with both HSP10 and ClpP, whereas HSP10 and ClpP do not compete for binding with HSP60.
Figure S5. Schematic of various constructs of HSP60 (mutants) and their interactions with ClpP.
Figure S6. Transcript levels of UPR^{mt} components in prostate cancer.
Figure S7. HSP60 or ClpP silencing inhibits clonal growth and reduces c-Myc and EZH2 in PCa cells.
Figure S8. DCEM1 disrupts HSP60-ClpP interactions, and induces a very little or no apoptosis in immortalized untransformed prostate epithelial cells.
Figure S9. DCEM1 induces mitochondrial ROS (mitoROS) production and caspase activation, and inhibits clonogenicity in PCa cells.
Figure S10. DCEM1 inhibits c-Myc and EZH2, and induces mitoROS, polyubiquitination, and apoptosis in TKO cells.
Figure S11. N-acetyl cysteine (NAC) antagonizes DCEM1-induced cell death.
Figure S12. The components of UPR^{mt} were not affected by DCEM1 treatment with exception in PC-3 cells.
Figure S13. DCEM1 and c-Myc inhibitor reduced expression of EZH2 and AR in PCa cells.
Figure S14. Mitochondrial Chaperonin Activity Assay (MiCAA).
Figure S15. LONP1 but not PARL inhibits DCEM1-induced accumulation of poly-Ub proteins and DEVDase activity.
Figure S16. Reduced ATP/ADP ratio upon *Hsp60* or *ClpP* silencing and by DCEM1 treatment in PCa cells.
Figure S17. DCEM1 treatment did not cause organ or systemic toxicities.
Figure S18. DCEM1 treatment of C57BL/6 mice did not change hematological parameters.
Figure S19. DCEM1 treatment of C57BL/6 mice did not change clinical chemistry parameters.

Supplementary Tables

Table S1. List of antibodies used in the study
Table S2. List of shRNAs sequence and their sequences
Table S3. List of siRNAs and their sources

Supplemental Materials and Methods

Whole cell lysates preparation, subcellular fractionation, and Western blotting

Preparation of whole cell lysates (WCL), mitochondrial and cytosolic fractions, and Western blotting were performed as previously described (1-4). The quantification of protein was carried out by micro BCA protein assay kit using BSA as standard (Thermo Fischer, Grand Island, NY). In brief, proteins were separated on sodium dodecyl sulfate-polyacrylamide gel electrophoresis using Criterion Precast Gels (Bio-Rad, Hercules, CA) and transferred on to nitrocellulose membrane (Bio-Rad, Hercules, CA). Nonfat milk (5%) was used to block the membrane for 30 minutes and washed with PBS-T (1XPBS and 0.1% Tween 20) and incubated overnight with indicated primary antibodies and corresponding HRP-conjugated secondary antibodies followed by immunodetection using ECL reagent (BioRad, Herculer, CA). Actin or GAPDH, lactate dehydrogenase B (LDHB) and TOM20 were used as loading and quality controls for WCL, cytosolic and mitochondrial extracts, respectively.

TCGA mRNA expression and gene correlations

The publicly available TCGA IlluminaHiSeq datasets (February 24, 2015) were downloaded in their normalized format from the UCSC Xena Browser and the average transcript reads were calculated for both tumor and matched non tumor specimens. The Read per kilo-base per million mapped reads (RPKM) method was used to quantify the mRNA gene expression from RNA sequencing data by normalizing for total read length and the number of sequencing reads. Spearman's correlation was performed to determine the degree to which two genes are related.

The normalized log2 mRNA expression data and clinical annotations for the MSKCC prostate cancer dataset were downloaded from https://cbio.mskcc.org/cancergenomics/prostate/data/ (5).

Proteinase K digestion of isolated mitochondria

Freshly isolated mitochondria (100 μg) from prostate cancer (PCa) cells were incubated in the homogenization buffer (20 mM HEPES, 10 mM KCl, 1.5 mM $MgCl_2$, 1.0 mM EDTA, 1.0 mM EGTA, 250 mM sucrose) alone or in the presence of proteinase-K (0.1 μg/ml) or proteinase K plus Triton X-100 (1% final concentration v/v). After 10 minutes incubation on ice, 2 μl of 100 μM phenylmethylsulfonyl fluoride (PMSF) was added to terminate proteolysis followed by addition of 6X SDS-loading buffer. Samples were then boiled for 5 minutes and analyzed immediately by Western blotting (6, 7).

Immunohistochemistry (IHC)

Tissues were fixed in 10% normal buffered formalin for 24 h prior to processing. Tissues were processed and embedded in paraffin and then sectioned at 5 microns (5 μm) thickness. Tissue sections were de-paraffinized using xylene and rehydrated with graded alcohol followed by double distilled water (H_2O). Antigen retrieval was performed by boiling with 1X sodium citrate buffer, pH 6.0 (Zymed Laboratories, San Francisco, CA) for 20 minutes. The Dako Autostainer was used to stain slides. Endogenous peroxidase was blocked by immersing slides in 3% H_2O_2 in methanol for 15 minutes. To block non-specific binding, tissues were incubated with 10% normal serum for 30 minutes, followed by avidin/biotin block (Vector Labs Cat#SP-2001). Primary antibodies for mentioned proteins were diluted in 1% BSA solution and incubated for 30 minutes at room temperature (RT), followed by the biotinylated secondary antibodies for 15 minutes. For signal enhancement, ABC reagent (Vector Labs Cat #PK 6100) was applied for 30 minutes. To reveal endogenous peroxidase activity, slides were incubated with 3, 3'-diaminobenzidine tetrahydrochloride (DAB) substrate (Dako Cat #K3467) for 5 minutes and then counterstained

with DAKO Hematoxylin for 20 seconds. Slides were then dehydrated through several baths of graded alcohols and xylenes and then mounted via DAKO Counterstaining solution. All images were taken at three fields (10X, 20X & 40 X magnifications) that showed representative areas of the human prostate tumor tissue microarray (TMA) (3, 4).

Immunoprecipitation

LNCaP, PC-3, and DU145 cells (treated with DCEM1 or vehicle treated); and TKO prostatic tumor tissues were lysed, precleared with mouse or rabbit (depending on the primary antibodies used) IgG-conjugated agarose beads, and incubated with primary antibodies against HSP60, ClpP or IgG (as control antibody), followed by addition of anti-rabbit, or anti-mouse IgG beads. Finally, the beads were pelleted, washed thoroughly, boiled in SDS sample buffer, and analyzed by TrueBlot Western blotting system (eBioscience).

In another experiment, various HSP60-V5 WT and mutant expressing constructs and ClpP-FLAG construct were co-transfected in PC-3 cells. After 48 h of transfection, cells were harvested and lysed, precleared with mouse or rabbit (depending on the primary antibodies used) IgG-conjugated agarose beads, and incubated with primary antibodies against V5 tag, FLAG tag or IgG (as control), followed by addition of anti-rabbit, or anti-mouse IgG beads. Finally, the beads were pelleted, washed thoroughly, boiled in SDS sample buffer, and analyzed by TrueBlot Western blotting system (eBioscience) (3).

Cellular thermal shift assay (CETSA)

CETSA was performed as described previously (8, 9). To determine the binding of DCEM1 with HSP60 within the cells, 2 million PC-3 cells were treated with either DMSO or DCEM1 (20 µM) for 1 hr. Protease inhibitor cocktails (PIC) were directly added to media and cells were harvested by scrapping and washed once with PBS, then resuspended in 500 µl PBS supplemented with 1X PIC and DCEM1 (20 µM). 100 µl cell suspension was distributed into three PCR tubes and incubated at 55^0C, 57^0C and 58^0C for 3 minutes in a PCR machine. Immediately after heating, cell suspensions were further incubated at room temperature for 3 minutes and then snap frozen in liquid nitrogen. Cells were lysed by three freeze-thaw cycles in liquid nitrogen was performed. Cell lysates were collected and centrifuged at 20,000 g for 20 min at 4^0C to remove cell debris and aggregated proteins. Supernatant was collected and 10 µl was heated with SDS sample buffer at 95^0C for 7 minutes and subjected to Western blot analysis for detection of HSP60 and actin.

Biotin-DCEM1 pull down assay

To confirm the direct binding of DCEM1 to endogenous HSP60 protein in PCa cell lysate, biotin conjugated DCEM1 was synthesized (Enamine Ltd). Biotin-DCEM1 pull down of HSP60 was performed according to recent published protocol (8). Whole cell lysates from 60-70 % confluent PC-3 cells were prepared in lysis buffer (25 mM HEPES, ph 7.4, 150 mM NaCl, 1% NP-40, 1 mM EDTA). Protein concentrations were measured using micro BCA protein estimation kit and 100 µg whole cell lysate (total volume 200 µl) was pre-cleared with streptavidin magnetic bead conjugate (Cell Signaling, Cat # 5947) for 1 h at 4^0 C. After pre-clear step, lysates were incubated with 10 and 20 µM Biotin-DCEM1 or 20 µM D-Biotin or DMSO on a tube rotator overnight at 4^0 C. Streptavidin magnetic beads (20 µl) were added to each sample and further incubated on a tube

rotator for 2 h at 4^0 C. Streptavidin magnetic beads were extensively washed 3 times with lysis buffer containing 0.1% BSA followed by 3 washings with lysis buffer without BSA. Finally, beads were incubated at 95^0 C in 2x SDS sample buffer for 7 minutes. Samples were centrifuged and supernatant was subjected to Western blot analysis for HSP60 and ClpP proteins.

Dot Blot Far Western analysis

To detect direct interaction between HSP60 and ClpP, we performed Dot Blot Far Western analysis as previously described (10). Recombinant HSP60 (Abcam, ab113192) and ClpP (Origene, TP300301) proteins were purchased from commercial vendors. 10 and 20 ng of either HSP60 or ClpP protein were dot blotted on a nitrocellulose membrane using a dot blot apparatus (BioRad). The membrane was blocked with 5% BSA and incubated with 5 µg/ml purified ClpP (for HSP60 dot blot) or HSP60 (for ClpP dot blot) protein in 5% BSA with or without 20 µM DCEM1 for 2 h at room temperature. Membranes were washed with TBST and then incubated with HSP60 and ClpP specific antibodies for 2 h at room temperature. After washing with TBST, membranes were incubated with appropriate secondary antibodies for 40 minutes at room temperature. After washing, blots were developed using standard chemiluminescence detection. To show specificity, in one set we denatured protein by heating reaction mixture at 80^0C for 10 minutes.

In vitro co-immunoprecipitation

To detect direct interaction between HSP60 and ClpP, we performed in vitro co-immunoprecipitation using recombinant proteins as described previously with slight modifications (10). Briefly, 200 ng of purified HSP60 protein was incubated with 200 ng of purified ClpP protein for 2 h at 4 °C in immunoprecipitation (IP) buffer (50 mM Tris HCl, 150 mM NaCl, 1% NP-40,

5% glycerol and PIC) with or without 20 μM DCEM1. 1 μg of either HSP60 or ClpP antibody was added to the mix and further incubated at 4^{0} C for overnight. Next day, 25 μl of precleared Protein A/G magnetic beads (ThermoFisher Scientific) was added, and the mixture was incubated for another 2 h at 4 °C. The beads were then washed 5 times in the above-mentioned IP Buffer at room temperature using a DynaL magnetic stand (ThermoFisher Scientific). Respective IgG was used as negative control. Bound proteins were eluted in 1.2x SDS loading buffer heated at 80 °C for 10 minutes.

c-Myc overexpression and silencing

For c-Myc overexpression experiment, PCa cells were seeded into six well plates (2 x 10^5 cells/well) for 24 h. Cells were then transfected with either empty vector or pcDNA3-c-Myc plasmid using lipofectamine 3000 transfection kit as per manufacturer's instructions (Thermo Fisher Scientific, Cat # L3000001). After 48 h, transfected cells were harvested and whole cell lysates were prepared in lysis buffer and subjected to Western blotting for c-Myc and ClpP proteins. pcDNA3-cmyc was a gift from Wafik El-Deiry (Addgene plasmid # 16011; http://n2t.net/addgene:16011; RRID:Addgene_16011) (11). In another set of experiments, PCa cells were transfected with control siRNA or c-Myc siRNA using lipofectamine 3000 transfection kit as per manufacturer's instructions. Cells were harvested after 48 h and whole-cell lysates were prepared in lysis buffer and subjected to Western blotting for c-Myc and ClpP proteins. For lentiviral mediated overexpression of c-Myc, we used pCDH-puro-cMyc plasmid (Addgene plasmid # 46970; http://n2t.net/addgene:46970; RRID:Addgene_46970) to make lentiviral particles followed by infecting stable *Hsp60* silenced LNCaP cells at MOI of 10 and whole cell lysates were prepared after 48 h (12).

Quantification of apoptosis by Annexin V and propidium iodide (PI) staining

PCa cells were harvested at indicated time points and percentage apoptotic cell death was determined using annexin-V-alexafluor 488 / PI kit (Thermo Fisher Scientific, Cat # V13245) according to the manufacturer's instructions. The stained cells were analyzed by flow cytometry (LSRIIA, BD Biosciences) collecting 10,000 events and data were analyzed using Win List 3D software (4).

DEVDase activity measurement

The caspase-3/7 (DEVDase) activity was measured using colorimetric approach method described earlier (4, 13). In a 96 well plate, 5 μL of WCL from each group was incubated with 40 μL of dH_2O and 50 μL of 2 X caspase master mix buffer (0.1 M HEPES, 0.2 M NaCl, 0.2% CHAPS, 40 mM DTT, 2 mM EDTA, 25% glycerol, and fluorescent caspase substrate-caspase-3/7 substrate DEVD-AFC. Plates were incubated for 90 minutes at 37°C in the dark. Fluorescence was read at excitation and emission wavelengths of 400/30 nm and 508/20 nm, respectively using a plate reader (BioTek Microplate Readers, USA). Protein concentration of WCL was determined by microBCA kit (Thermo Fisher Scientific, USA; Cat # 23235) where bovine serum albumin (BSA) was taken as a standard. The fluorescence obtained were normalized by their respective protein concentration. The results were presented as fold change compared to respective controls.

Double immunofluorescence (DIF) and confocal microscopy

Briefly, cells (5000 cells) on coverslips were stained live with DAPI (1 μg/ml) for 15 minutes at 37°C in CO_2 incubator. Cells were fixed with 4% paraformaldehyde containing 5% sucrose for 30 minutes at RT followed by permeabilization with 1% Triton X-100 in PBS for 10 minutes.

Following washing and blocking with 10% goat serum containing 1% Triton X-100 in 1X PBS and washed with 1X PBS twice, respective primary antibodies (HSP60, HSP10 and ClpP) were applied for overnight at 4°C. Alexafluor-488-conjugated and alexafluor-594-conjugated secondary antibodies were added for 2 h. After washing with 1X PBS twice, coverslips were mounted on glass slides using ProLong Gold Antifade Mountant with DAPI as mounting medium. Fluorescent images were acquired using a laser-scanning confocal system (Zeiss LSM710) on an inverted microscope equipped with an oil immersion lens (3, 4, 6).

Cellular reactive oxygen species (ROS), mitochondrial ROS (mitoROS) and mitochondrial membrane potential (mitoMP) using flow cytometry

Flow cytometry analysis was carried out to illustrate cellular and mitoROS, and mitoMP in control and treated groups as described previously (4, 14). The cellular ROS, mitoROS, and mitoMP were estimated in the control and treated groups using DHR-123, MitoSox Red, MitoTracker Green, and MitoTracker Orange probes (Life Technologies, USA), respectively. In brief, control and treated cells were harvested and centrifuged at 1000 rpm for 5 minutes. Cells were then re-suspended in 1 ml of 1X PBS and centrifuged for 5 minutes at 2500 rpm. The pellets were stained with respective probes and incubated for 30 minutes in the dark at 37°C. Cells were centrifuged at 1500 rpm for 5 minutes and pellets were re-suspended in 1X PBS and 10,000 cells were analyzed using flow cytometry (LSR II, BD Biosciences). Data were analyzed by WinList 3D 7.1 software and presented as the fold changes of geometric mean in comparison to untreated control.

ClpP, LONP1, and PARL overexpression

To analyze the effect of mitochondrial proteases ClpP, LONP1, and PARL overexpression on DCEM1-induced proteostatic stress, PCa cells were seeded into six well plates (2 x 10^5 cells/well) for 24 h. Cells were then transfected with either empty vector or ClpP or LONP1 or PARL expressing constructs using lipofectamine 3000 transfection kit as per manufacturer's instructions (Thermo Fisher Scientific, Cat # L3000001). After 48 h, transfected cells were treated with DCEM1 (10 µM) for 24 h, whole cell lysates were prepared in lysis buffer, and subjected to Western blotting for poly-ubiquitinated proteins and DEVDase activity for caspase activity measurements. pcDNA3.2-LonP1-V5 was a gift from David Chan (15). pcDNA3 PARL-FLAG-CT wild type was a gift from Luca Pellegrini (Addgene plasmid # 13639; http://n2t.net/addgene:13639; RRID:Addgene_13639) (16). pReceiver-ClpP-FLAG-M35 construct was purchased from GeneCopoeia (Rockville, MD).

Quantification of ATP levels

Hsp60 and *ClpP* silenced LNCaP and PC-3 cells were seeded in a 96 well white culture plate (5000 cells per well). After 24 h, ATP levels were assayed using luminescent ATP detection assay kit (Abcam, Cat # ab113849) as per manufacturer's instructions. Luminescence signals were read using BioTeK luminometer plate reader. Similarly, ATP levels were estimated in DU145 parental and $HSP60^{+/-}$ cells. In another experiment, LNCaP and PC-3 cells were seeded in a 96 well white culture plate (5000 cells per well). After 24 h cells were treated with DCEM1 (5, 10, and 20 µM), followed by ATP measurement after 24 h as described above. TKO prostatic tumor tissues were homogenized in 1% NP40 buffer and 10 µg protein were diluted in PBS to the volume of 50 µl and ATP levels were estimated as described above.

Measurement of ATP/ADP ratio

In a white 96 well plate, 5 x 10^3 cells were transfected with siRNA against *Hsp60* and *ClpP*. After 48 h the ATP/ADP ratio was measured using the ATP/ADP ratio assay kit (Millipore Sigma) according to the manufacturer's protocol. In another set of experiment, cells were treated with DCEM1 for 24 h and ATP/ADP ratio was measured. TKO prostatic tumor tissues were homogenized in 1% NP40 buffer and 10 µg protein were diluted in assay buffer to the volume of 10 µl followed by estimation of ATP/ADP levels.

Chemical cross-linking and oligomerization assays

Freshly harvested cells were suspended in 45 µl of HIM buffer (10 mm HEPES-KOH, 200 mm mannitol, 70 mm sucrose, 1 mm EGTA, pH 7.5) followed by addition of freshly prepared bismaleimidohexane (BMH) or ethylene glycol bis(succinimidylsuccinate) to a final concentration of 2 mm and incubated at room temperature for 30 minutes. Cells were then mixed with protein sample buffer and subjected to Western blotting (6).

Mitochondrial DNA damage analysis

Mitochondrial DNA damage was analyzed as described previously (17). Briefly, PCa cells were treated with either H_2O_2 (200 µM) or DCEM1 (10 and 20 µM) for 24 h. Total DNA, containing both mtDNA and nuclear DNA, was isolated from cells using the ZR Genomic DNA II Kit (Zymo Research). Total cellular DNA was quantified and 15 ng DNA was used as template to amplify 17.7 kb 5' flanking region of the β-globin gene (nuclear DNA) and 16.2 kb fragment of mitochondrial genome using LongAmp Taq DNA polymerase (NEB). The primer nucleotide sequences were as follows: for β-globin gene, 5'-TTGAGACGCATGAGACGTGCAG-3', and 5'-

GCACTGGCTTAGGAGTTGGACT-3'; and for mitochondrial genome, 5'-TGAGGCCAAATATCATTCTGAGGGGC-3'and5'-TTCATCATGCGGAGATGTTGGATGG -3'. We used the PCR program as follows: an initial denaturation for 3 minutes at 94^0C followed by 30 cycles of 94^0C denaturation for 15 sec, 60^0C annealing for 15 sec and 65^0C primer extension for 15 minutes. A final extension at 65^0C was performed for 10 minutes at the completion of the profile. PCR products were resolved on a 1% agarose gel.

Gene expression analysis by real-time PCR

Total RNA was isolated from *Hsp60* silenced LNCaP and PC-3 cells using the Quick-RNA™ MiniPrep (Zymo Research) kit according to the manufacture's protocol. Total RNA (1 µg) was converted to cDNA using Maxima H minus cDNA synthesis master mix (Thermo Fisher Scientific). Relative mRNA expression was determined on the BioRad CFX Connect Real Time PCR system. The primers used for qRT-PCR are as follows- ClpP: forward-GCGTTGCCAGCCTTGTTATC; reverse- ACTGCATCGTGTCGTAGATGG. Beta-Actin: forward- TCACCCACACTGTGCCCATCTACGA; reverse-CAGCGGAACCGCTCATTGCCAATGG. The real-time PCR reactions were carried out in a total reaction volume of 10 µl containing 20 ng cDNA, 5 µl of 2X iTaq SYBR Green Supermix with ROX (Bio-Rad, Cat# 172-5850), and 300 nM each of forward and reverse primers. A melting curve analysis done at the end of amplification showed the absence of nonspecific amplification or primer dimer formation. The threshold cycle number (Ct) values for each reaction were calculated using the BioRad CFX analysis software. Average threshold cycle number (Ct) values were obtained by amplification of ClpP, and beta-actin. Relative mRNA expression was determined as $2^{-\Delta Ct}$, or fold difference of gene-of-interest and normalized with beta-actin (4).

Seahorse XF96 bioenergetics mitostress assay

Different parameters associated with mitochondrial respiration such as oxygen consumption rate, basal respiration, maximal respiration, ATP production, and spare respiratory capacity in PCa cell lines were assessed by MitoStress assay by using Seahorse XF96 (Seahorse Biosciences, North Billerica, MA, USA) analyzer as described previously (4). *Hsp60* and *ClpP* silenced PCa cells (30000 cells/well) were seeded in seahorse plate and analyzed for above mentioned parameters next day. For DCEM1 treatment related experiments, PC-3 cells (30000 cells/well) were seeded in seahorse plate and treated with indicated doses of DCEM1 next day and analyzed on seahorse analyzer after 16 h treatment.

CLPP promoter reporter assay

CLPP promoter reporter clone and empty pLightSwitch vector were purchased from SwitchGear Genomics, Menlo Park, CA (Cat # S721763). The reporter construct was prepared by cloning 1000 bp of CLPP promoter from the transcription initiation site. Plasmids were transformed in DH5α E. Coli strain and were isolated using a Zymo Plasmid MidiPrep kit (Zymo research, cat # D4200). LNCaP cells were seeded in 96 well plates and transfected with either pLightSwitch empty vector or CLPP promoter reporter clones using a lipofectamine 3000 transfection kit (ThermoFisher Scientific, Waltham, MA). Luciferase activity assay was determined after 48 h using LightSwitch Assay Reagent (SwitchGear Genomics, Menlo Park, CA. Cat # LS010).

Viability assay and clonogenic survival assay

Cell viability was assayed via Trypan blue exclusion. Briefly, 10 µl of cell suspension was added to 10 µl of trypan blue, and 10 µl of the cell suspension-trypan blue solution was added to a

hemocytometer. Live and dead cells were counted, and viability was calculated. For the clonogenic survival assays, stable *Hsp60* and *ClpP* silenced LNCaP and PC-3 PCa cells, as well as DU145 parental and *DU145 Hsp60*$^{+/-}$ cells, were seeded at a density of 500 cells per well in a 6-well tissue culture dish and were cultured for 7 days. Cells were washed with 1X PBS, fixed with chilled 95% ethanol for 10 minutes, and stained with crystal violet solution (0.05% in distilled water) for 30 minutes at room temperature and observed under phase contrast microscope (Carl Zeiss, Thornwood, NY) as previously described (18). In another experiment, PCa cells were seeded at a density of 500 cells per well in a 6- well tissue culture dish and after 24 h treated with DCEM1 (5 μM). After 6 days of treatment, cells were washed with 1X PBS, fixed with chilled 95% ethanol for 10 minutes, and stained with crystal violet solution as described above.

ClpP protease activity in purified mitochondria

Purified mitochondria from PCa cells were isolated as described above. ClpP protease activity in mitochondria was assayed as described earlier (19)**.** In brief, 0.5 mM fluorogenic substrate Suc-LY-AMC (Sigma) was added to 2.5 µg/well mitochondrial lysates in ClpP peptidase assay buffer containing 50 mM Tris-Cl (pH 8.0), KCl (200 mM), DTT (1 mM), 0.1% n-dodecyl β-D-maltoside (DDM), and ATP (2 mM). Fluorescence was measured at an excitation wavelength of 360 nm and emission wavelength of 460 nm at 2 minutes intervals for 1 h. The rate of ClpP activity was normalized with protein concentration of each sample.

Proximity ligation assays (PLA) in PCa cells and tumor tissues

PLA was used to analyze the protein-protein interaction along with the subcellular localization of the observed interaction in PCa cells and TMA using Duolink® In Situ Starter Kit (Sigma, Cat #

DUO92101) as per manufacturer's guidelines. Briefly, 5000 cells (LNCaP, PC-3, DU145) were cultured on a cover slip (12 mm) in 6-well culture plates for 24 h. Cells were fixed with 2% paraformaldehyde (Sigma) in 1X PBS for 30 minutes at RT and then washed twice with 1X PBS. 600 µl of blocking reagents A (2% BSA in 0.1% Triton X 100 in 1X PBS) was added for 1 h, washed once with 1X PBS and then 600 µl of blocking buffer B (1% BSA in 0.1% NP40 in 1X PBS) was added for 1 h, washed twice with 1X PBS and kept overnight at 4^0C. The next day, samples were washed with 1X PBS and primary antibodies specific for HSP60 and ClpP were diluted (1:100) with antibody diluents and added directly on the coverslip for 60 minutes at RT, and then washed twice with Duolink *in situ* wash buffer A (8.8 g NaCl, 1.2 g Tris base, 0.5 ml Tween 20, pH 7.4) with gentle agitation. Negative and positive PLA probes were diluted (1:5) in the antibody diluent. Then, 30 µl of probe mix was added on to the coverslip and subsequently transferred into a humidified chamber for 1 h. The samples were washed with Duolink *in situ* wash buffer A twice for 5 minutes under gentle agitation and the probes were ligated with two circle-forming DNA oligonucleotides by ligation-ligase solution for 30 minutes at 37 °C (4 µl 5X Ligation buffer, 0.5 µl ligase and 15.5 µl dH_2O). The samples were washed in 1X wash buffer A twice at 2 minutes interval under gentle agitation and added fluorescently labelled oligonucleotides buffer (4 µl of amplification red buffer, 0.25 µl polymerase and 15.75 µl dH_2O) to complete the enzymatic ligation via rolling circle amplification under dark conditions for 1 h 40 minutes at 37 °C in the humidified chamber. The samples were washed in 1X wash buffer B (5.84 g NaCl, 4.24 g Tris base, 26.0 g Tris-HCL, pH 7.4) twice for 10 minutes followed by 0.01X wash buffer B for 1 minute by diluting 1X buffer B 1:100 in high purity water under dark conditions and mounted onto a slide in a minimal volume of ProLong Gold antifade reagent with DAPI. Slides were stored at 4°C in the dark. For human PCa TMA slides, the same methods were followed as

mentioned in the IHC protocol up to antigen retrieval and further followed the exact protocol of the *in situ* PLA protocol mentioned above in this section. Subcellular localization of protein-protein interactions was detected using confocal microscope (Zeiss LSM710) (20).

Generation of HSP60 mutants

HSP60 cDNA was PCR amplified from LNCaP cells and cloned at BamHI and XhoI site in pSF-CMV-Puro-COOH vector (Oxford genetics, Oxford, UK. Cat # OG3422) which is a C-terminal V5 epitope tag mammalian expression plasmid. After confirmation of HSP60 expression in PCa cells, several mutants were created in HSP60 gene using a QuickChange II XL site-directed mutagenesis kit (Agilent Technologies, Wilmington, DE. Cat # 200521) as per manufacturer's instructions. The primers were designed using QuikChange Primer Design tool available through Agilent technologies. The mutants are following- A) $HSP60^{N\text{-}Del}$: Mitochondrial localization signal (MLS) was deleted from N terminal of HSP60 by deleting nucleotide from +4 to +75 from HSP60 gene. B) $HSP60^{\Delta api}$: Apical domain was deleted from HSP60 gene by deleting nucleotide from +1090 to +1188. C) $HSP60^{D3G}$: D3G mutant was created by replacing nucleotide A at +86 position with G to replace Aspartic acid (D) with Glycine (G). All constructs were confirmed by sanger sequencing at Roswell Park Comprehensive Cancer Center genomics core facility.

Magnetic resonance imaging (MRI) imaging

Magnetic resonance imaging was carried out on a 4.7 Tesla preclinical scanner using the Paravision 3.0.2 imaging platform (Bruker Biospin, Billerica MA) and a custom-built, 35 mm ID quadrature radiofrequency coil (m2m Imaging, Cleveland OH). Following scout scans, a T2-weighted, fast spin-echo scan was acquired with chemical shift, fat saturation (CHESS) in the axial

orientation to visualize the prostate and seminal vesicles. Pertinent imaging parameters include: effective echo time = 40 ms, repetition time = 2800 ms, in-plane resolution = 125 micron, slices = 25, slice thickness = 1 mm. An additional T2-weighted scan without fat saturation was acquired with identical geometry to assist in delineation of anatomy. Regions of interest (ROIs) were created in Analyze 7.5 (AnalyzeDirect, Overland Park KS) for the prostate, seminal vesicles, and urethra, as well as tumors within both the prostate and seminal vesicles, with the aid of a mouse atlas (21) and previously reported MR images of mouse prostate (22). Volumes for each ROI were calculated in Analyze and are reported herein.

Mitochondrial Chaperonin Activity Assay (MiCAA)

We analyzed mitochondrial chaperonin activity assay as reported earlier with modifications (23). To analyze the endogenous mitochondrial chaperonin (protein folding) activity, we co-transfected PCa cells with pmCherry-N1 (Clontech Lab. Cat # 632523) and EYFP-Mito [EYFP-Mito-7 was a gift from Michael Davidson (Addgene plasmid # 56596 ; http://n2t.net/addgene:56596 ; RRID:Addgene_56596)] (24). In brief, transfected cells were treated with either DCEM1 or mizoribine or tert-butyl ester of epolactaene (ETB) after 6 h post transfection. After 24 h, cells were harvested and analyzed by flow cytometry using PE Texas Red and FITC channels. Average fluorescent intensity for both channels were determined by FACS Diva software and YFP signal intensity was normalized with RFP signal intensity and plotted as fold change compared to control. For *Hsp60 siRNA* MiCAA experiment, 100 nM Hsp60 specific predesigned *DsiRNAs* (IDT, Design ID hs.Ri.HSPD1.13.1 and hs.Ri.HSPD1.13.3) were transfected into PCa cells and after 6 h, pmCherry-N1 and EYFP-Mito plasmids were transfected and analyzed by flow cytometry as mentioned above.

Structure-based virtual drug screening for HSP60 inhibitors

Virtual screening experiments to identify HSP60 inhibitors were performed with Schrödinger software (Small molecule drug discovery suite, v2018-2). The X-ray crystal structure of the HSP60-HSP10 human mitochondrial chaperonin complex (PDB 4PJ1) was used as the model for docking experiments. Proteins were prepared for docking studies using the Protein Preparation Wizard (25). Specifically, we elected to assign bond orders, add hydrogen atoms, create bonds between sulfur atoms within 3.2 Å, and remove crystallographic water molecules > 5 Å from heteroatom groups. The protein hydrogen-bonding network was optimized using the automated optimization at pH 7.0 and the overall structure was minimized to the lowest energy state using the OPLS3 force field. The virtual chemical library was developed from the Enamine 3D Diversity Set (based on 3D shape diversity) and Enamine pharmacological diversity set (based on compounds with known pharmacological effects). Ligands were prepared using LigPrep (Schrödinger) and settings were as follows: OPLS3 force field, ionization states at pH 7.0 +/- 2.0, ligands were desalted if necessary, any possible tautomers were generated, specified chiralities were retained, and the lowest energy ring conformation was kept. Using the GLIDE program as described (26), we set up a virtual screening platform to identify HSP60 inhibitors. All docking settings were set to default. To progressively filter out hits from non-hits, we performed three stages of GLIDE docking, each stage with an increased stringency: HTVS (High-Throughput Virtual Screening), SP (Standard Precision), and XP (Extra Precision). Top scoring molecules were sorted based on glide score (-kcal/mol) and the top 25% from HTVS were processed to SP docking and the top 15% from SP were processed to XP docking. The top 50-100 scored molecules after XP docking were visually inspected for differences in molecular interactions, erroneous poses, or similar binding modes (pharmacophores). The top molecules were subsequently clustered

based on structural similarity to identify around 10-20 structurally distinct clusters. A single representative molecule from each cluster was selected for purchase.

Xenograft studies

Roswell Park Comprehensive Cancer Center (RPCCC) ethical committee IACUC (Institute Animal Care and Use Committee) approved all mouse protocols used in this study (IACUC approval # 1306M). Mice were housed in an animal facility maintained on a 12 h light/dark cycle, at a continual temperature (22±2°C) and relative humidity (55±15%), tap water and food were available ad libitum. C.B-Igh-1b/IcrTac-Prkdcscid male mice (4–6 weeks old) were purchased from an in-house colony maintained at Roswell Park Comprehensive Cancer Center (NY, USA). To assess the effect of HSP60 depletion on PCa tumor growth in vivo, SCID male mice were inoculated with *DU145 WT*, *DU145* $Hsp60^{+/-}$ 1 and *DU145* $Hsp60^{+/-}$ 2 (5×10^5 Cells/Flank) suspended in Matrigel (1:1 ratio, v/v) by subcutaneous injection on both flanks. In another experiment, to assess the effect of *Hsp60* and *ClpP* silencing on PCa tumor growth in vivo, SCID male mice were inoculated with stable PC-3 cells expressing either *shMock*, *shHsp60*, or *shClpP* (1×10^6 Cells/Flank) suspended in matrigel by subcutaneous injection on both flanks. Tumor growth was assessed by digital caliper measurements (VWR® Digital Caliper) twice weekly.

To analyze the effect of DCEM1 on PCa xenograft tumor growth, PC-3 and 22RV1 cells (1×10^6 Cells/Flank) mixed with Matrigel (1:1) and injected subcutaneously in the right and left flanks of each mouse. When xenograft tumors reached 5 mm in diameter (~18 days post injection), mice were randomly divided into 2 groups of 4 mice. First group received 100 µl vehicle (75% normal saline:20% Kolliphor HS15:5% DMSO). Second group received 100 µl vehicle and DCEM1 (60 mg/kg body weight) twice weekly. All treatments were administered for 3 weeks. Mice were

euthanized, tumors were excised and flash frozen in liquid nitrogen. The tissue lysates were used for analyzing caspase-3 activity. Tumor tissues were also fixed in formalin and processed for IHC staining for indicated antibodies.

Toxicity assessment of DCEM1 in C57BL/6 animals

For general assessment of DCEM1 toxicity in C57BL/6 male mice, 10 weeks old animals (n=16) were bought from Jackson Laboratories, Bar Harbor. After acclimatization in Roswell Park Comprehensive Cancer Center animal house, animals were randomly divided into two groups (n=8) and treated with 100 µl Vehicle (75% Normal Saline: 20% Kolliphor HS15: 5% DMSO) or DCEM1 (60 mg/kg body weight) in vehicle twice weekly via i.p. injections. Body weights of experimental animals were measured once per week. All treatments were administered for 6 weeks. At the end of experiment, animals were euthanized and main organs (Prostate, Liver, Kidney, Heart, Pancreas, Lung and Spleen) were collected, weighed and processed for H&E staining. Blood samples were also collected from all the animals for hematological and clinical chemistry related parameters (in serum). Hematological and Clinical Chemistry analyses were performed by the Laboratory Animal Shared Resources of Roswell Park Comprehensive Cancer Center using IDEXX Procyte A5088 Hematology analyzer for hematology samples, and IDEXX Catalyst DS Chemistry Analyzer for chemistry samples in freshly isolated blood and serum samples from experimental animals.

References

1. Chandra D, Choy G, and Tang DG. Cytosolic accumulation of HSP60 during apoptosis with or without apparent mitochondrial release: evidence that its pro-apoptotic or pro-survival functions involve differential interactions with caspase-3. *J Biol Chem.* 2007;282(43):31289-301.
2. Chandra D, Liu JW, and Tang DG. Early mitochondrial activation and cytochrome c up-regulation during apoptosis. *J Biol Chem.* 2002;277(52):50842-54.
3. Gogada R, Yadav N, Liu J, Tang S, Zhang D, Schneider A, et al. Bim, a proapoptotic protein, up-regulated via transcription factor E2F1-dependent mechanism, functions as a prosurvival molecule in cancer. *J Biol Chem.* 2013;288(1):368-81.
4. Kumar R, Bhat TA, Walsh EM, Chaudhary AK, O'Malley J, Rhim JS, et al. Cytochrome c Deficiency Confers Apoptosome and Mitochondrial Dysfunction in African-American Men with Prostate Cancer. *Cancer Res.* 2019;79(7):1353-68.
5. Taylor BS, Schultz N, Hieronymus H, Gopalan A, Xiao Y, Carver BS, et al. Integrative genomic profiling of human prostate cancer. *Cancer Cell.* 2010;18(1):11-22.
6. Gogada R, Prabhu V, Amadori M, Scott R, Hashmi S, and Chandra D. Resveratrol induces p53-independent, X-linked inhibitor of apoptosis protein (XIAP)-mediated Bax protein oligomerization on mitochondria to initiate cytochrome c release and caspase activation. *J Biol Chem.* 2011;286(33):28749-60.
7. Chandra D, Choy G, Daniel PT, and Tang DG. Bax-dependent regulation of Bak by voltage-dependent anion channel 2. *J Biol Chem.* 2005;280(19):19051-61.
8. Han H, Jain AD, Truica MI, Izquierdo-Ferrer J, Anker JF, Lysy B, et al. Small-Molecule MYC Inhibitors Suppress Tumor Growth and Enhance Immunotherapy. *Cancer Cell.* 2019;36(5):483-97 e15.
9. Jafari R, Almqvist H, Axelsson H, Ignatushchenko M, Lundback T, Nordlund P, et al. The cellular thermal shift assay for evaluating drug target interactions in cells. *Nat Protoc.* 2014;9(9):2100-22.
10. Adhikari H, Kattan WE, Kumar S, Zhou P, Hancock JF, and Counter CM. Oncogenic KRAS is dependent upon an EFR3A-PI4KA signaling axis for potent tumorigenic activity. *Nat Commun.* 2021;12(1):5248.
11. Ricci MS, Jin Z, Dews M, Yu D, Thomas-Tikhonenko A, Dicker DT, et al. Direct repression of FLIP expression by c-myc is a major determinant of TRAIL sensitivity. *Mol Cell Biol.* 2004;24(19):8541-55.
12. Cheng Z, Gong Y, Ma Y, Lu K, Lu X, Pierce LA, et al. Inhibition of BET bromodomain targets genetically diverse glioblastoma. *Clin Cancer Res.* 2013;19(7):1748-59.
13. Yadav N, Gogada R, O'Malley J, Gundampati RK, Jayanthi S, Hashmi S, et al. Molecular insights on cytochrome c and nucleotide regulation of apoptosome function and its implication in cancer. *Biochim Biophys Acta Mol Cell Res.* 2020;1867(1):118573.
14. Yadav N, Kumar S, Marlowe T, Chaudhary AK, Kumar R, Wang J, et al. Oxidative phosphorylation-dependent regulation of cancer cell apoptosis in response to anticancer agents. *Cell death & disease.* 2015;6:e1969.
15. Shin CS, Meng S, Garbis SD, Moradian A, Taylor RW, Sweredoski MJ, et al. LONP1 and mtHSP70 cooperate to promote mitochondrial protein folding. *Nat Commun.* 2021;12(1):265.

16. Sik A, Passer BJ, Koonin EV, and Pellegrini L. Self-regulated cleavage of the mitochondrial intramembrane-cleaving protease PARL yields Pbeta, a nuclear-targeted peptide. *J Biol Chem.* 2004;279(15):15323-9.
17. Yakes FM, and Van Houten B. Mitochondrial DNA damage is more extensive and persists longer than nuclear DNA damage in human cells following oxidative stress. *Proc Natl Acad Sci U S A.* 1997;94(2):514-9.
18. Franken NA, Rodermond HM, Stap J, Haveman J, and van Bree C. Clonogenic assay of cells in vitro. *Nat Protoc.* 2006;1(5):2315-9.
19. Cole A, Wang Z, Coyaud E, Voisin V, Gronda M, Jitkova Y, et al. Inhibition of the Mitochondrial Protease ClpP as a Therapeutic Strategy for Human Acute Myeloid Leukemia. *Cancer Cell.* 2015;27(6):864-76.
20. Mukhopadhyay UK, Oturkar CC, Adams C, Wickramasekera N, Bansal S, Medisetty R, et al. TP53 Status as a Determinant of Pro- vs Anti-Tumorigenic Effects of Estrogen Receptor-Beta in Breast Cancer. *J Natl Cancer Inst.* 2019;111(11):1202-15.
21. Iwaki T YH, Hayakawa T. *A Color Atlas of Sectional Anatomy of the Mouse.* Braintree MA: Braintree Scientific Inc; 2001.
22. Ravoori M, Duggal J, Gagea M, Han L, Singh S, Liu P, et al. Visualizing the prostate gland by MR imaging in young and old mice. *PLoS One.* 2013;8(3):e55746.
23. Lu Z, Chen Y, Aponte AM, Battaglia V, Gucek M, and Sack MN. Prolonged fasting identifies heat shock protein 10 as a Sirtuin 3 substrate: elucidating a new mechanism linking mitochondrial protein acetylation to fatty acid oxidation enzyme folding and function. *J Biol Chem.* 2015;290(4):2466-76.
24. Rizzo MA, Davidson MW, and Piston DW. Fluorescent protein tracking and detection: fluorescent protein structure and color variants. *Cold Spring Harb Protoc.* 2009;2009(12):pdb top63.
25. Sastry GM, Adzhigirey M, Day T, Annabhimoju R, and Sherman W. Protein and ligand preparation: parameters, protocols, and influence on virtual screening enrichments. *J Comput Aided Mol Des.* 2013;27(3):221-34.
26. Friesner RA, Murphy RB, Repasky MP, Frye LL, Greenwood JR, Halgren TA, et al. Extra precision glide: docking and scoring incorporating a model of hydrophobic enclosure for protein-ligand complexes. *J Med Chem.* 2006;49(21):6177-96.

Supplementary Figures and Tables

A mitochondrial unfolded protein response inhibitor suppresses prostate cancer growth in mice via HSP60

Rahul Kumar, Ajay K Chaudhary, Jordan Woytash, Joseph R Inigo, Abhiram A Gokhale, Wiam Bshara, Kristopher Attwood, Jianmin Wang, Joseph Spernyak, Eva Rath, Neelu Yadav, Dirk Haller, David W Goodrich, Dean G Tang and Dhyan Chandra

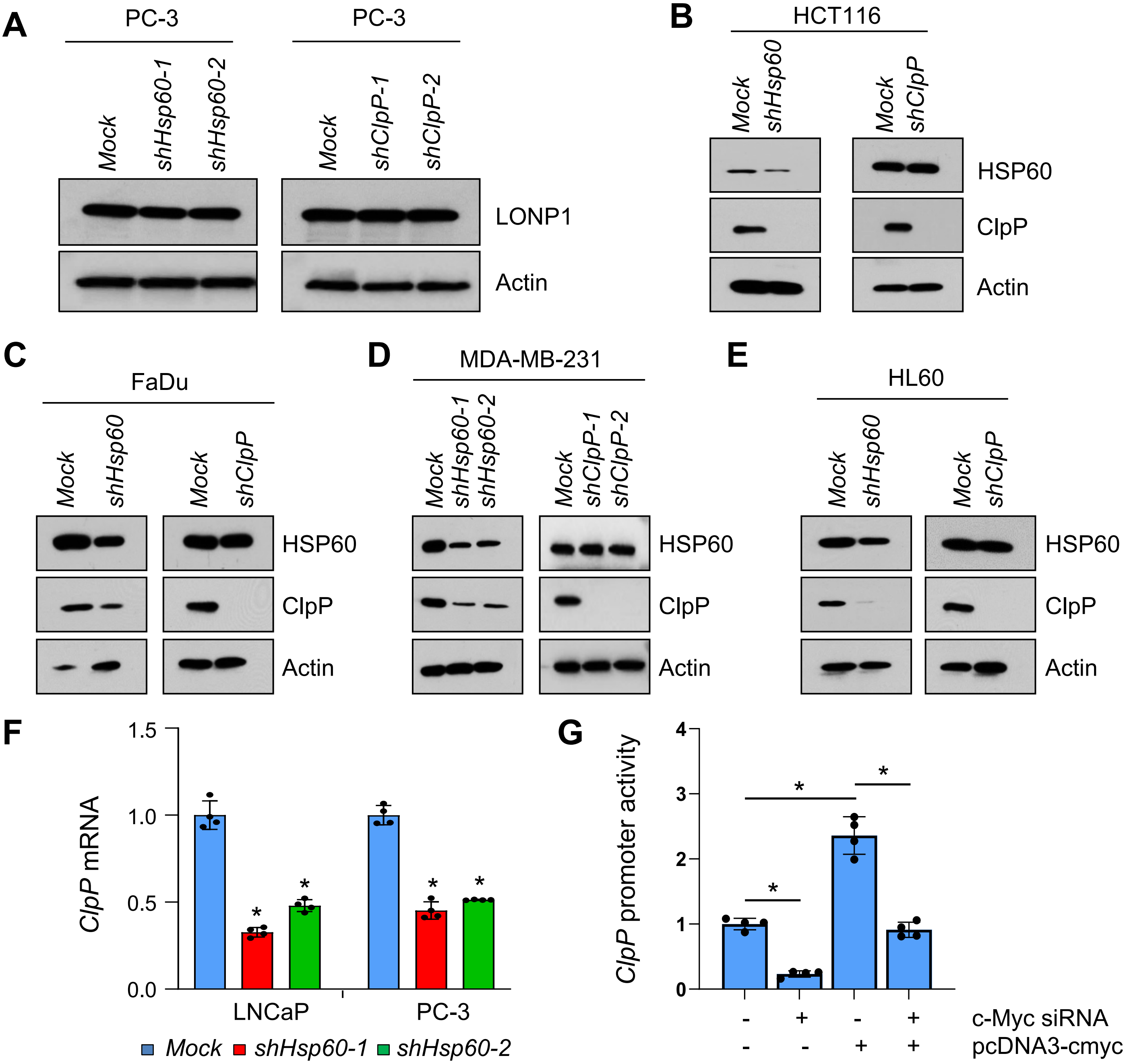

Figure S1: HSP60 regulates ClpP expression in multiple types of cancer cells.
(A) Expression of LONP1 was analyzed by Western blotting upon *Hsp60* and *ClpP* silencing in PC-3 cells. Actin serves as a loading control. **(B-E)** *Hsp60* silencing reduced ClpP expression but not vice versa in HCT116 (**B**, human colorectal carcinoma), FaDu (**C**, human pharynx squamous cell carcinoma), MDA-MB-231 (**D**, human triple negative breast cancer cells), HL60 (**E**, human acute promyelocytic leukemia). Whole cell lysates were used for Western blotting for indicated proteins. Actin serves as a loading control. **(F)** *ClpP* mRNA levels were analyzed in *Hsp60* silenced LNCaP and PC-3 PCa cells and data presented as fold change compared to mock control groups. **(G)** LNCaP cells were transfected with either c-Myc siRNA or pcDNA3-cmyc or both along with pLightSwitch-CLPP PROM construct. After 48 h, cells were analyzed for luciferase activity and data presented as fold changes compared to control group. Results in F and G are mean ± SD (n=4). *p<0.05 by 1-way ANOVA followed by Dunnett's multiple-comparison test (**F**). *p<0.05 by 1-way ANOVA followed by Tukey's multiple-comparison test (**G**).

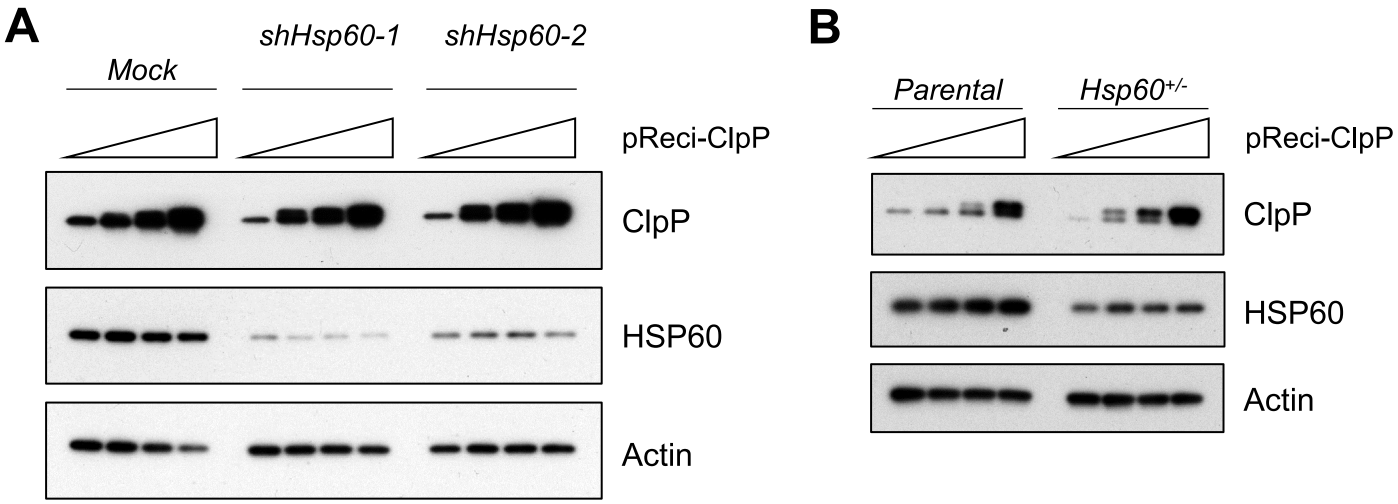

Figure S2: Overexpression of ClpP in HSP60 depleted PCa cells.
(A) *Hsp60* silenced LNCaP cells were infected with ClpP overexpressing lentivirus and ClpP levels were analyzed after 48 h. Actin serves as a loading control. **(B)** *Hsp60* heterozygous DU145 cells (DU145$^{+/-}$) were infected with ClpP overexpressing lentivirus and ClpP levels were analyzed after 48 h. Actin serves as a loading control.

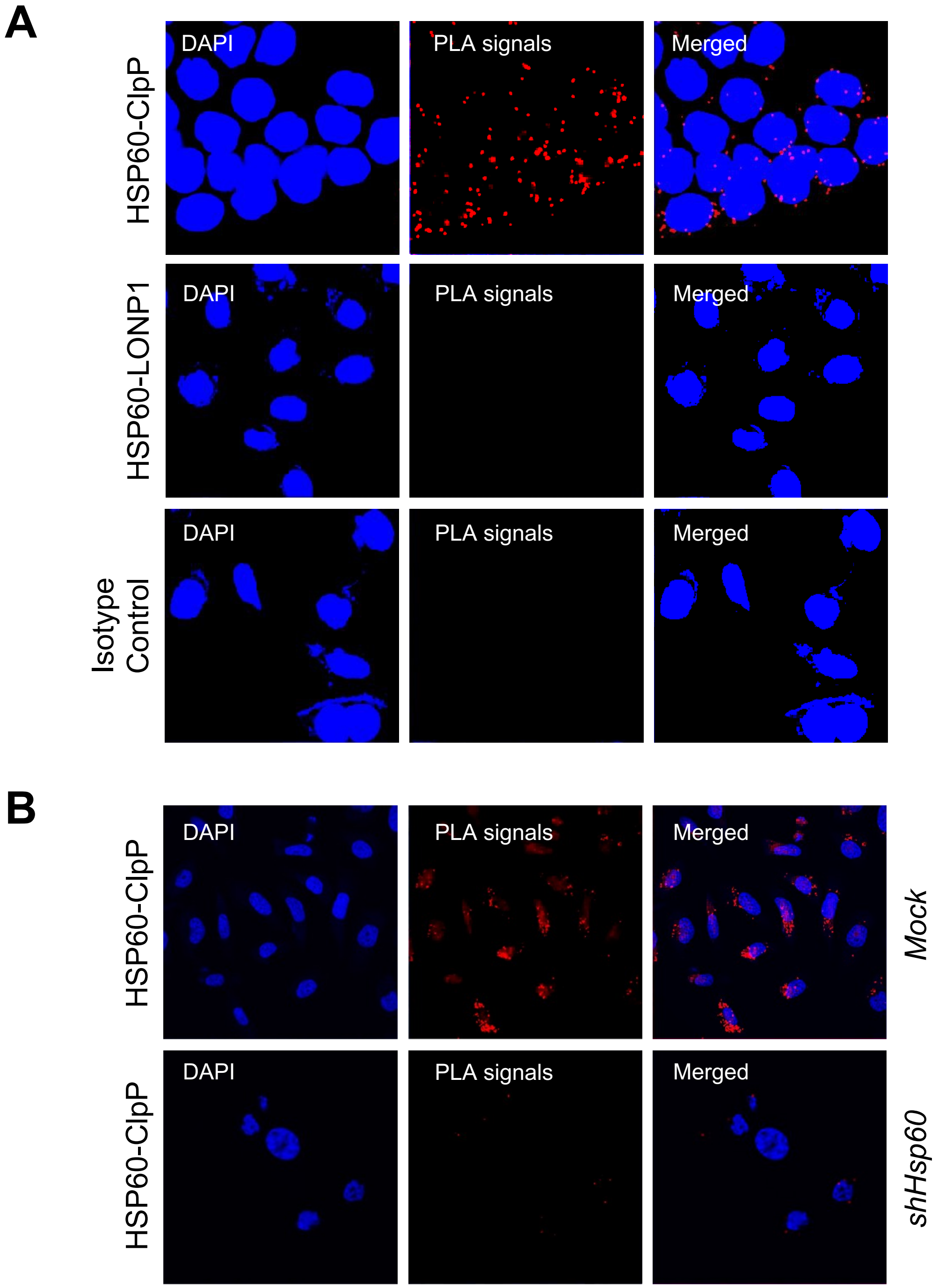

Figure S3: HSP60 interacts with ClpP but not with LONP1.
(A) HSP60-ClpP and HSP60-LONP1 interactions were analyzed in DU145 PCa cells by proximity ligation assay (PLA) as described in Materials and Methods. Isotype controls using normal mouse IgG and normal rabbit IgG antibodies serve as negative controls. **(B)** HSP60-ClpP interaction was analyzed in *Hsp60* silenced LNCaP cells by PLA.

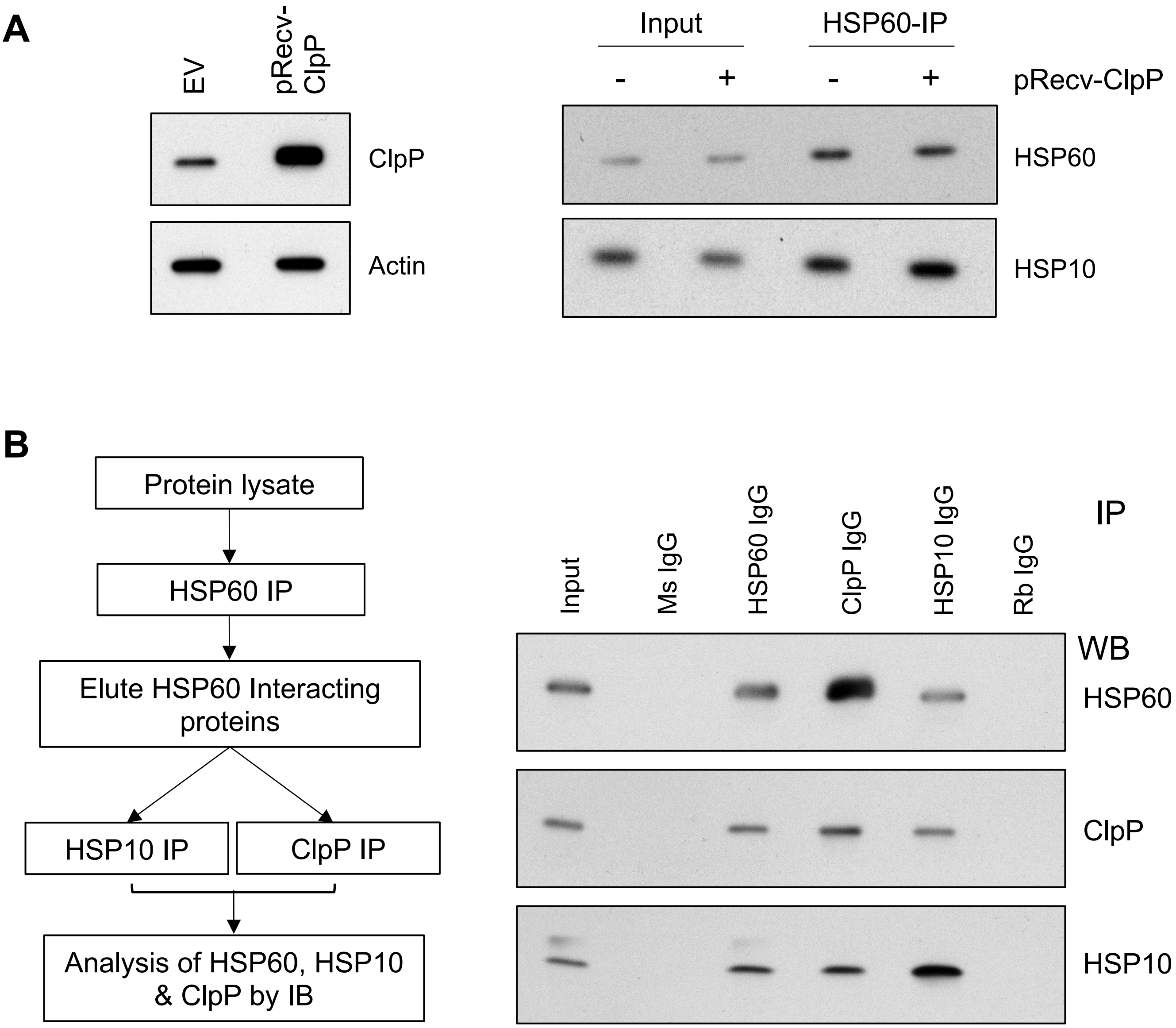

Figure S4: HSP60 interacts with both HSP10 and ClpP, whereas HSP10 and ClpP do not compete for binding with HSP60.

(A) Analysis of HSP60 interaction with HSP10 following ClpP overexpression in LNCaP cells.

(B) Double IP of HSP60 and then HSP10 or ClpP IP in eluate from HSP60 IP as described in Materials and Methods.

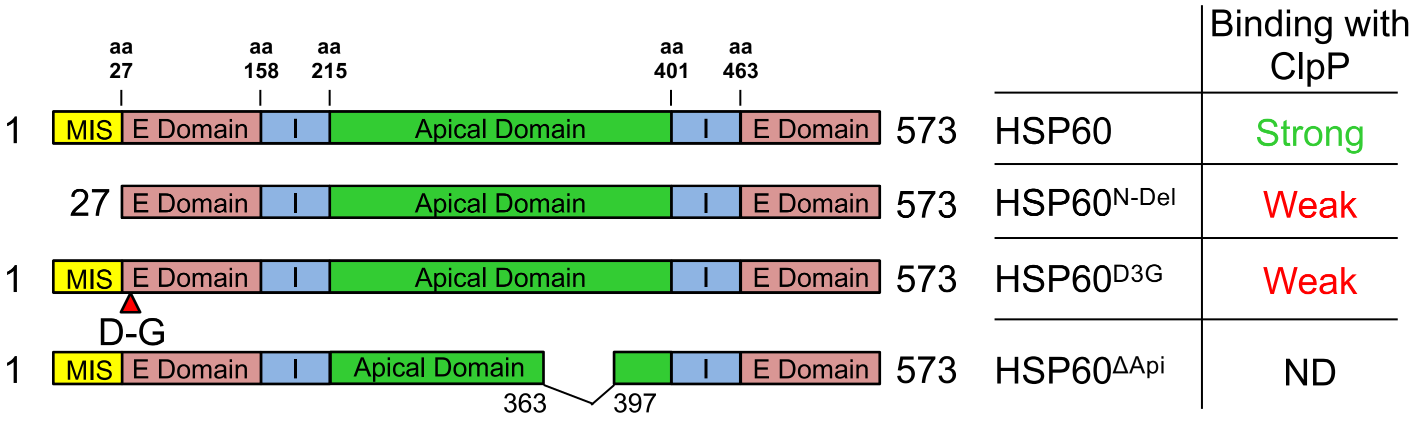

Figure S5: Schematic of various constructs of HSP60 (mutants) and their interactions with ClpP.

Various mutants of HSP60 and their interactions with ClpP were analyzed using biochemical experiments as described for Figure 3G and H. MIS, mitochondrial import signal; E Domain, equatorial domain; and I, intermediate domain; ND, not detectable.

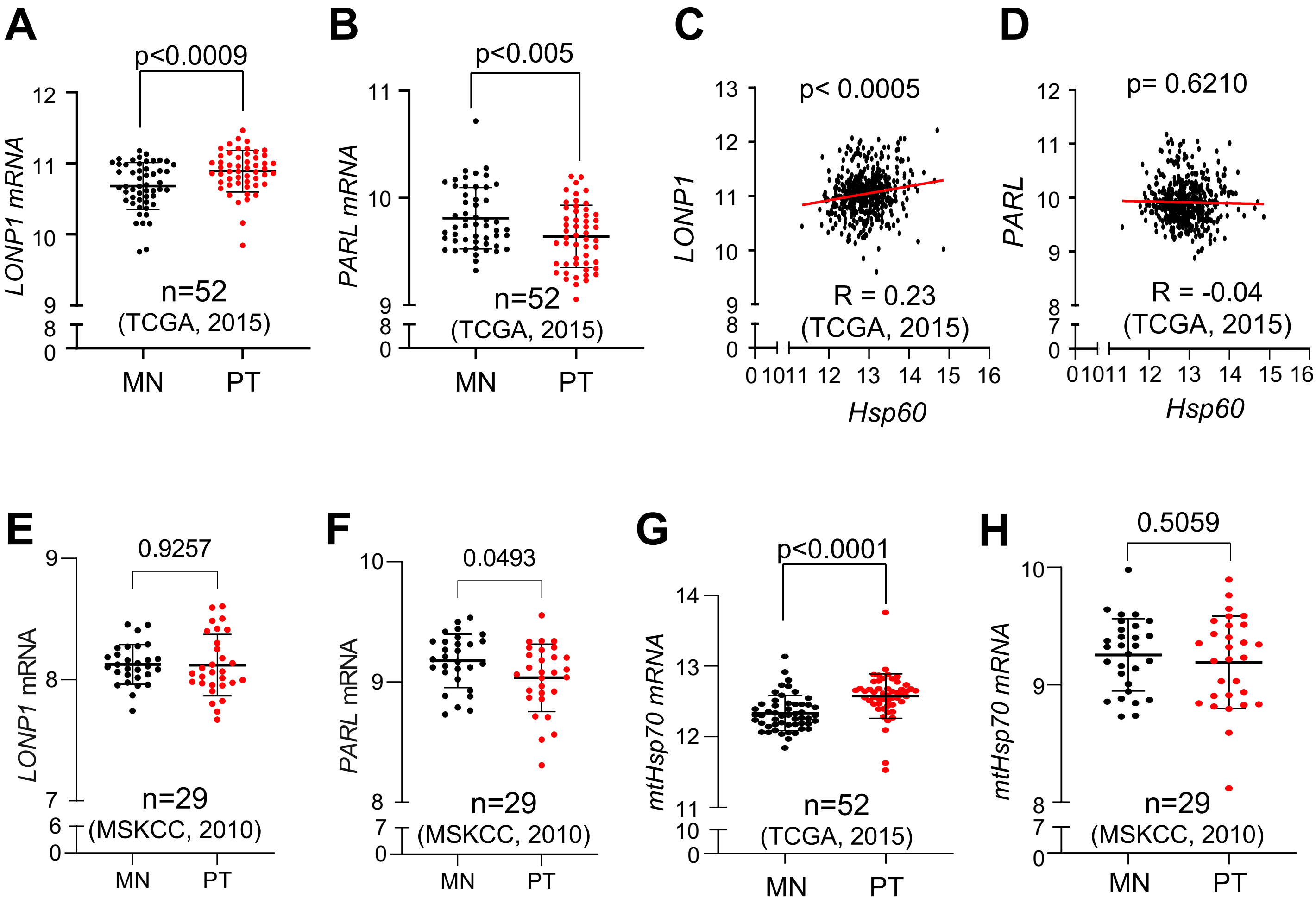

Figure S6: Transcript levels of UPR^{mt} components in prostate cancer.
(A) *LONP1* transcript reads in prostate tumors (PT) compared to matching normal (MN) tissues from TCGA dataset. **(B)** *PARL* transcript reads in PT compared to MN from TCGA dataset. **(C)** Correlative analysis between *Hsp60* and *LONP1* transcript reads from TCGA dataset. **(D)** Correlative analysis between *Hsp60* and *PARL* transcript reads from TCGA dataset. **(E)** *LONP1* transcript reads in PT compared to MN from MSKCC 2010 dataset. **(F)** *PARL* transcript reads in PT compared to MN from MSKCC 2010 dataset. **(G)** *mtHsp70* transcript reads in PT compared to MN from TCGA dataset. **(H)** *mtHsp70* transcript reads in PT compared to MN from MSKCC 2010 dataset. p values were calculated by 2-tailed t-test (**A-H**).

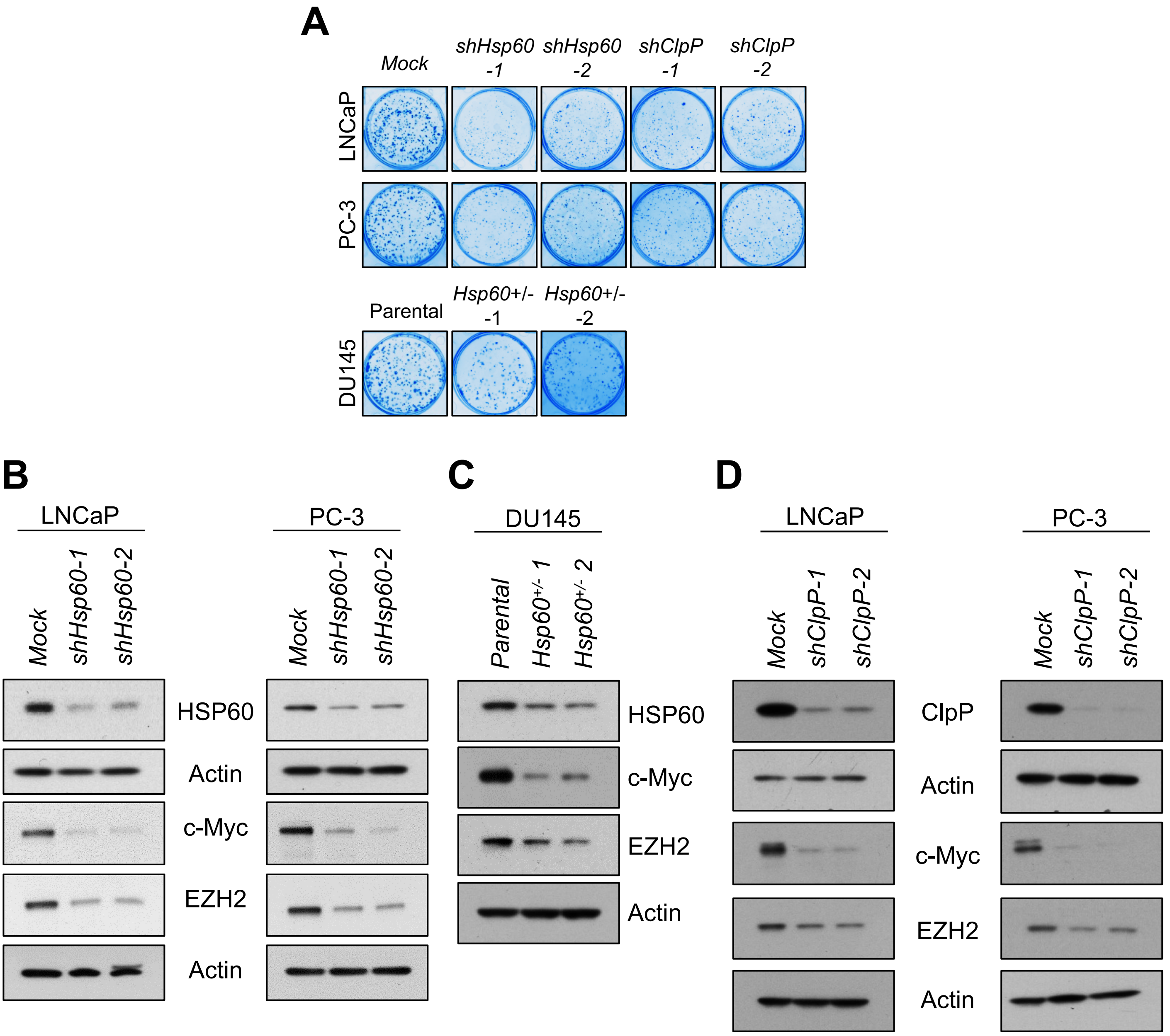

Figure S7: HSP60 or ClpP silencing inhibits clonal growth and reduces c-Myc and EZH2 in PCa cells.

(A) Clonogenic assays were performed in *Hsp60* and *ClpP* silenced LNCaP and PC-3 cells, and in DU145 *Hsp60*$^{+/-}$ cells. **(B)** Protein levels of c-Myc and EZH2 in *Hsp60* silenced LNCaP and PC-3 cells. **(C)** Protein levels of c-Myc and EZH2 in DU145 *Hsp60*$^{+/-}$ cells. **(D)** Protein levels of c-Myc and EZH2 in *ClpP* silenced LNCaP and PC-3 cells.

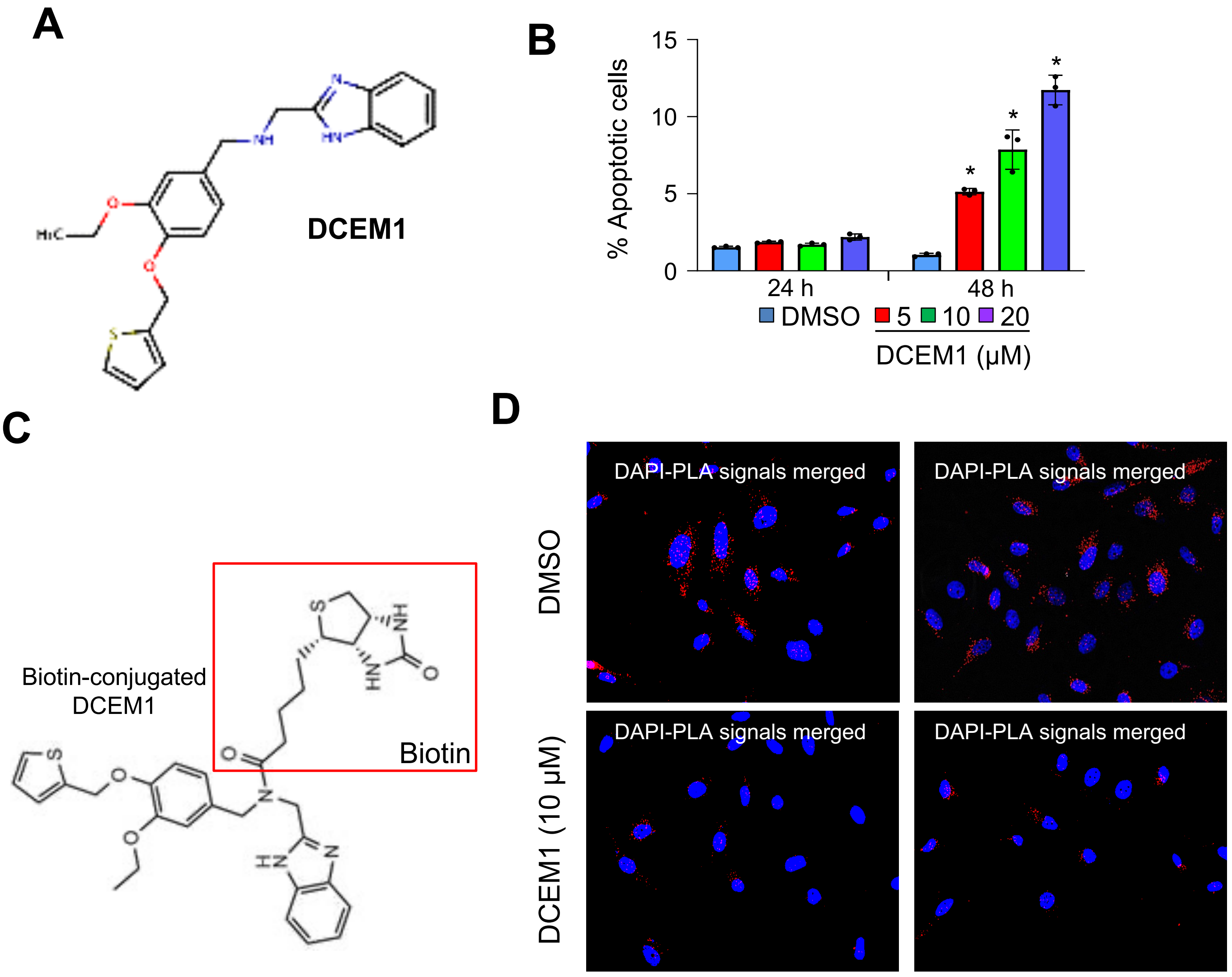

Figure S8: DCEM1 disrupts HSP60-ClpP interactions, and induces a very little or no apoptosis in immortalized untransformed prostate epithelial cells.
(A) Structure of DCEM1. **(B)** Human immortalized prostate epithelial RWPE-1 cells were treated with DCEM1 at indicated concentration for 24 and 48 h and apoptotic cells were quantified by Annexin V/PI labeling and results presented as fold change compared to DMSO controls. Data are mean ± SD (n=3). *p<0.05 by 1-way ANOVA followed by Dunnett's multiple-comparison tests. **(C)** Structure of biotin-conjugated DCEM1. **(D)** PC-3 cells were treated with DCEM1 (10 μM) for 24 h and HSP60-ClpP interaction was analyzed by proximity ligation assay (PLA). Images are from two separate areas under microscope.

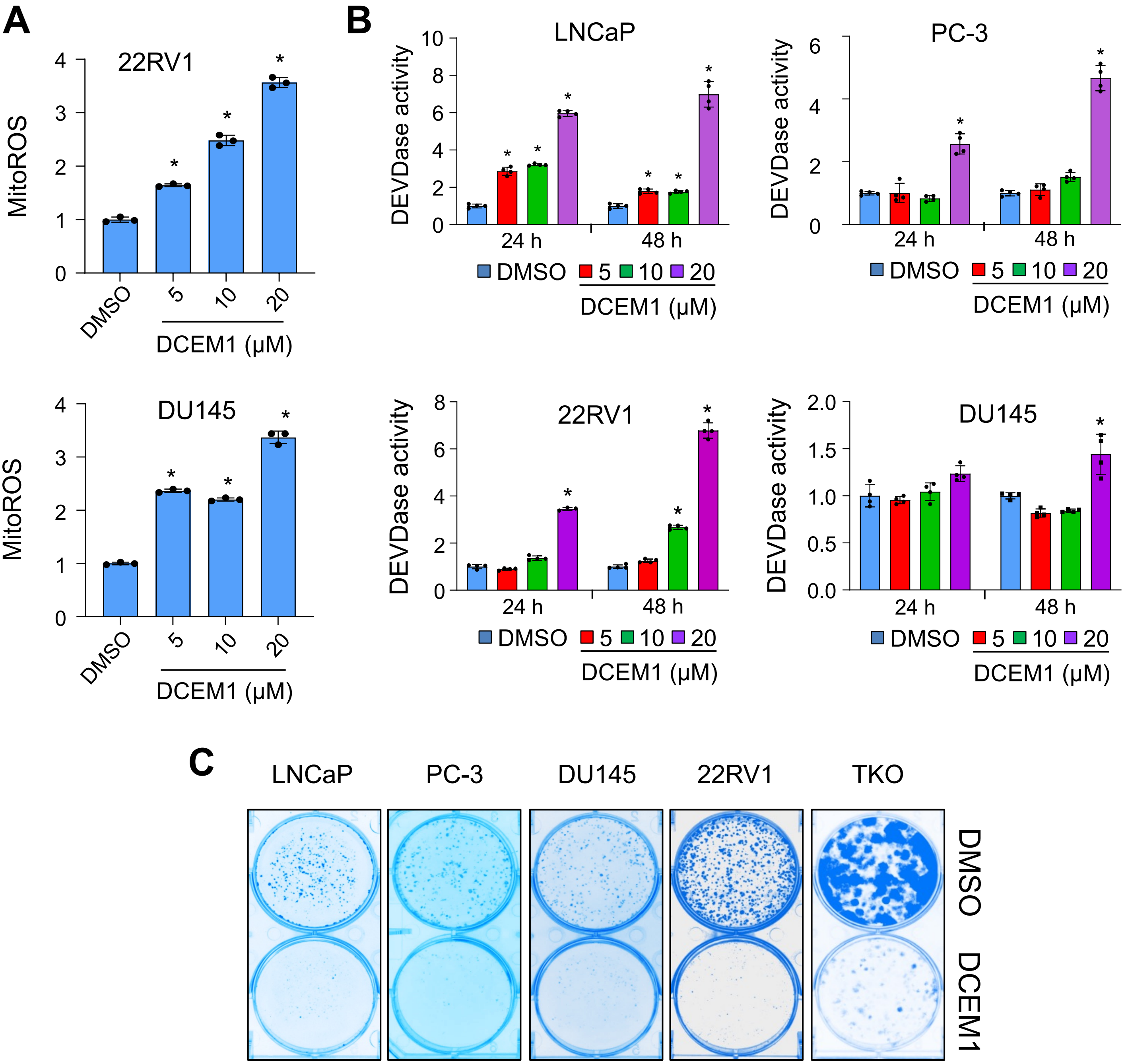

Figure S9: DCEM1 induces mitochondrial ROS (mitoROS) production and caspase activation, and inhibits clonogenicity in PCa cells.
(A) 22RV1 and DU145 cells were treated with DCEM1 at indicated doses for 24 h and mitoROS levels were analyzed by flow cytometry. **(B)** LNCaP, PC-3, 22RV1 and DU145 cells were treated with DCEM1 at indicated doses for 24 and 48 h and DEVDase activity analyzed. Results are presented as fold changes compared to respective controls. **(C)** LNCaP, PC-3, DU145, 22RV1, and mouse TKO PCa cells were treated with 5 µM DCEM1 and after 6 days clones were stained with crystal violet. Data are mean ± SD (n=3). **p*<0.05 by 1-way ANOVA followed by Dunnett's multiple-comparison tests (**A** and **B**).

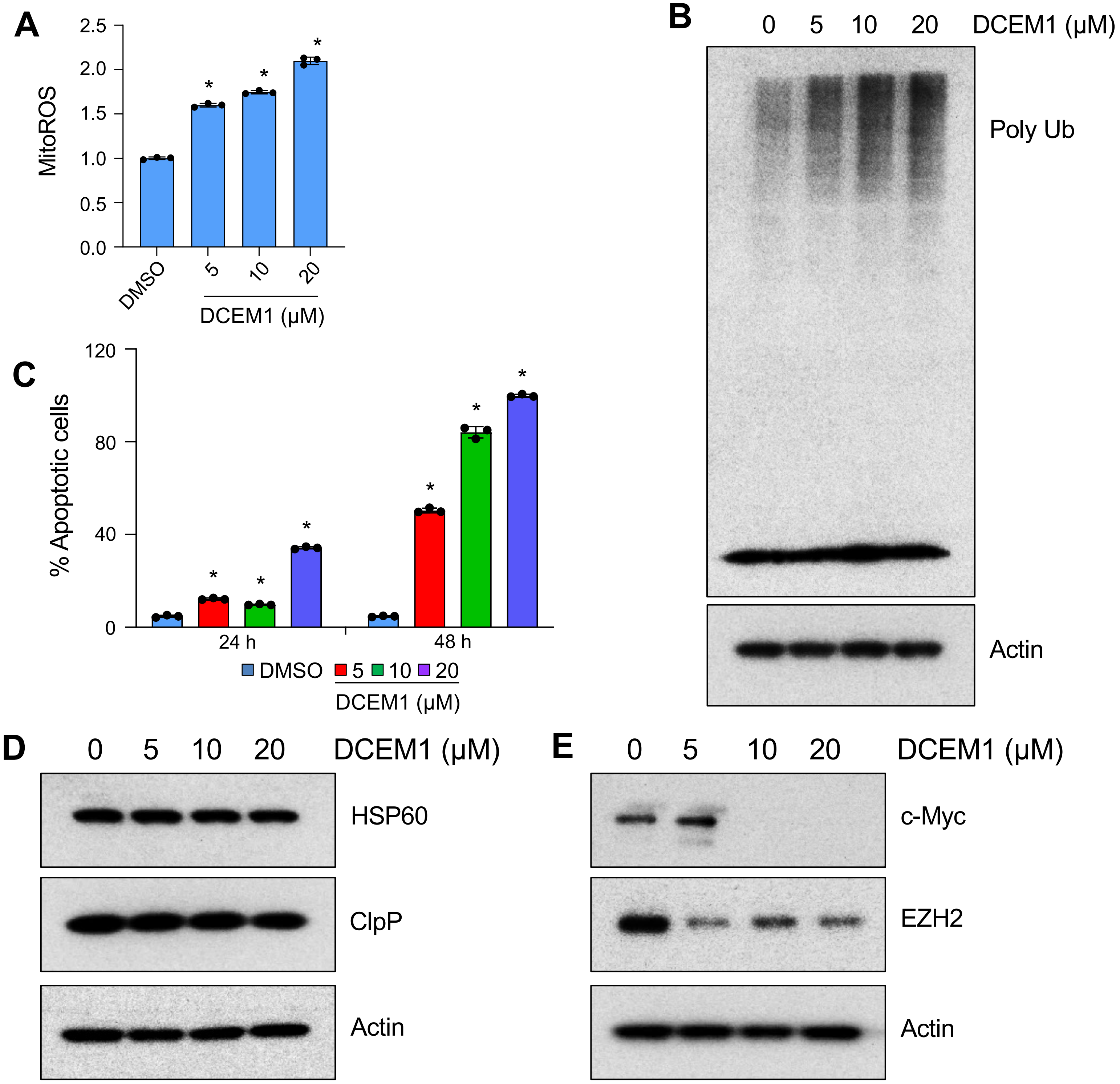

Figure S10: DCEM1 inhibits c-Myc and EZH2, and induces mitoROS, polyubiquitination, and apoptosis in TKO cells.

(A) Mouse TKO PCa (TKO) cells were treated with DCEM1 at indicated doses for 24 h. MitoROS levels were analyzed by flow cytometry as described in Materials and Methods. **(B)** TKO cells were treated with DCEM1 at indicated doses for 24 h and polyubiquitination (Poly Ub) levels were analyzed by Western blotting. Actin serves as a loading control. (**C-E)** TKO cells were treated with DCEM1 at indicated doses for 24 and 48 h and apoptotic cells were quantified by Annexin V/PI staining and FACS (**C**). TKO cells were treated with DCEM1 at indicated doses for 24 h. HSP60 and ClpP levels **(D)** and c-Myc and EZH2 levels **(E)** were analyzed by Western blotting. Actin serves as a loading control. Data are mean ± SD (n=3). *$p<0.05$ by 1-way ANOVA followed by Dunnett's multiple-comparison tests (**A** and **C**).

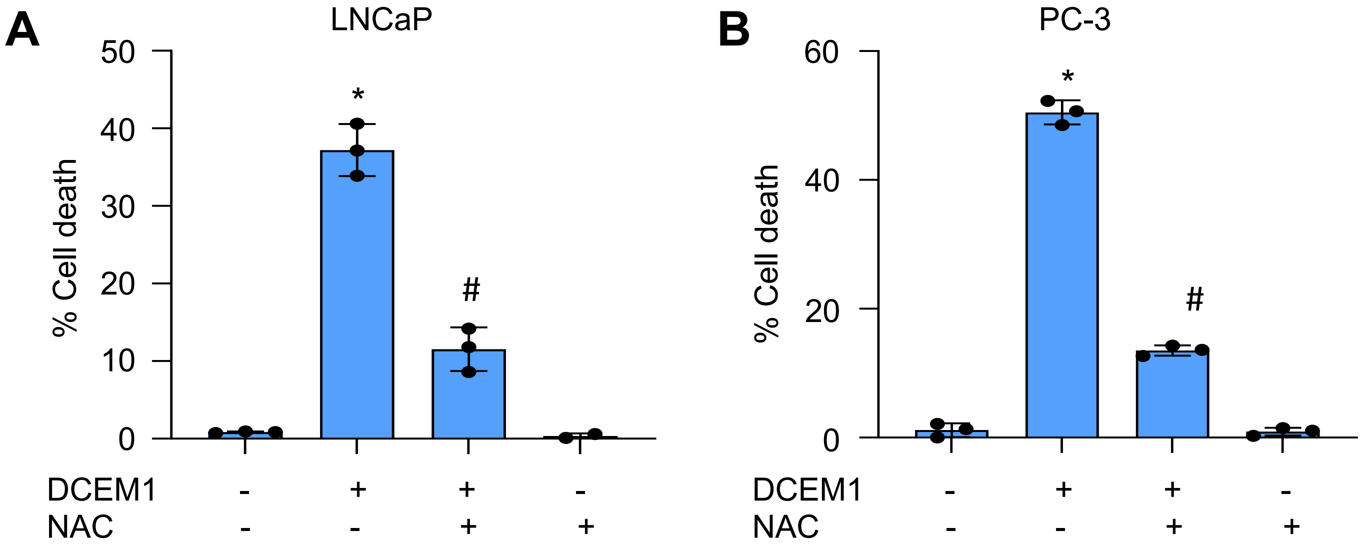

Figure S11: N-acetyl cysteine (NAC) antagonizes DCEM1-induced cell death.
LNCaP **(A) and** PC-3 **(B)** PCa cells were pretreated with 10 mM NAC for 2 h followed by treatment with DCEM1 (10 µM) for 24 h and cell death was analyzed by lactate dehydrogenase (LDH) release assay. Data are mean ± SD (n=3). * $p<0.05$ compared to untreated cells, # $p<0.05$ compared to DCEM1 treated cells by 1-way ANOVA followed by Tukey's multiple-comparisons test (**A** and **B**).

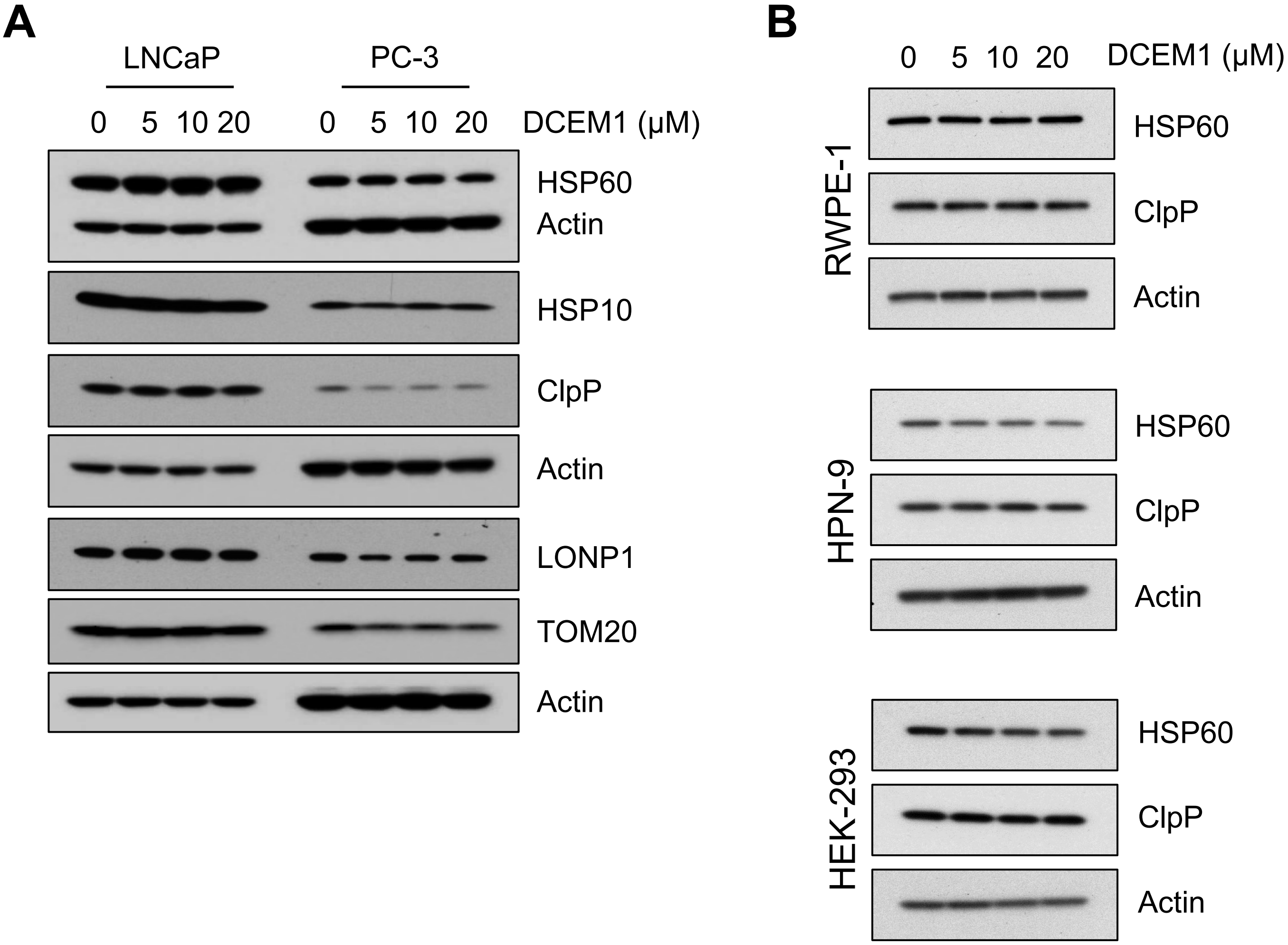

Figure S12: The components of UPR^{mt} were not affected by DCEM1 treatment with exception in PC-3 cells.

(A) LNCaP and PC-3 cells were treated with DCEM1 for 24 hours and UPR^{mt} components were analyzed by Western blotting. Actin serves as a loading control. **(B)** Immortalized prostate epithelial cells (RWPE1), normal human prostate epithelial cells (HPN-9), and HEK-293 cells were treated with DCEM1 for 24 hours and UPR^{mt} components were were analyzed by Western blotting. Actin serves as a loading control.

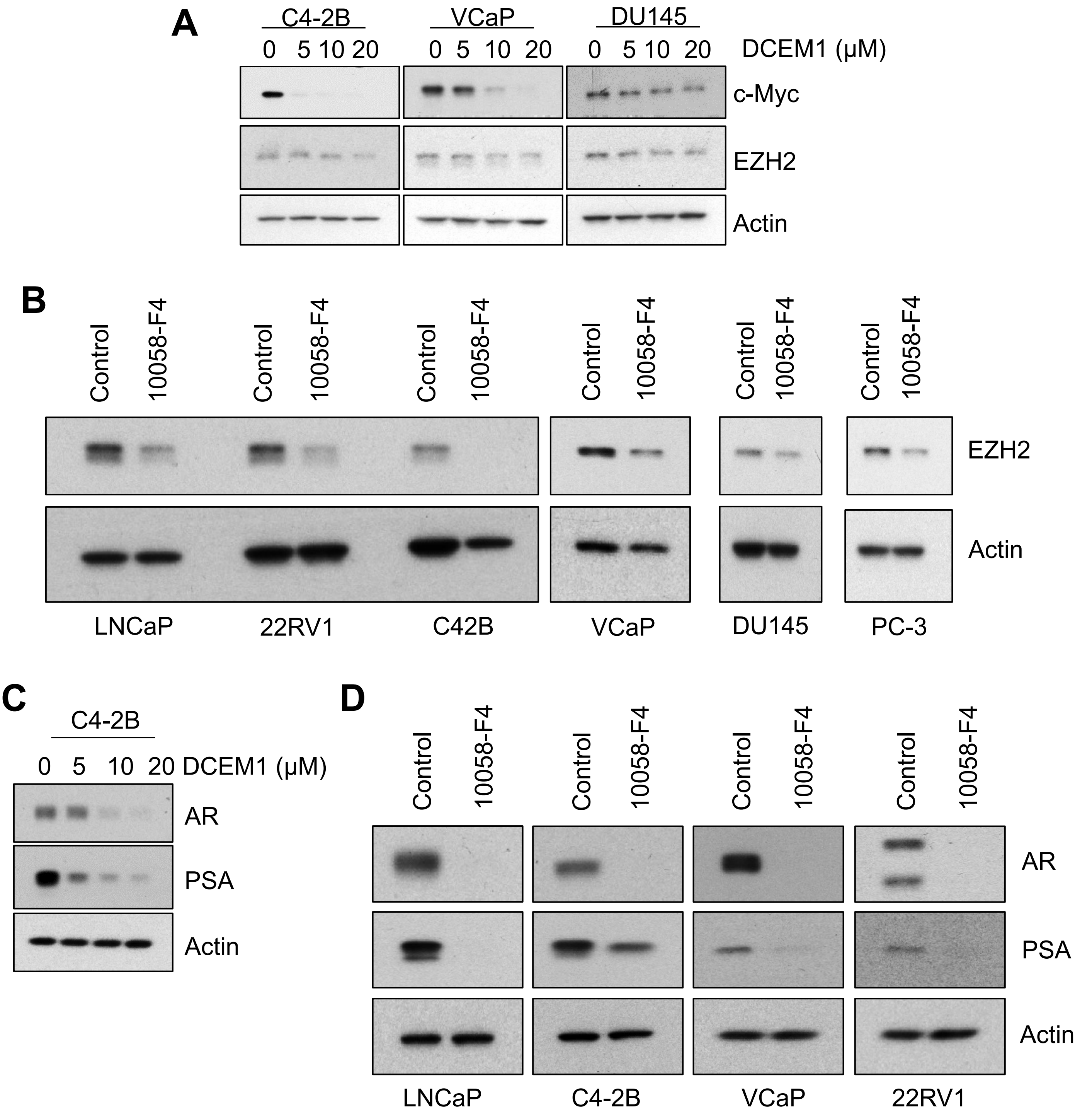

Figure S13: DCEM1 and c-Myc inhibitor reduced expression of EZH2 and AR in PCa cells. **(A)** C4-2B, VCaP and DU145 PCa cells were treated with indicated doses of DCEM1 for 24 h and c-Myc and EZH2 protein expression levels were analyzed in whole cell lysates. **(B)** PCa cells were treated with 75 μM 10058-F4 (c-Myc inhibitor) for 24 h and EZH2 protein expression levels were analyzed in whole cell lysates. **(C)** C4-2B cells were treated with indicated doses of DCEM1 for 24 h and AR and PSA protein expression levels were analyzed in whole cell lysates. **(D)** AR positive PCa cells were treated with 75 μM 10058-F4 (c-Myc inhibitor) for 24 h and AR and PSA protein expression levels were analyzed in whole cell lysates. Actin serves as a loading control in all panels.

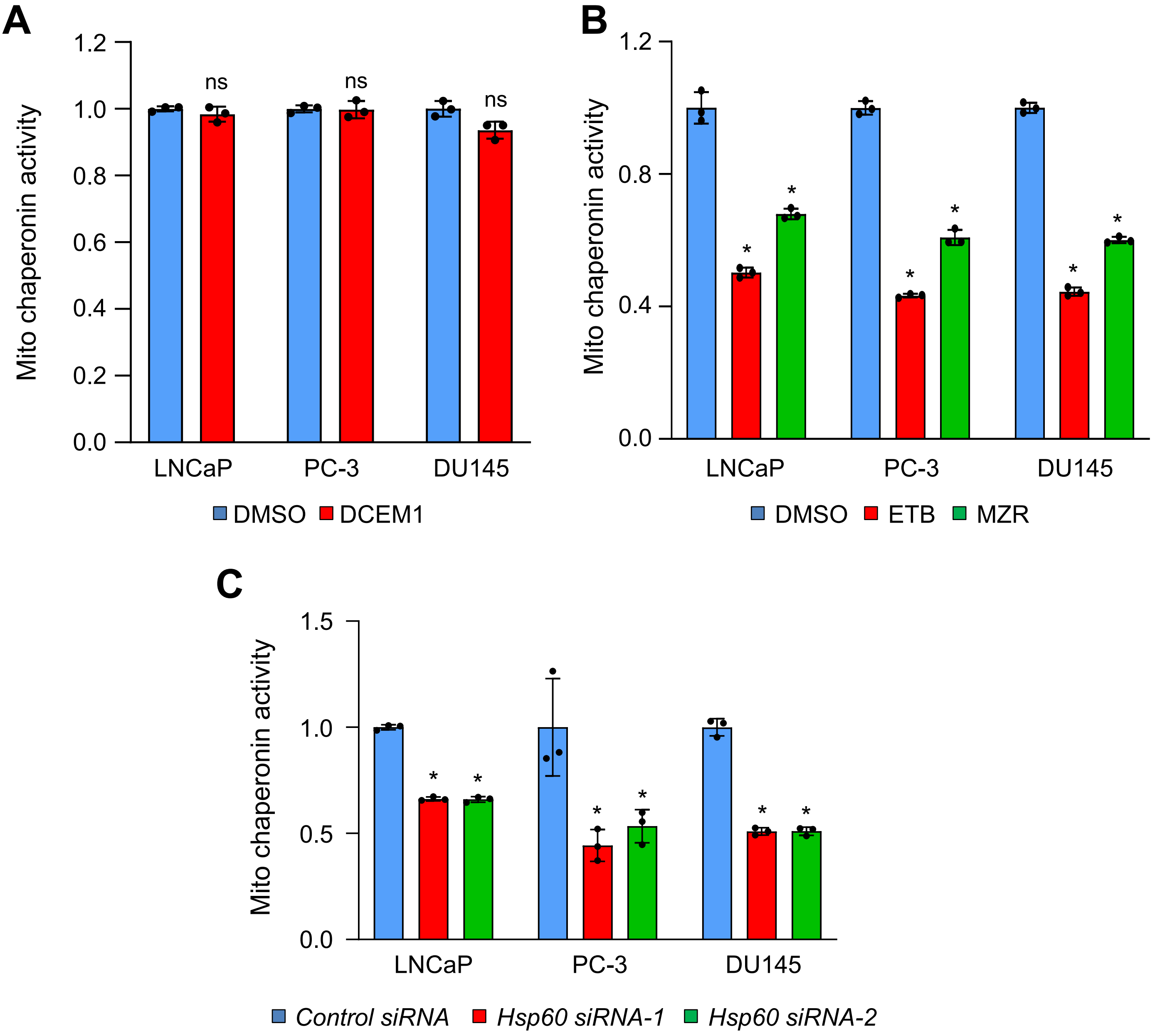

Figure S14: <u>Mi</u>tochondrial <u>C</u>haperonin <u>A</u>ctivity <u>A</u>ssay (MiCAA).
PCa cells were treated with either DCEM1 **(A)** or ETB or Mizoribine **(B)** for 24 h and mitochondrial (Mito) chaperonin activity was analyzed as described in Materials and Methods. *Hsp60* silenced PCa cells by siRNA transfection were used for mitochondrial chaperonin activity **(C)**. Data are mean ± SD (n=3). * $p<0.05$ compared to respective controls by 1-way ANOVA followed by Dunnett's multiple-comparison test (**A-C**).

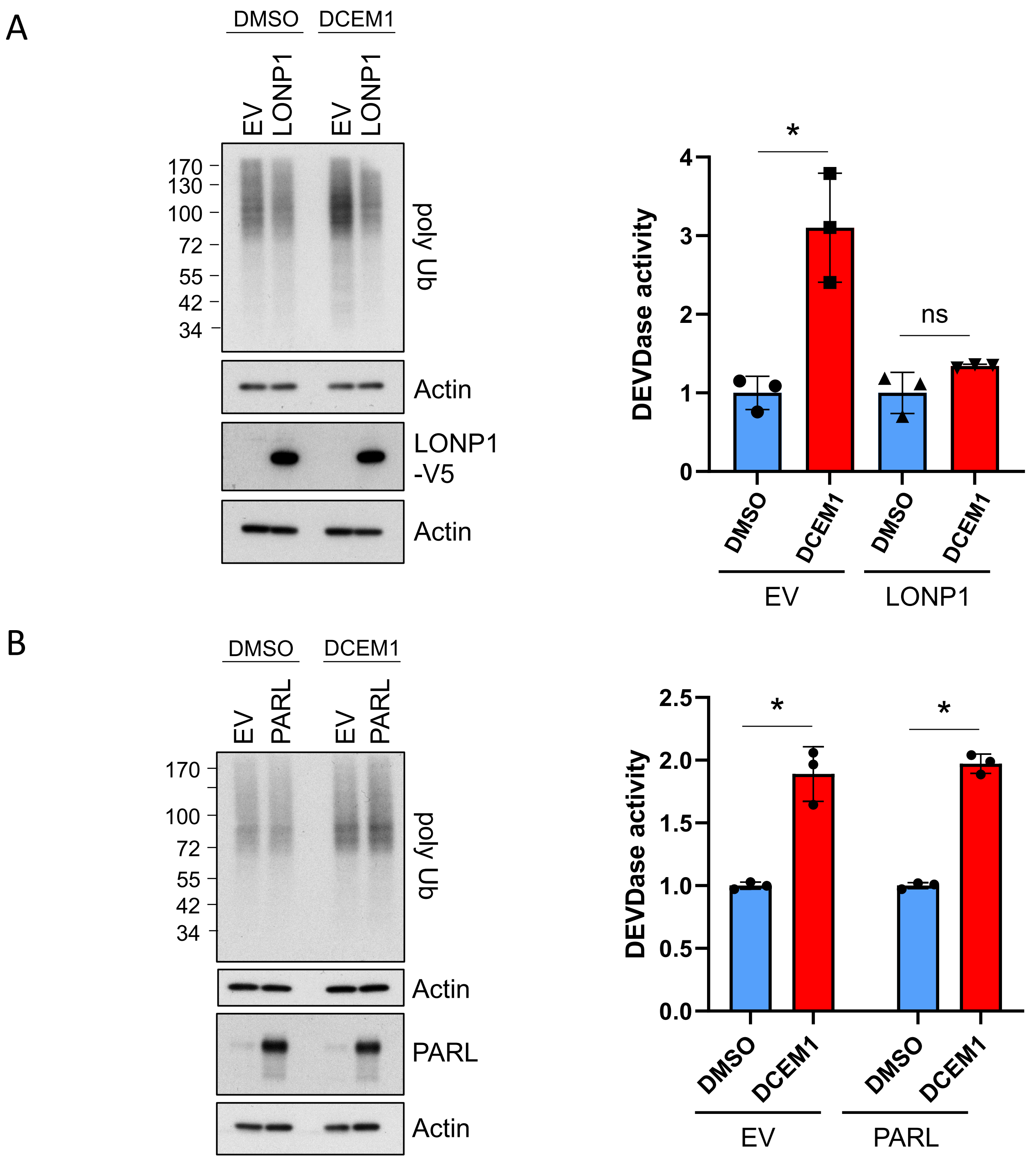

Figure S15: LONP1 but not PARL inhibits DCEM1-induced accumulation of poly-Ub proteins and DEVDase activity.

(A) LONP1 protein was over-expressed in LNCaP cells followed by DCEM1 (10 μM) treatment and level of poly-ubiquitinated proteins and DEVDase activity was analyzed. Actin serves as a loading control. (B) PARL protein was over-expressed in LNCaP cells followed by DCEM1 (10 μM) treatment and level of poly-ubiquitinated proteins and DEVDase activity was analyzed. Actin serves as a loading control. Data are mean ± SD (n=3). * $p<0.05$ compared to respective controls by 1-way ANOVA followed by Tukey's multiple-comparison test (**A** and **B**).

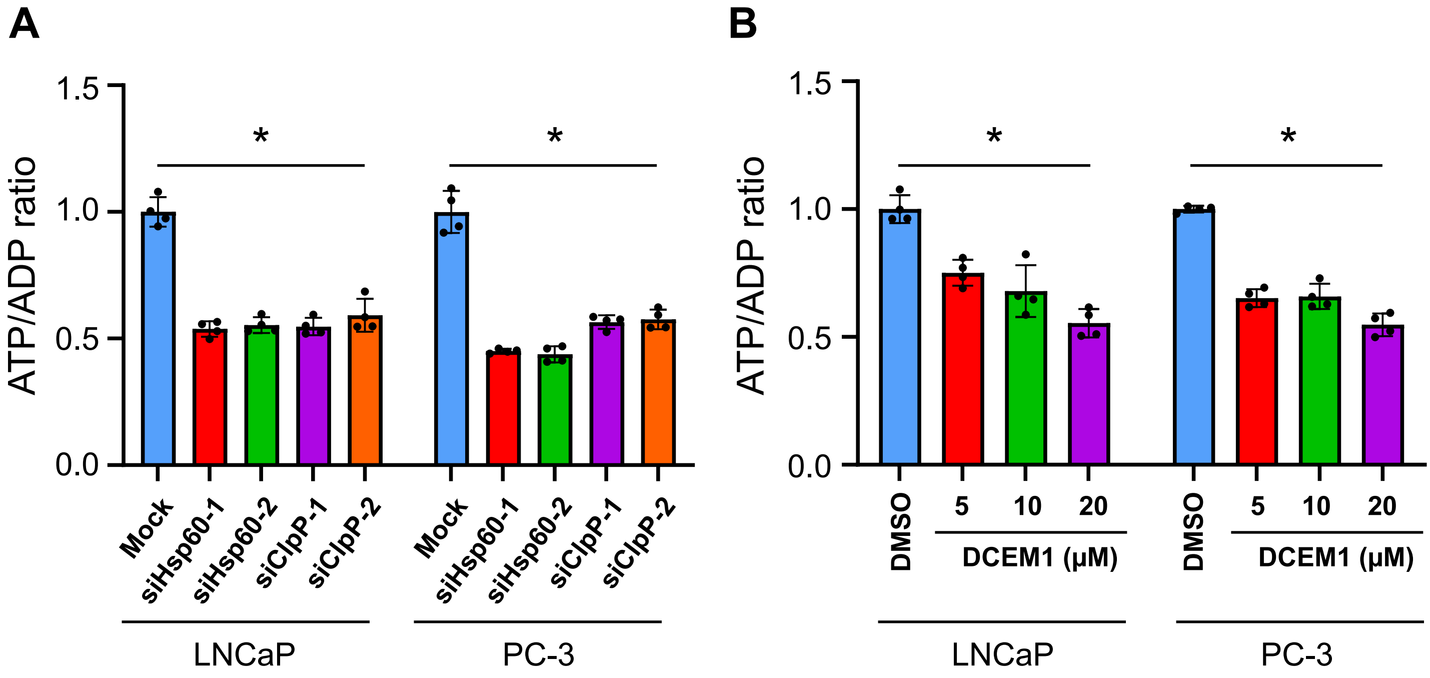

Figure S16: Reduced ATP/ADP ratio upon *Hsp60* or *ClpP* silencing and by DCEM1 treatment in PCa cells.
(A) LNCaP and PC-3 cells were transfected with either *Hsp60* or *ClpP* siRNA and ATP/ADP ratio was analyzed after 72 h. **(B)** LNCaP and PC-3 cells were treated with indicated doses of DCEM1 and ATP/ADP ratio was analyzed after 24 h. Data are mean ± SD (n=4). * $p<0.05$ by 1-way ANOVA followed by Dunnett's multiple-comparison test (**A** and **B**).

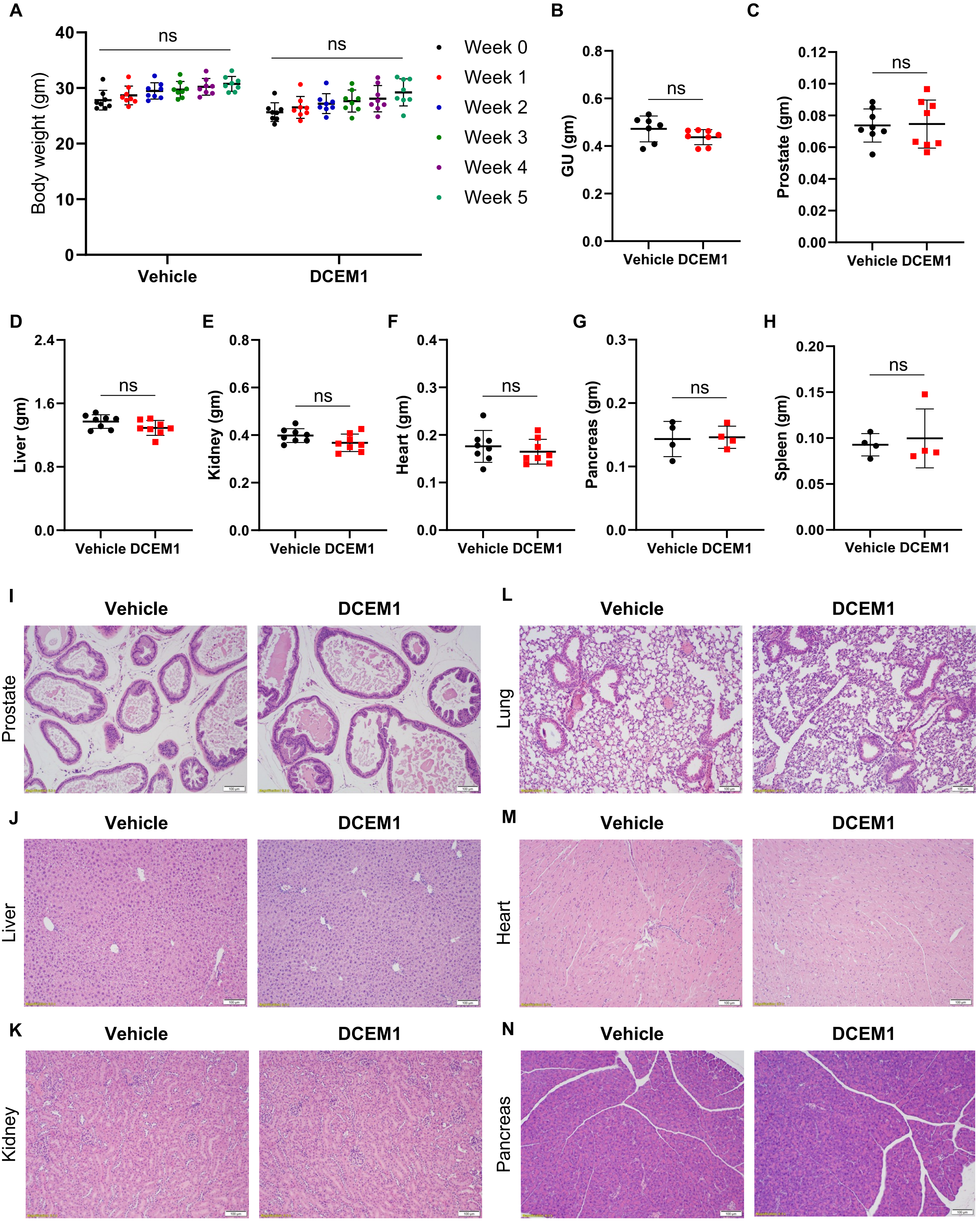

Figure S17: DCEM1 treatment did not cause organ or systemic toxicities.
C57BL/6 nontumor bearing mice were treated with either vehicle or DCEM1 (60 mg/kg body weight) twice weekly for 6 weeks. Body weight were recorded on a weekly basis **(A)**. At termination, major organs were harvested, weighed **(B-H)** and sections were cut and stained with H&E for histopathological evaluation **(I-N)**. Presented images were captured using 20x objective. Data are mean ± SD. ns- not significant by 2-tailed t test (**A-H**).

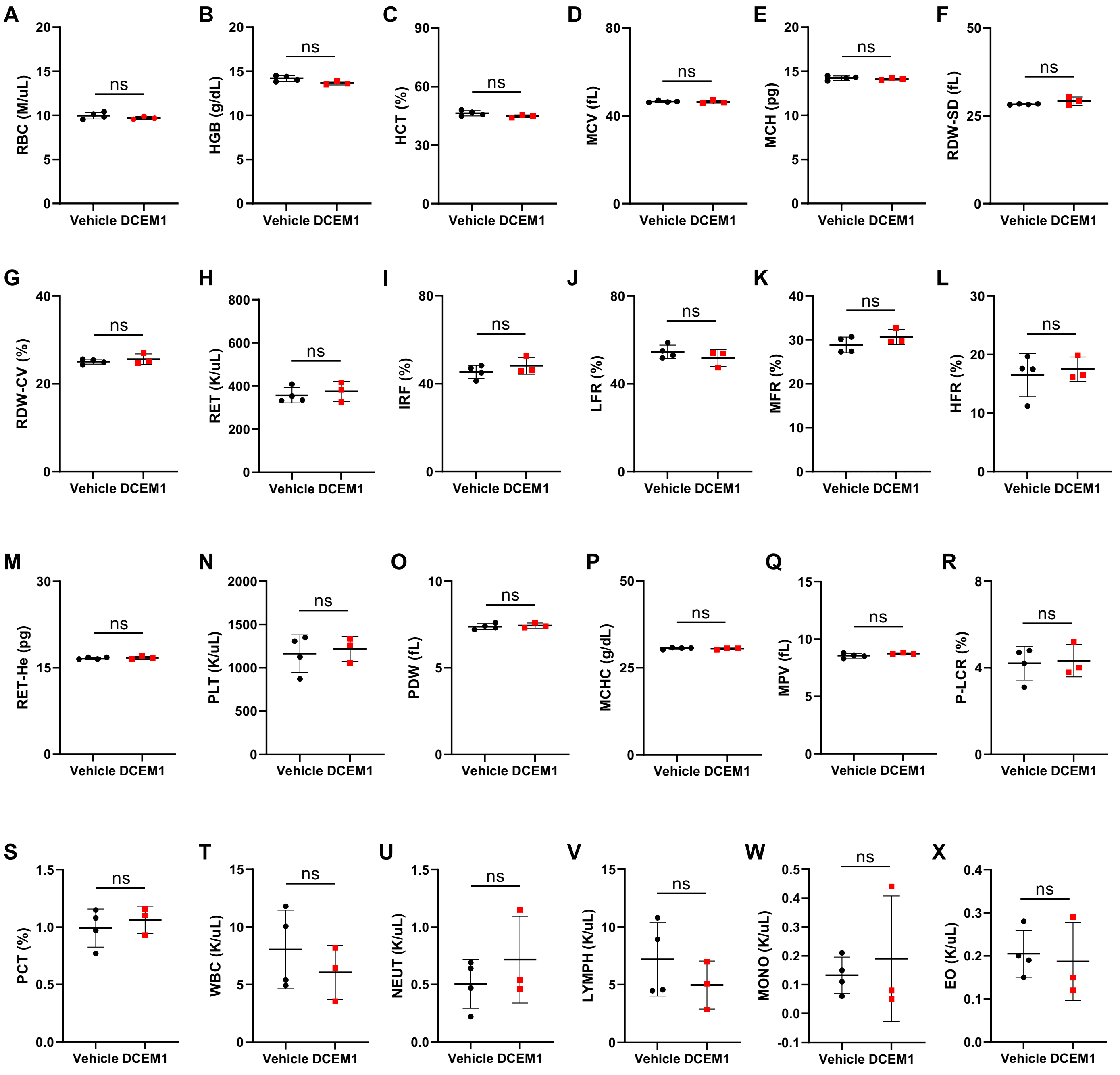

Figure S18: DCEM1 treatment of C57BL/6 mice did not change hematological parameters. Shown are hematological parameters measured in blood from vehicle (n=4) and DCEM1 (n=3) treated non-tumor bearing mice. **(A)** RBC, Red blood cells; **(B)** HGB, Hemoglobin; **(C)** HCT, Hematocrit; **(D)** MCV, Mean corpuscular volume; **(E)** MCH, Mean corpuscular hemoglobin; **(F)** RDW-SD, Red cell distribution width SD; **(G)** RDW-CV, Red cell distribution width CV; **(H)** RET, Reticulocyte count; **(I)** IRF, Immature reticulocyte fraction; **(J)** LFR, Low fluorescence reticulocytes; **(K)** MFR, Medium fluorescence reticulocytes; **(L)** HFR, High fluorescence reticulocytes; **(M)** RET-He, Reticulocyte hemoglobin count; **(N)** PLT, Platelet count; **(O)** PDW, Platelet distribution width; **(P)** MCHC, Mean corpuscular hemoglobin concentration; **(Q)** MPV, Mean platelet volume; **(R)** P-LCR, Platelet large cell ratio; **(S)** PCT, Plateletcrit; **(T)** WBC, White blood cell count; **(U)** NEUT, Neutrophil; **(V)** LYMPH, Lymphocyte; **(W)** MONO, Monocyte; **(X)** EO, Eosinophil. Data are mean ± SD, ns, not significant by 2-tailed t test (**A-X**).

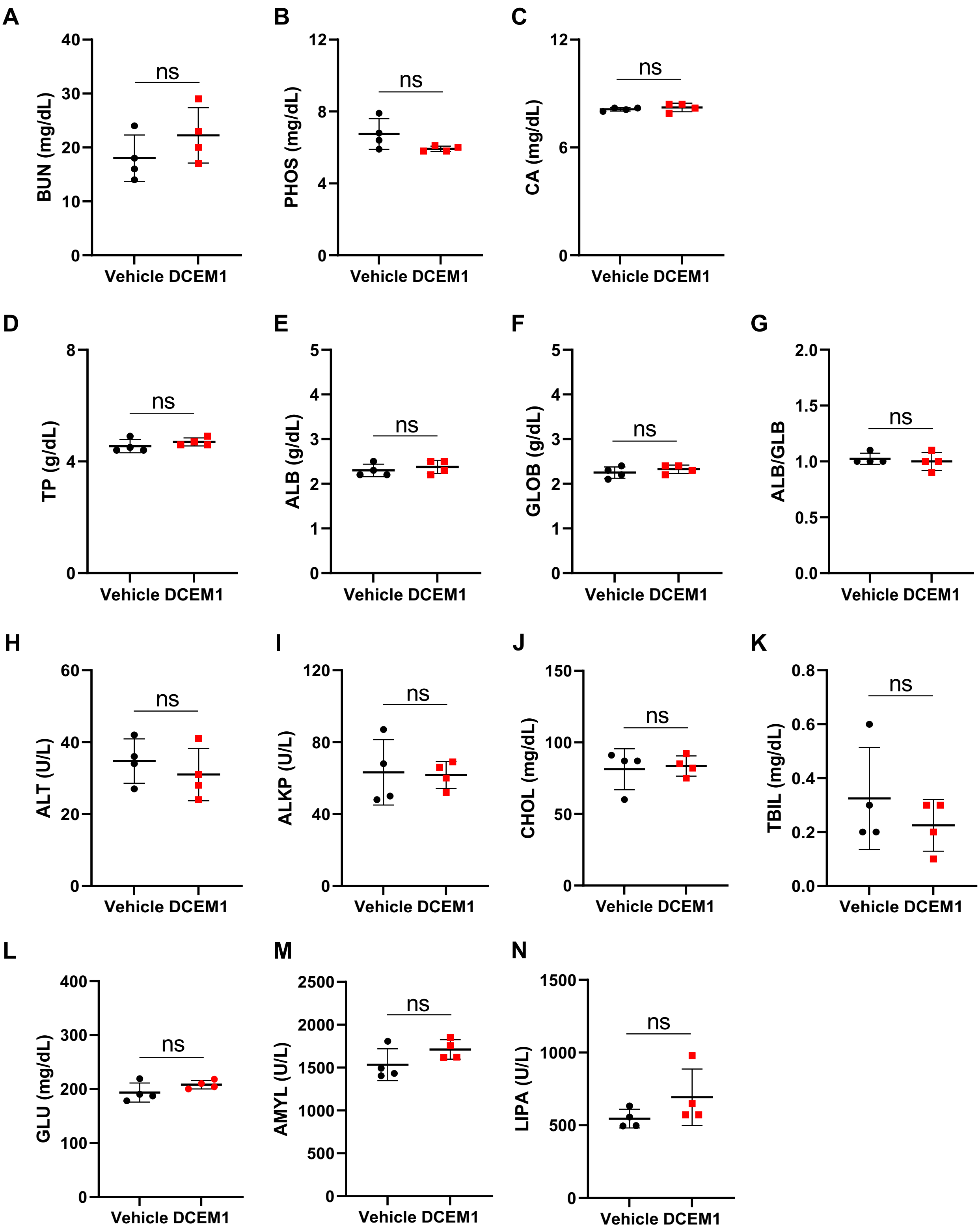

Figure S19: DCEM1 treatment of C57BL/6 mice did not change clinical chemistry parameters. Shown are 14 clinical chemistry parameters in blood from vehicle or DCEM1 treated mice. **(A)** BUN, Blood urea nitrogen; **(B)** PHOS, Inorganic phosphorus; **(C)** CA, Calcium; **(D)** TP, Total protein; **(E)** ALB, Albumin; **(F)** GLOB, Globulin; **(G)** ALB/GLOB ratio; **(H)** ALT, Alanine aminotransferase; **(I)** ALKP, Alkaline phosphatase; **(J)** Cholesterol (total); **(K)** TBIL, Bilirubin (total); **(L)** GLU, Glucose; **(M)** AMYL, Amylase; **(N)** LIPA, Lipase. Data are mean ± SD (n=4), ns, not significant by 2-tailed t test (**A-N**)

Supplementary Table S1: List of antibodies used in the study

Reagent or Resource	Source	Identifier
Antibody		
HSP60 (Clone LK1)	Millipore	Cat# 386028-50UG
HSP60 (Clone D6F1)	Cell Signaling	Cat# 12165
ClpP	Sigma	Cat# HPA010649
ClpP (Clone B-12)	Santa Cruz Biotechnology	Cat# sc-271284
HSP10	Enzo	Cat# SPA-110E
GAPDH (Clone 7B)	Santa Cruz Biotechnology	Cat# sc-69778
c-Myc (Clone D3N8F)	Cell Signaling	Cat# 13987
Actin-HRP (Clone C4)	Santa Cruz Biotechnology	Cat# sc-47778 HRP
EZH2 (Clone D2C9)	Cell Signaling	Cat# 5246
Ki67 (Clone MIB-1)	Agilent	Cat# M7249
TOM20 (Clone D8T4N)	Cell Signaling	Cat# 42406
LDHB (Clone EP1565Y)	Abcam	Cat# ab53292
TBP	Proteintech	Cat# 22006-1-AP
Normal Ms IgG	Santa Cruz Biotechnology	Cat# sc-2025
Normal Rb IgG	Santa Cruz Biotechnology	Cat# sc-2027
V5 (Clone C-9)	Santa Cruz Biotechnology	Cat# sc-271944
FLAG (Clone M2)	Sigma	Cat# F1804
Ubiquitin (Clone P4D1)	Cell Signaling	Cat# 3936
LONP1	Abcam	Cat# ab103809
NDUFA9 (Clone 20C11B11B11)	Abcam	Cat# ab14713
SDHA (Clone 2E3GC12FB2AE2)	Abcam	Cat# ab14715
UQCRC2 (Clone 13G12AF12BB11)	Abcam	Cat# ab14745
COX IV (Clone 3E11)	Cell Signaling	Cat# 4850
ATP5A (Clone 15H4C4)	Abcam	Cat# ab14748
p-AMPKα (Thr172; Clone 40H9)	Cell Signaling	Cat# 2535
AMPKα (Clone D63G4)	Cell Signaling	Cat# 5832
p-ULK1 (Ser555; Clone D1H4	Cell Signaling	Cat# 5869
p-ULK1 (Ser757)	Cell Signaling	Cat# 6888
ULK1 (Clone D8H5)	Cell Signaling	Cat# 8054
p-Akt (Ser473; Clone D9E)	Cell Signaling	Cat# 4060
Akt (Clone 11E7)	Cell Signaling	Cat# 4685

p-mTOR (Ser2448; Clone D9C2)	Cell Signaling	Cat# 5536
mTOR (Clone 7C10)	Cell Signaling	Cat# 2983
p-p70 S6 (Ser371)	Cell Signaling	Cat# 9208
p-p70 S6 (Thr389; Clone 108D2)	Cell Signaling	Cat# 9234
p-4E-BP1 (Thr37/46; Clone 236B4)	Cell Signaling	Cat# 2855
AR (N-20)	Santa Cruz Biotechnology	Cat# sc-816
PSA (Clone D6B1)	Cell Signaling	Cat# 5365
Anti-rabbit IgG, HRP	Cell Signaling	Cat# 7074
Anti-mouse IgG, HRP	Cell Signaling	Cat# 7076
Anti-mouse IgG, Alexa Fluor 488	ThermoFisher Scientific	Cat# A-11001
Anti-rabbit IgG, Alexa Fluor 594	ThermoFisher Scientific	Cat# A-11037

Supplementary Table S2: List of shRNAs and their sequences

**shRNA**	**Mature Antisense Sequence**
Hsp60 shRNA1	*5'-CGCAATGACCATTGCTAAGAAT-3'*
Hsp60 shRNA2	*5'-AGCTATATTTCTCCATACTTTA-3'*
ClpP shRNA1	*5'-GCTCAAGAAGCAGCTCTATAA-3'*
ClpP shRNA2	*5'-AGAAGCAGCTCTATAACATCT-3'*

Lentiviral particles specific for *Hsp60* and *ClpP*, and *control shRNAs* were obtained from the Roswell Park Comprehensive Cancer Center shRNA core resource and were directly utilized to infect cells at a multiplicity of infection (MOI) of 2.

Supplementary Table S3: List of siRNAs and their sources

**siRNA**	**Source**	**Catalog Number**
Control siRNA-A	Santa Cruz Biotechnology	Cat # sc-37007
c-Myc siRNA	Santa Cruz Biotechnology	Cat # sc-29226
